# Comparative gene expression study and pathway analysis of the human iris- and the retinal pigment epithelium

**DOI:** 10.1371/journal.pone.0182983

**Published:** 2017-08-21

**Authors:** Anna Bennis, Jacoline B. ten Brink, Perry D. Moerland, Vivi M. Heine, Arthur A. Bergen

**Affiliations:** 1 Department of Clinical Genetics, Academic Medical Center, Amsterdam, The Netherlands; 2 The Netherlands Institute for Neuroscience (NIN-KNAW), Royal Netherlands Academy of Arts and Sciences, Amsterdam, The Netherlands; 3 Bioinformatics Laboratory, Department of Clinical Epidemiology, Biostatistics and Bioinformatics, Academic Medical Center, Amsterdam, The Netherlands; 4 Department of Pediatrics / Child Neurology, Neuroscience Campus Amsterdam, VU University Medical Centre, Amsterdam, The Netherlands; 5 Department of Complex Trait Genetics, Center for Neurogenomics and Cognitive Research, Neuroscience Campus Amsterdam, Vrije Universiteit, Amsterdam, The Netherlands; 6 Department of Ophthalmology, Academic Medical Centre, Amsterdam, The Netherlands; University of Tasmania, AUSTRALIA

## Abstract

**Background:**

The retinal pigment epithelium (RPE) is a neural monolayer lining the back of the eye.

Degeneration of the RPE leads to severe vision loss in, so far incurable, diseases such as age-related macular degeneration and some forms of retinitis pigmentosa. A promising future replacement therapy may be autologous iris epithelial cell transdifferentiation into RPE *in vitro* and, subsequently, transplantation. In this study we compared the gene expression profiles of the iris epithelium (IE) and the RPE.

**Methods:**

We collected both primary RPE- and IE cells from 5 freshly frozen human donor eyes, using respectively laser dissection microscopy and excision. We performed whole-genome expression profiling using 44k Agilent human microarrays. We investigated the gene expression profiles on both gene and functional network level, using R and the knowledge database Ingenuity.

**Results:**

The major molecular pathways related to the RPE and IE were quite similar and yielded basic neuro-epithelial cell functions. Nonetheless, we also found major specific differences: For example, genes and molecular pathways, related to the visual cycle and retinol biosynthesis are significantly higher expressed in the RPE than in the IE. Interestingly, Wnt and aryl hydrocarbon receptor (AhR-) signaling pathways are much higher expressed in the IE than in the RPE, suggesting, respectively, a possible pluripotent and high detoxification state of the IE.

**Conclusions:**

This study provides a valuation of the similarities and differences between the expression profiles of the RPE and IE. Our data combined with that of the literature, represent a most comprehensive perspective on transcriptional variation, which may support future research in the development of therapeutic transplantation of IE.

## Introduction

In the vertebrate eye, the RPE is a monolayer of neural-crest derived cells located between the photoreceptors and the choroid. Dysfunctional RPE is involved in many retinal degenerative diseases such as age-related macular degeneration (AMD), Stargardt’s disease, Best’s disease and retinitis pigmentosa. For these disorders there is no (effective) treatment. One of the most promising future therapy options for RPE related disorders is cell replacement of the dysfunctional RPE.

Autologous intra-ocular RPE transplantation was previously carried out with limited success [1,2], since surgical variability and complications remained high. Therefore, many studies in the last decade focused on the development and use of induced pluripotent stem cells (iPSC) as a source for autologous cell replacement therapy. These iPSC can be differentiated *in vitro* towards RPE cells and used for experimental transplantation studies in animal models [3–5]. Recently, clinical stem cell/RPE replacement trials in patients with macula degeneration and patients with Stargardt’s disease were started [6,7].

Alternative strategies for retinal cell replacement are currently also being explored [8]. One of them involves *transdifferentiation*, also called *direct conversion*, the process of transforming an adult somatic cell into another adult somatic cell. With the acquired knowledge on differentiation of pluripotent stem cells towards RPE, the field of transdifferentiation has gained renewed interest. Humans have a limited capacity to transdifferentiate cells *in vivo* or spontaneously regenerate and restore their tissues and organs [9,10]. However, several studies demonstrated that *in vitro* procedures could convert one cell into another cell type and thereby skipping the pluripotent state, using overexpression of cell-lineage specific genes [11–15]. Recent studies also presented new strategies, using criteria such as common cellular origin and developmental plasticity, to identify “the best possible” cell for transdifferentiation [16,17].

In the literature, iris epithelium (IE) cells have been considered as potential starting source for transdifferentiation into the RPE and cell replacement therapy for several reasons [1,8,18–20]. First of all, both RPE and IE are neuro-epithelia with a common embryological origin (neuroectoderm of the developing optic cup). Next, IE cells can be obtained relatively easily through iridectomy in patients. Therefore, IE cells are a potentially autologous cell source, reducing the chance of transplant rejection. Finally, the IE seems suitable because *in vitro* cultured IE cells display a number of functional RPE features, such as the presence of tight junctions and the phagocytosis of photoreceptor outer segments [21,22].

To improve our understanding of molecular and functional similarities and differences between the human IE and RPE, we conducted a new, in depth microarray study, comparing gene expression profiles and the functional annotations of these two tissues *in vivo*.

## Results

### Similarities between the IE and the RPE transcriptomes

Following our previously published analyses strategies [23–25], we selected those genes with expression in the highest 10^th^ percentile for the RPE and the IE, assuming these genes to have the highest biological relevance. Using these files, the knowledge database Ingenuity attributed similar statistically significant biological functions, canonical pathways, and molecular networks to the RPE and the IE.

**Fig 1:**
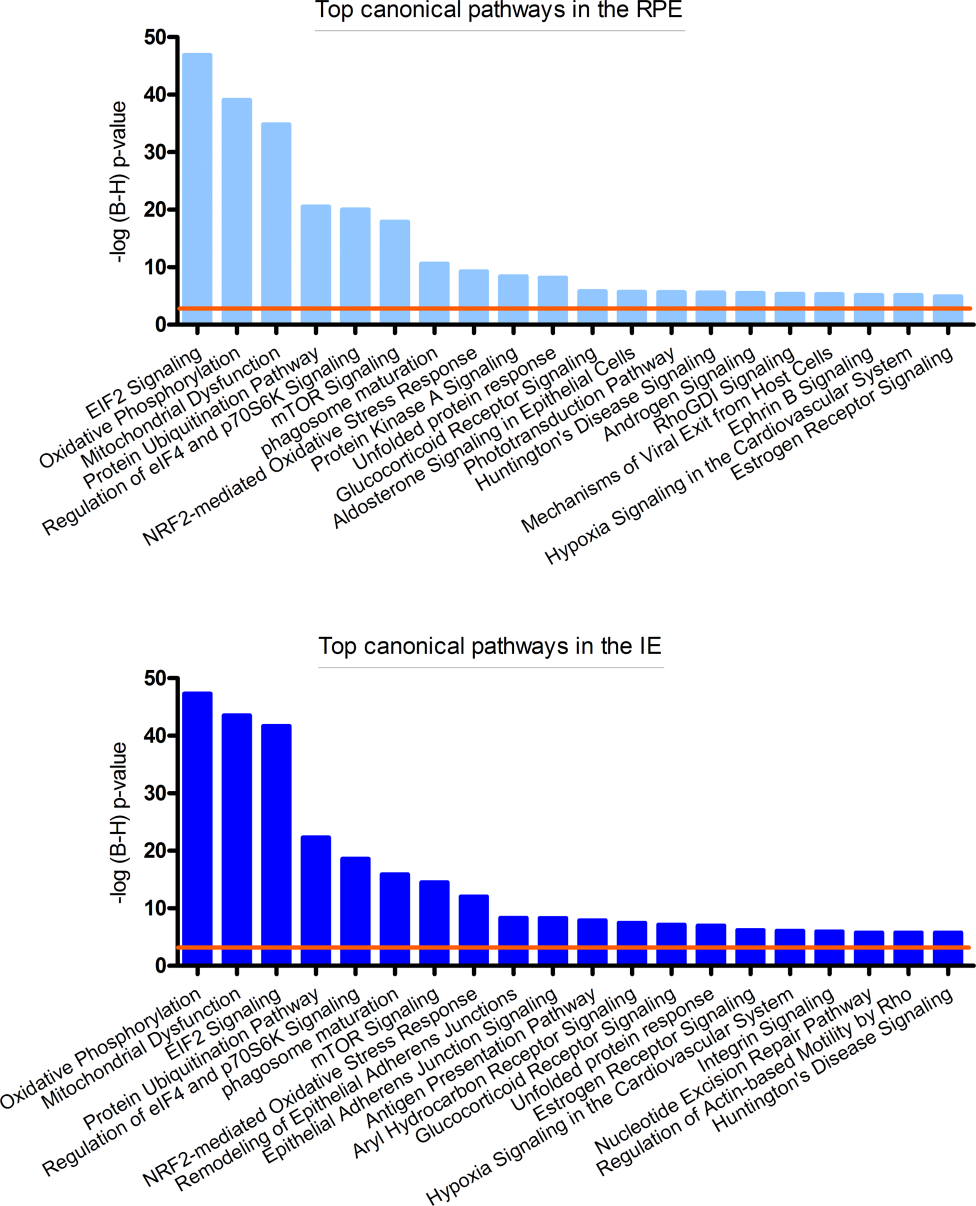
Top 20 significant canonical pathways of the core analysis in IPA (Ingenuity) of most highly expressed genes of the IE and the RPE. P-values indicate the significance of enrichment for the most highly expressed genes from our dataset. P-values were corrected for multiple testing using the Benjamini-Hochberg (B-H) false discovery rate. The upper graph (light blue bars) represents the–log(B-H) p-value of the RPE and the lower graph (dark blue bars) represents the–log(B-H) p-value of the IE. The orange line indicates the threshold of B-H corrected p<0.001.

The canonical pathways attributed to the highest percentile of the IE and RPE are quite similar (82.6% of these canonical pathways overlap). Many pathways underlie normal cellular physiology, which are similarly expressed in both cell types. Both the RPE and the IE show epithelial related canonical pathways such as Remodeling of Epithelial Adherens Junctions, Epithelial Adherens Junction Signaling, Integrin Signaling and Aldosterine Signaling in Epithelial Cells. The top 20 of these pathways are shown in Fig 1. Biological functions and molecular networks yielded similar functional annotations and can be found in the supplementary files (S1 Table).

We found considerable overlap between the canonical pathways expressed in the IE and the RPE. However, we observed significant differences as well. Here, we mainly focus on these differences.

### Differences between the IE and the RPE transcriptomes

To focus on the most differentially expressed genes between the two epithelia, we used stringent selection criteria (B-H adjusted p<0.001 and FC>5). We report a set of 700 unique genes (3.6%) significantly more expressed in the RPE than in the IE. Vice versa, 488 (2.5%) genes were significantly higher expressed in the IE compared to the RPE. Tables 1 and 2 show the top 30 of these genes. For the complete lists of statistically differentially expressed genes see S2 Table.

**Table 1:** Top 30 genes significantly more highly expressed in the RPE compared to the IE. The genes that are in bold were shown to be enriched in the human RPE [26]. Asterisks mark the genes that might be present in our dataset by contamination of the mRNA on the photoreceptor-RPE interface or may be expressed to some extent in both adjacent cell layers (also see Materials and methods).

RPE			
GeneName	SystematicName	Adj p value	FC
**RPE65**	**NM_000329**	**6.9E-05**	**167.7**
PCAT4	AK056825	1.2E-04	146.1
**SLCO1C1**	**NM_017435**	**3.5E-05**	**138.1**
GNGT1	NM_021955	5.9E-04	136.3
CNGA1*	NM_000087	5.4E-06	131.2
GUCA1B*	NM_002098	8.1E-06	116.1
**KIRREL2**	**NM_199180**	**7.5E-06**	**108.3**
MIR124-2HG	AK124256	6.1E-06	106.3
MAK	NM_005906	5.4E-06	98.5
LINC00982	AL833006	1.7E-05	96.8
**OPCML**	**NM_001012393**	**7.5E-06**	**94.8**
NEUROD1	NM_002500	7.8E-06	94.4
MPP4*	NM_033066	7.5E-06	93.8
**BEST1**	**NM_004183**	**4.3E-05**	**92.9**
**ITGB8**	**NM_002214**	**4.7E-04**	**91.1**
**DUSP6**	**NM_001946**	**1.3E-04**	**89.5**
**LRAT**	**NM_004744**	**4.0E-05**	**81.9**
NRL*	NM_006177	2.4E-05	81.5
RRH	NM_006583	1.9E-05	78.3
RP1*	NM_006269	6.1E-06	76.5
SAG*	NM_000541	8.5E-05	75.9
COL8A1	AL359062	2.5E-05	72.9
PDE6H	NM_006205	2.0E-05	72.4
AIPL1*	NM_014336	2.0E-05	70.5
KIAA1189	NM_020711	1.7E-05	68.0
LOC100507521	BX101632	1.6E-05	67.6
SLC26A7	NM_052832	8.0E-05	66.2
TMEM16B	NM_020373	7.5E-06	65.5
GNAT2	ENST00000351050	4.2E-05	60.8
SERINC4	ENST00000319327	7.5E-06	60.3

**Table 2:** Top 30 genes significantly more highly expressed in the IE compared to the RPE.

IE			
GeneName	SystematicName	Adj p value	FC
DCT	ENST00000377028	1.4E-09	128.5
WNT16	NM_057168	5.8E-10	125.9
GLULD1	NM_016571	2.2E-06	117.5
PDE1A	NM_001003683	5.2E-09	104.4
KLF5	NM_001730	7.3E-07	86.2
AGXT2L1	NM_031279	3.4E-07	78.6
ZIC1	NM_003412	5.6E-07	71.2
CLCA2	NM_006536	6.0E-08	68.8
C8orf47	NM_173549	2.1E-09	61.5
SRD5A2	NM_000348	9.1E-09	60.5
GJB2	NM_004004	3.1E-08	57.5
MYOC	NM_000261	5.2E-07	55.5
CPAMD8	NM_015692	2.5E-08	52.7
GJA3	NM_021954	3.6E-08	52.4
CRYGS	NM_017541	9.2E-08	51.8
SFRP2	NM_003013	1.8E-09	49.9
F5	NM_000130	7.5E-08	40.6
FBP2	NM_003837	4.8E-08	39.5
SNCAIP	NM_005460	7.8E-10	37.6
NPFFR2	NM_053036	3.3E-07	37.4
OTX1	NM_014562	2.3E-08	36.4
WNT5A	NM_003392	2.1E-08	33.5
TFEC	NM_012252	3.4E-08	33.0
DSC1	NM_004948	3.1E-08	32.8
LINC00403	AK055145	7.1E-06	32.7
NTF3	NM_002527	8.5E-07	30.7
GBP7	NM_207398	7.7E-09	29.7
CRYBB2P1	BC047380	3.3E-06	29.3
INDO	NM_002164	1.1E-06	26.9
ADAMTS16	NM_139056	2.9E-09	26.9

### Functional annotation of the genes that are enriched in the RPE

**Fig 2:**
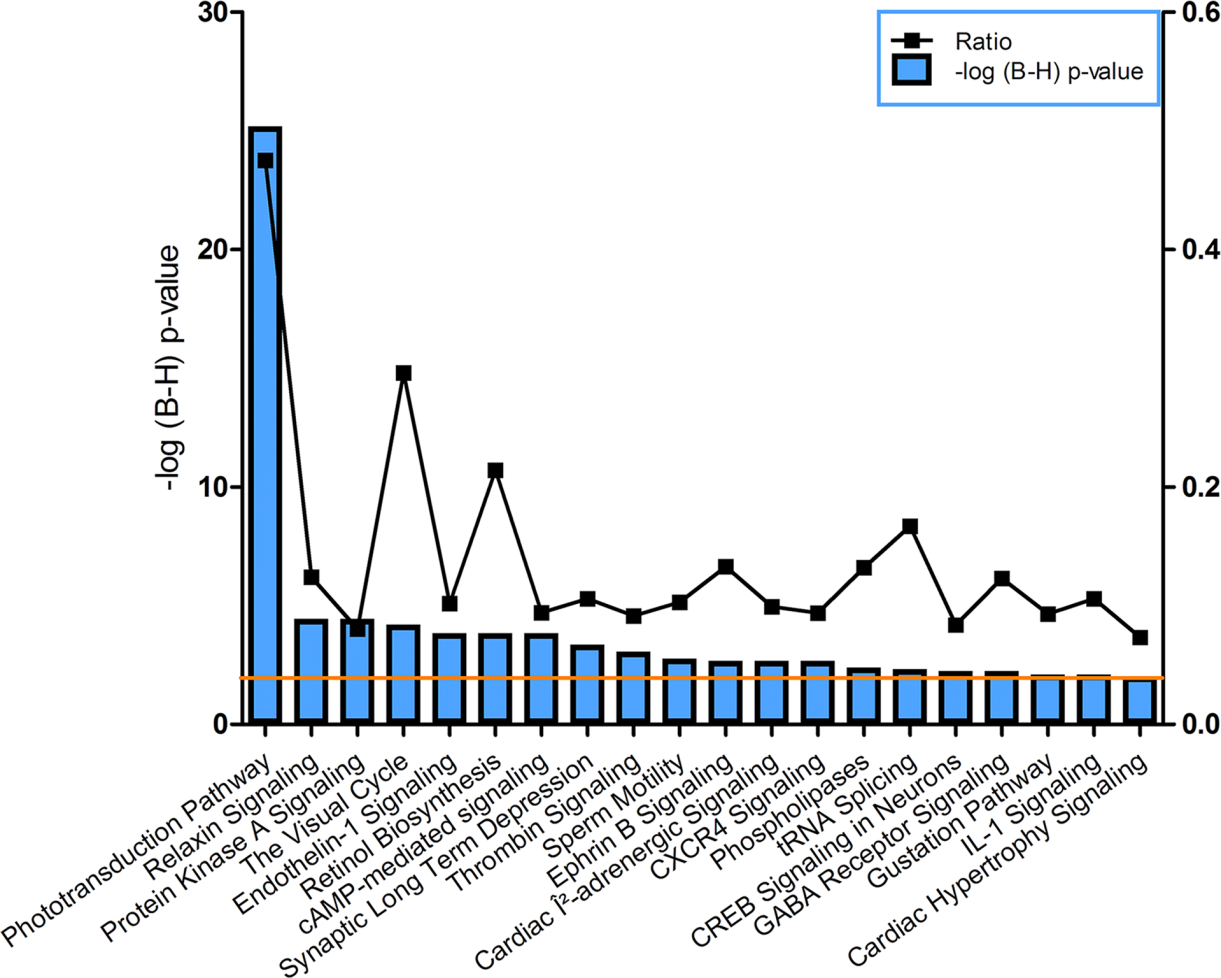
Canonical pathways identified by Ingenuity for the RPE enriched genes. The left y-axis displays the–log of the Benjamini-Hochberg corrected p-value. The right y-axis displays the ratio of number of genes derived from our dataset, divided by the total number of genes in the pathway. The bar graph represents the -log(B-H) p-value. The orange line indicates the threshold at a Benjamini-Hochberg corrected p<0.01.

Functional annotation of the genes that are enriched in the RPE (significantly more expressed in the RPE compared to the IE) yielded 28 canonical pathways (Fig 2). Interestingly, at least 4 of these pathways are directly related to the expression of the visual cascade: Phototransduction Pathway, The Visual Cycle, Retinol Biosynthesis, Retinoate Biosynthesis. Examples of genes that these different pathways have in common are *LRAT*, *RGR*, *RBP1*, *RDH5*, *RDH8*, *RDH10*, *RDH11*, *RDH12*, *RPE65*. For the complete list of the involved genes see S3 Table.

### Functional annotation of the genes that are enriched in the IE

**Fig 3:**
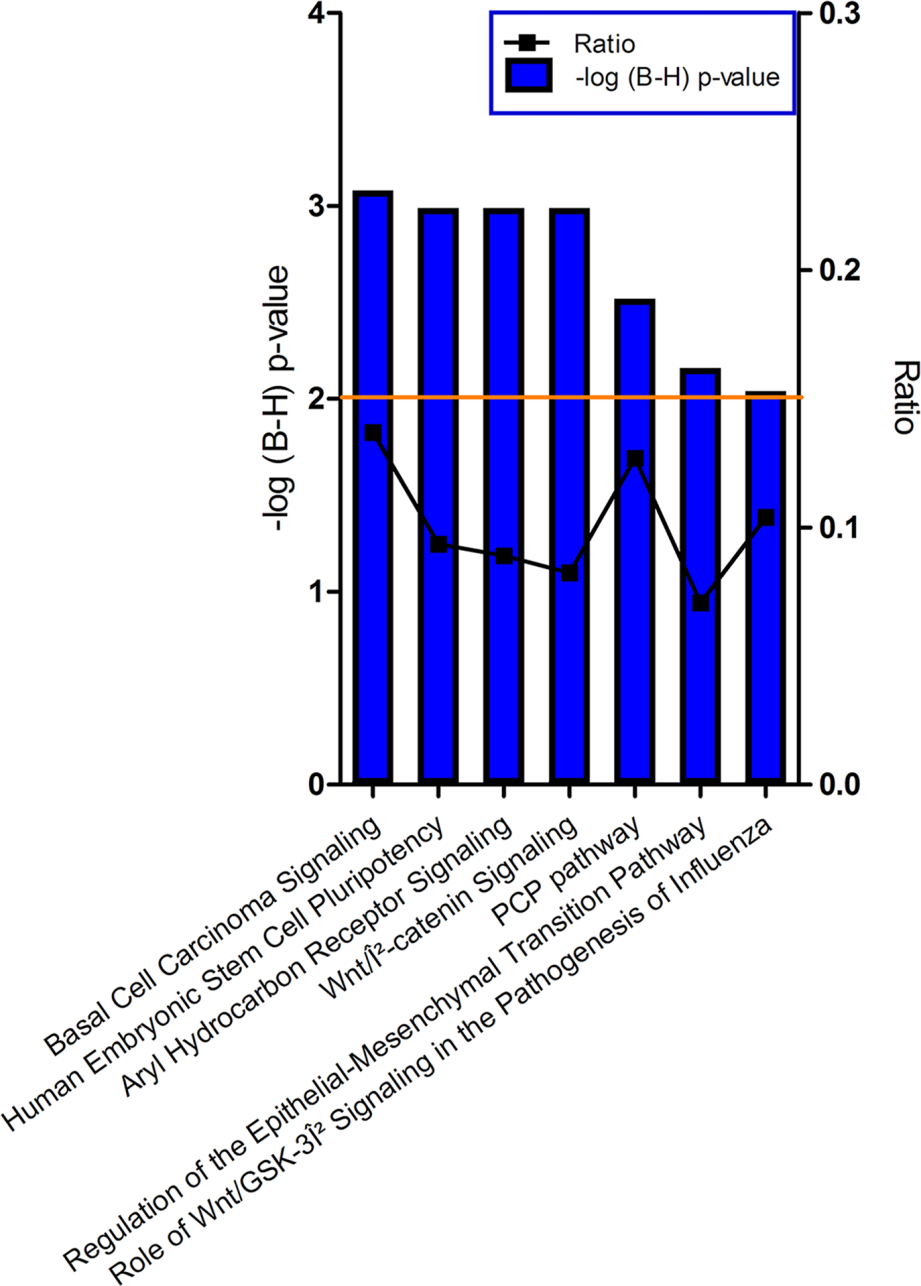
Canonical pathways identified by Ingenuity for the IE enriched genes. The left y-axis displays the–log of the Benjamini-Hochberg corrected p-value. The right y-axis displays the ratio of number of genes derived from our dataset, divided by the total number of genes in the pathway. The bar graph represents the -log(B-H) p-value. The orange line indicates the threshold at a Benjamini-Hochberg corrected p<0.01.

Ingenuity assigned several canonical pathways to the genes in the IE specific dataset (significantly more expressed in the IE compared to the RPE) (Fig 3). Four of the five significant canonical pathways: Basal Cell Carcinoma Signaling, Human Embryonic Stem Cell Pluripotency, Wnt/B-catenin Signaling, and PCP Pathway have large overlap in participating genes. These include multiple *WNT* genes (*4*, *16*, *10A*, *2B*, *5A*, *7A*, *7B*), *FZD10*, *NTF3*, *PDGFD*, *TGFB3*. *GLI3*, *GLIS1*, *PDGFD*, *ZIC3*, *INHBA*, *SOX1*, *CD44*, *SOX11*, *SFRP2*, *GJA1*.

The fifth pathway, Aryl Hydrocarbon Receptor Signaling is a more exclusive pathway, derived from the highly expressed *TGFB3*, *GSTA3*, *HSPB3*, *HSPB2*, *DCT*, *GSTM1*, *HSPB7*, *ALDH1L2*, *NR2F1*, *TYR*, *FAS*, *ALDH3A1* and *CYP1B1* genes. For the complete list of the involved genes see S4 Table.

### Genes associated with established RPE functions in the IE and RPE

**Fig 4:**
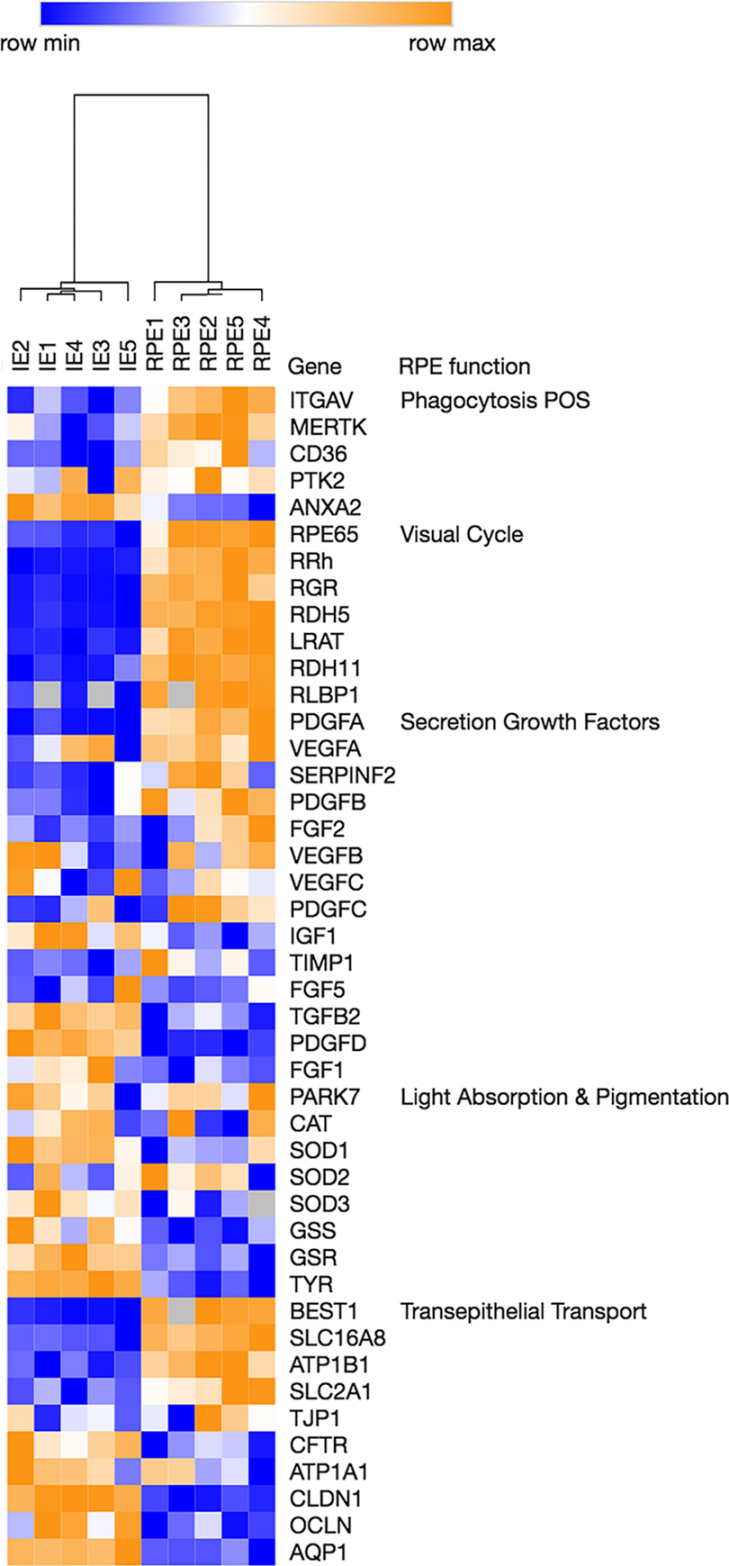
Heatmap for the expression of genes related to RPE specific functions. The normalized expression data are converted to heat map color using the mean and maximum values for each gene. The intensity scale of the standardized expression values ranges from dark blue (low expression) to dark orange (high expression). We added a hierarchical cluster tree that shows that the IE samples cluster together and the RPE samples cluster together.

To evaluate the expression of genes involved in well-known RPE functions in both the RPE and IE of the individual samples, we compiled a list of most important RPE functions. We determined the categories according to what we derived from our dataset described here, and found in the literature [27]: “phagocytosis of photoreceptor outer segments” [28–30], “visual cycle” [31], “secretion of factors and signaling molecules” [23], “light absorption and pigmentation” [32–35] and”transepithelial transport and pH regulation” [36–39]. Subsequently, we selected the genes known to be involved in these functions and investigated the corresponding normalized IE and RPE gene expression levels in all samples, resulting in a heat map for these entries (Fig 4).

Interestingly, we found a very clear and consistent distinction in normalized expression levels across all samples between the RPE and IE. Especially for the expression of “visual cycle” and “phagocytosis photoreceptor outer segments” we found a pronounced difference. Also the normalized expression of genes in “transepithelial transport” and “light absorption and pigmentation” are undoubtedly tissue specific. Only the “secretion of growth factors” has some heterogeneity, however a hierarchical clustering tree shows that, overall, there is low intra-epithelial variability.

In addition, we next composed a list of RPE expressed genes that were previously implicated in normal RPE function and/or retinal diseases [39], which we provide in the supplementary files for the interested reader (S1 Fig and S5 Table).

## Discussion

There are a vast number of studies about the (promising) use of (pluripotent) stem cell derived RPE cells for transplantation use in degenerative disorders of the RPE [40]. However, instead of stem cells, several authors suggested to use IE cells for RPE replacement. Thumann et al [41] and Abe et al [18] argued that the IE and RPE have a common neuro-ectodermal origin and that an IE biopsy can be relatively easily obtained from the patient by iridectomy.

To investigate this idea further, we compared the gene expression profiles of the human RPE and the IE *in vivo*. We aimed to gain more insight into the (molecular) differences and similarities between these tissues.

### Similarities between the IE and the RPE transcriptomes

The canonical pathways and corresponding statistically significantly enriched functions for the most highly expressed genes of the IE and the RPE were very similar.

However, there was also a set of statistically significantly differentially expressed genes. Out of 19596 unique genes, 700 (3.6%) were enriched in the RPE and 488 (2.5%) were enriched in the IE (S2 Table). It is important to note here that the cutoff values we chose in this study are relatively strict to make the study as comprehensible as possible. Obviously, more relaxed comparison parameters would yield more, but less significant differences between RPE and IE.

### RPE enriched gene expression compared to the IE

Prominent features among the enriched RPE gene expression are those implicated in the phototransduction cascade. Obviously, *in vivo*, the expression of these genes is most likely invoked by the activation of rhodopsin and, subsequently, the entire phototransduction cascade in the adjacent photoreceptors.

An alternative explanation is the presence of some degree of photoreceptor contamination in our RPE samples, which is unavoidable even when we use meticulous laser dissection technology. This may have caused the enrichment of the phototransduction cascade in the RPE compared to the IE.

### IE enriched gene expression compared to the RPE

#### Wnt signaling pathway is active in the IE, but not in the RPE

Ingenuity attributed specific canonical pathways to the IE that are related to the Wnt signaling pathway (Wnt SP): Basal Cell Carcinoma Signaling, Human Embryonic Stem Cell Pluripotency, Wnt/B-catenin Signaling and PCP Pathway. In general, the Wnt signaling pathway consists of a group of signal transduction pathways that regulate crucial aspects of cell fate determination, cell proliferation, cell polarization, neural patterning and organogenesis during embryonic development [42].

In general, Wnt SP expression maintains pluripotency and self-renewal in mouse and human embryonic stem cells [43,44]. The activation of Wnt SP improves the efficiency of reprogramming of somatic cells, including retinal neurons, into iPSCs, both *in vitro* and *in vivo* [45–47]. This role is not only complex, but also time and dose dependent [48]. The Wnt SP is also crucial for the differentiation of pluripotent stem cells to RPE cells, but the impact on different stages of RPE differentiation from human embryonic stem cells is not yet well understood [49–51].

Nonetheless, the high expression of Wnt SP genes in the IE compared to the RPE suggests that the IE preserves (part of) its multipotent character during life. Indeed, a number of previous studies in chicken, rodents, pigs and humans also suggested the presence of multipotent neural progenitor cells in the IE [52–54]. The human IE can be cultured in neurosphere formation, displaying retinal stem/progenitor cell properties [19,55] (and own unpublished observation). Finally, transduction and expression of only a few genes (*CRX*, *RX* and *NEUROD*) induced a functional photoreceptor like phenotype from rodents, primates and human iris cells [55]. Taken together, the available data suggest that IE cells retain, at least to some degree, developmental or functional plasticity, which may proof beneficial for potential therapeutic strategies for RPE replacement.

#### The aryl hydrocarbon signaling pathway is active in the IE but not in the (aged) RPE

Our Ingenuity analysis showed a high expression of the aryl hydrocarbon receptor (AhR) signaling pathway in the IE compared to the RPE. AhR is a ligand dependent transcription factor that regulates a cellular defense mechanism pathway against toxin overload in cells. Toxin overload in RPE cells may come from daily rhythmic phagocytosis of photoreceptor outer segments, oxidative stress and damaging light exposure. Several functional studies previously showed that the detoxifying AhR pathway is also active in human RPE, but that this activity decreases with age [56,57]. Indeed, our samples are derived from older donors, which might explain the relatively low expression of genes involved in AhR signaling in RPE samples. Interestingly, Esfandiary [58] et al found an association between detoxification genes, including AhR, and AMD. These data were supported by studies on a AhR-/- mice which presented features of AMD pathogenesis, including thick focal and diffuse sub-RPE deposits, regions of retinal hyper- and hypopigmentation as well as RPE degeneration [57,59,60].

Combining our data with those of the literature, older IE cells and relatively young RPE cells show high AhR related expression and detoxification functionalities, whereas older RPE cells do not. It is tempting to speculate here that older IE cells maintain specific detoxification capacities during life, whereas the corresponding RPE does not.

### Well-known RPE functions

For further insight in the presence or absence of potential RPE functionalities in the IE we analyzed a number of well-known RPE specific functions.

For a limited number of RPE functions (“visual cycle”, “phagocytosis of photoreceptor outer segments”, “transepithelial transport” and “light absorption and pigmentation”) the underlying genes follow a unique and characteristic differential expression pattern in the RPE or in the IE across all donor eyes (Fig 4). One might thus argue that these functionalities are not present in the IE cells and may (have to) be invoked upon transformation of IE cell to RPE cell. Previous studies have shown that both human IE and RPE cells can be maintained and expanded *in vitro* [61–63], and are then able to phagocytize photoreceptor outer segments when provided in the medium [64]. In addition, cultured autologous IE cells were previously transplanted in monkeys, and they were able to phagocytize photoreceptor outer segments even 6 months after transplantation [65]. This support the flexibility of the IE cells to take on RPE functions, depending on microenvironmental factors.

For other RPE functions (secretion of growth factors, light absorption and pigmentation) gene expression is more heterogeneous across the IE and RPE samples, without disturbing the normal functions of the tissues in these individuals. Thus these functions may either be not fully specific or redundant in the IE or RPE.

### Our IE-RPE microarray results compared to the literature

To our knowledge, only one other microarray study in the literature addressed potential gene expression similarities and differences between human IE and RPE: Cai and coworkers [66] concluded that there are major differences in gene expression profiles of IE and RPE, including lack of expression in IE of genes known to be critical for RPE function. Also they concluded that the native IE gene expression profile and corresponding functionalities may be a potential obstacle for successful subretinal transplantation. In our current study, we explored IE and RPE gene expression in a much larger dataset (we measured more than seven times the amount of gene probes), and we included extensive bioinformatics as well as functional annotation. For detailed technical and statistical differences between the study of Cai et al and our study, see S2 Fig. Our data and analysis partly support and extend the conclusions of Cai and coworkers.

Besides large similarities, we also find major differences in gene expression profiles between IE and RPE. We estimate these differences to affect at least 6.1% of the transcriptome. This appears not be a large difference, if we consider the findings of Van Soest et al [67], who concluded that the transcriptome differences between macular and peripheral RPE were 2–3%. Nonetheless, such difference in transcriptomes and related functionalities may be an obstacle for direct transplantation.

A number of new findings and considerations from our study may be of interest:

1. The IE and RPE show many similarities based on their gene expression profiles.
2. The aryl hydrocarbon signaling pathway is active in the IE and young RPE, but not in the (aged) RPE [57]. This may represent a difference in detoxification capacity between the two tissues.
3. The high activity of Wnt SP may reflect the multipotent character of IE cells. This could be of interest to studies that will further investigate IE’s therapeutic potential.

## Summary and conclusions

In conclusion, our study provides in depth analysis of the gene expression profiles of the IE and the RPE. We analyzed these profiles to determine and report the differences and similarities between the two related tissues. Our data may be useful in the further exploration of IE as a potential source for regenerative medicine for RPE degeneration.

## Materials & methods

### Ethics statement

This study was performed in agreement with the declaration of Helsinki on the use of human material for research. The human donor eyes were obtained from the Netherlands Brain Bank (NBB) (Amsterdam, The Netherlands). The NBB obtained permission (informed consent) from the donors for enucleation of the eyes and to use the eyes for scientific purposes. All procedures of the NBB have been approved by the ethics Committee of VU University Medical Center (Amsterdam, The Netherlands) under the reference number 2009/148. All data were analyzed anonymously.

### Tissue collecting and processing

We selected 5 donor eyes (3 male, 2 female). Donors were aged 49 to 73 at time of death. Donors were selected for not having any ophthalmic disorder or malignancy, ocular abnormalities on visual or histological inspection, drusen and poor morphology [23]. Globes were enucleated and snap-frozen between 10 and 22 hours post mortem. The eyes were stored at -80C until use. For full details see S6 Table. From each donor eye we collected both the IE and the RPE in order to reduce genetic variation in our study design.

To collect the RPE, a macular fragment of 16mm^2^ with the fovea in its center was cut from the retina.12uM Sections from the macular area were used to isolate the RPE cells [67]. The sections were dehydrated with ethanol and air-dried before micro dissection. To minimize cellular cross-contamination in our procedure, we used the meticulous laser dissection microscope to cut the RPE monolayer specifically (PALM Carl Zeiss, MicroImaging GmbH, Munich, Germany). Nonetheless, considering the proximity and interactivity of the photoreceptors and the RPE, the chance of some contamination of adjacent cell layers is very high. This has been previously observed and extensively discussed elsewhere [23,67].

To collect the IE, the anterior part of the eye was excised at the level of the ora serrata. This anterior part was snap frozen in isopentane in liquid nitrogen and stored at -80C. We removed the ciliary body from the anterior part to expose the iris. While keeping the eye frozen we scraped and collected the iris epithelium with forceps, detaching it from the stroma.

When we collected and select our samples, specificity of the tissue and integrity of the RNA are most important to ensure valid results. We used different techniques to collect the IE and the RPE, which is necessary for the specificity of the tissues.

To ensure that our findings are a reflection of a clear difference between IE and RPE and that the variance within sample groups is less than between, we conducted a principal component analysis (S3 Fig). The first component separates the IE samples from the RPE samples and explains 89% of the total variance in the data. In addition we made an overview of the measured expression levels of possible photoreceptor contaminating genes (S7 Table and S4 Fig).

RNA isolation, amplification and labelling procedures were carried out essentially as described elsewhere [24]. High quality RNA is challenging with postmortem ocular tissues, compared to isolating RNA from fresh cell cultures. Postmortem changes of the RNA can be determined by measuring its integrity. Given the lengthy procedures of sample selection, procedure and extensively quality controls, we included a limited number of the “very best samples” in our microarray analysis. To clarify: If RNA integrity was compromised in either the IE or RPE, no samples of this donor eye were used. We always used both IE and RPE from the same eye to minimize the variance. Quality of the total RNA was checked with a Bioanalyzer assay (RNA 6000 Pico Kit, Agilent Technologies, Amstelveen, The Netherlands). The RIN values of the tRNA ranged from 5.1 to 9 and the peak of the fragment length of the aRNA samples varied between 700 and 900nt (S5 Fig).

In our microarray study we used a common reference design. As a common reference we used RNA from human RPE/choroid that was used in previous and on-going gene expression analyses in our lab [24,25,68]. The common reference was prepared from human RPE/choroid RNA that was isolated, amplified using the same methodology as our experimental samples, and labelled with Cy3 (Cy3 mono-reactive dye pack, GE Healthcare UK, Little Chalfont, Buckinghamshire, UK). See Janssen et al [24] for a more detailed description of the laser dissection procedures, RNA processing and microarray procedures.

### Microarray data analysis

The microarray data were extracted using Agilent Feature Extraction Software (Agilent Technologies, version 9.5.3.1), see S6 Fig. Raw data were imported into R (version 2.14.0 for Windows, R Development Core Team, 2009) using the Bioconductor package LIMMA. Background correction was performed using the “normexp” method with an offset of 10 to adjust the foreground signal without introducing negative values. The resulting log-ratios were transformed using intensity-dependent loess normalization. We further normalized the average intensities across arrays using the aquantile method [69]. The microarray data is available in the Gene Expression Omnibus database with the accession number GSE81058. We ranked the normalized intensities in the Cy5 channel corresponding to the experimental samples. Based on these ranks we divided the normalized intensities in bins corresponding to the highest 10 percentile, the 50th– 90^th^ percentile, 10^th^-50^th^ percentile and lowest 10^th^ percentile.

Genes that are differentially expressed between the RPE and the IE were identified on the normalized log-ratios using a linear model with patient as blocking factor. Significant differences were determined using Bayes moderated paired t-statistics (package LIMMA). Resulting p-values were corrected for multiple testing using the Benjamini-Hochberg False Discovery Rate adjustment. We used stringent statistical analysis and the paired t test to determine those IE-RPE differences that overcome the variation between the individual donors. To identify explicit differences between the IE and the RPE we used cutoff values of a fold change (FC) >5 and a p-value<0.001. We selected these stringent cutoff values because with the initial selection criteria of FC>2.5 and a p-value<0.001 we found 1277 genes enriched in the IE and 1581 genes in the RPE and we wanted to analyse the most significant differences between the IE and RPE, instead of less significant differences that are probably based on overlapping gene involvement in multiple functionalities. The genes derived from this analysis are referred to as “Significantly highly expressed in the IE” and “significantly highly expressed in the RPE”.

Functional annotation was done in IPA, Ingenuity (Ingenuity Systems, version 24718999, assessed at September 14^th^, 2015). To present the results as comprehensive as possible we highlighted the Ingenuity canonical pathways only because they depict the most simple and straightforward representation of our data and functionalities. The associated biological functions and diseases are described in the supplementary files (S1 and S2 Tables). To visualize the normalized expression data for the RPE specific functions we used the GENE-E software [70]. We made use of the hierarchical clustering function, using a Pearson correlation metric, to visualize the variation within our sample sets.

### Confirmation of microarray results

**Fig 5:**
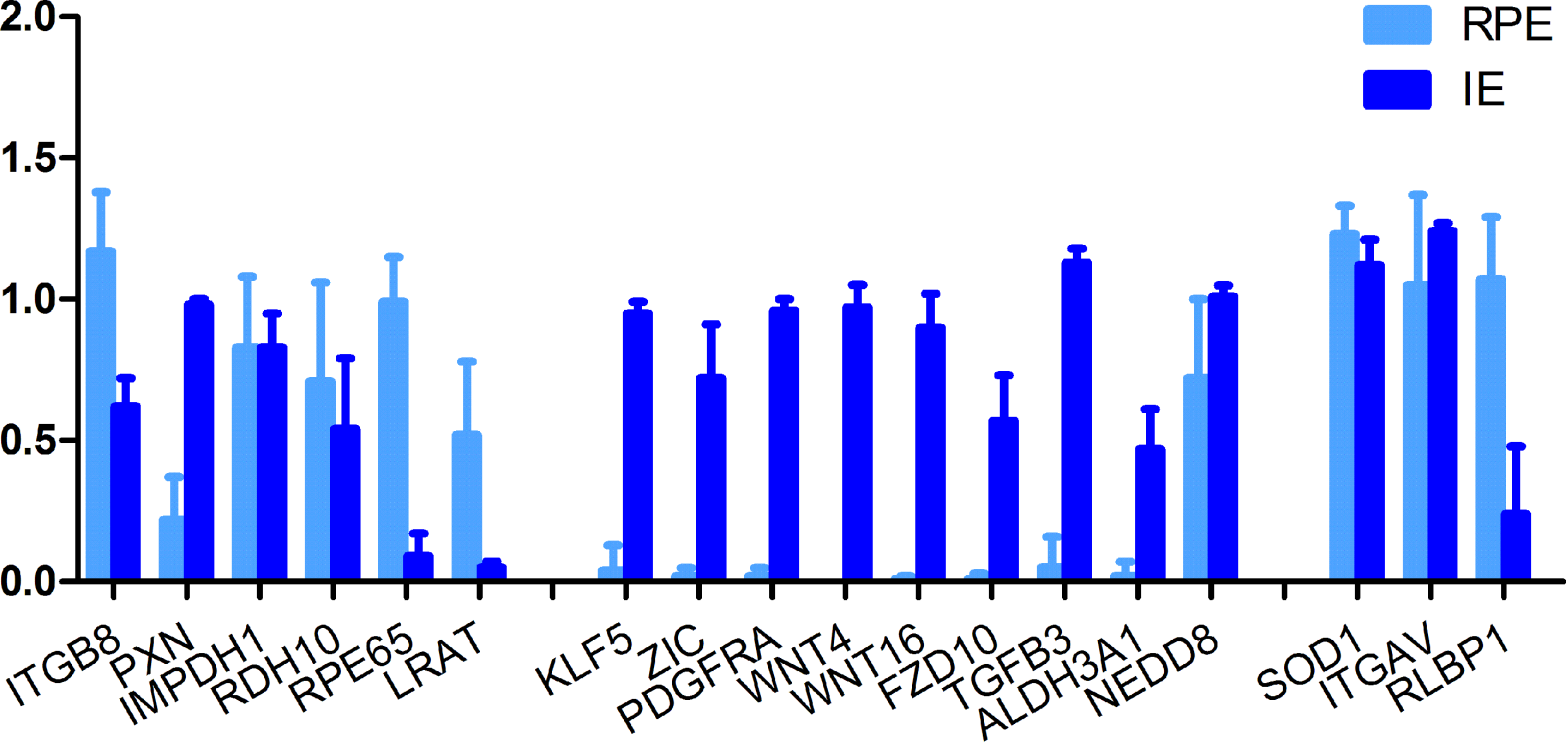
Confirmation of microarray results by sqRT-PCR. We used GAPDH as the housekeeping gene to normalize the gene expression of the IE and RPE samples. We depict the median and standard deviation for RPE samples in light blue, IE samples in dark blue. We selected genes that were highly expressed in the RPE (*ITGB8*, *PXN*, *IMPDH1*, *RDH10*, *RPE65*, *LRAT*), highly in the IE (*KLF5*, *ZIC*, *PDGFRA*, *WNT4*, *WNT16*, *FZD10*, *TGFB3*, *ALDH3A1*, *NEDD8*) and in both groups (*SOD1*, *ITGAV*, *RLBP1*). We find 89% (16 of the 18 genes) to be in agreement with the microarray results, only *PXN* and *RLBP1* give a different result.

We confirmed our microarray data with sqRT-PCR, see Fig 5 and S7 Fig for the photos of the gel electrophoresis. For a detailed description of the sqRT-PCR, see Janssen et al [24]. In short, sqRT-PCR was carried out using intron-spanning primers on cDNA from IE and RPE, using up to 5 biological replicates. To minimize effects of RNA degradation artefacts in the human post mortem samples, we generated primers near the 3’end of the gene. We quantified the gene expression in ImageJ and normalized expression by comparing it to the measured expression of housekeeping gene GAPDH.

## Supporting information

S1 Fig Heatmap for the expression of genes involved in disorders of the RPE. (TIF)

S2 Fig Technical and statistical differences between the study of Cai et al and our study. (TIF)

S3 Fig Principal component analysis of the RPE and IE samples. (TIFF)

S4 Fig Mean expression and standard deviation of the measured expression of photoreceptor genes in the RPE and IE samples. (TIFF)

S5 Fig Overview of the RNA quality control for our samples. (TIF)

S6 Fig Quality control reports of the microarrays. (PDF)

S7 Fig Gel images of the RT-PCR experiment to validate the microarray. (PDF)

S1 Table Biological functions and molecular networks of the highest 10 percentile of the RPE and IE. (XLS)

S2 Table Significantly differentially expressed genes with FC5. (XLS)

S3 Table Canonical pathways of RPE enriched genes. (XLS)

S4 Table Canonical pathways of IE enriched genes. (XLSX)

S5 Table Expression of genes in the IE and RPE that are involved in genetic retinal diseases originating in the RPE. (XLSX)

S6 Table Background information donor eyes. (XLS)

S7 Table Mean expression and standard deviation of the measured expression of photoreceptor genes. (XLS)

## References

[1] Sheridan CM, Mason S, Pattwell DM, Kent D, Grierson I, Williams R. Replacement of the RPE monolayer. Eye. 2009;23: 1910–1915. doi: 10.1038/eye.2008.420 19169229 10.1038/eye.2008.420

[2] van Meurs JC (last), Kirchhof B, MacLaren R, in Schachat AP, Wilkinson CP, Hinton DR, et al. Ryan’s Retina 6th edition, Chapter 124: Retinal Pigment Epithelium and Choroid Translocation in Patients with Age-Related Macular Degeneration. Mosby; 2016.

[3] Li Y, Tsai Y-T, Hsu C-W, Erol D, Yang J, Wu W-H, et al Long-term safety and efficacy of human-induced pluripotent stem cell (iPS) grafts in a preclinical model of retinitis pigmentosa. Mol Med. 2012;18: 1312–1319. doi: 10.2119/molmed.2012.00242 22895806 10.2119/molmed.2012.00242 PMC3521789

[4] Maeda T, Lee MJ, Palczewska G, Marsili S, Tesar PJ, Palczewski K, et al Retinal pigmented epithelial cells obtained from human induced pluripotent stem cells possess functional visual cycle enzymes in vitro and in vivo. J Biol Chem. 2013;288: 34484–34493. doi: 10.1074/jbc.M113.518571 24129572 10.1074/jbc.M113.518571 PMC3843063

[5] Stanzel BV, Liu Z, Somboonthanakij S, Wongsawad W, Brinken R, Eter N, et al Human RPE stem cells grown into polarized RPE monolayers on a polyester matrix are maintained after grafting into rabbit subretinal space. Stem Cell Rep. 2014;2: 64–77. doi: 10.1016/j.stemcr.2013.11.005 24511471 10.1016/j.stemcr.2013.11.005 PMC3916756

[6] Schwartz SD, Tan G, Hosseini H, Nagiel A. Subretinal Transplantation of Embryonic Stem Cell-Derived Retinal Pigment Epithelium for the Treatment of Macular Degeneration: An Assessment at 4 Years. Invest Ophthalmol Vis Sci. 2016;57: ORSFc1–9. doi: 10.1167/iovs.15-18681 27116660 10.1167/iovs.15-18681

[7] Wiley LA, Burnight ER, DeLuca AP, Anfinson KR, Cranston CM, Kaalberg EE, et al cGMP production of patient-specific iPSCs and photoreceptor precursor cells to treat retinal degenerative blindness. Sci Rep. 2016;6: srep30742 doi: 10.1038/srep30742 27471043 10.1038/srep30742 PMC4965859

[8] Dhamodaran K, Subramani M, Ponnalagu M, Shetty R, Das D. Ocular stem cells: a status update! Stem Cell Res Ther. 2014;5: 56 doi: 10.1186/scrt445 25158127 10.1186/scrt445 PMC4055087

[9] Singer AJ, Clark RAF. Cutaneous Wound Healing. N Engl J Med. 1999;341: 738–746. doi: 10.1056/NEJM199909023411006 10471461 10.1056/NEJM199909023411006

[10] Michalopoulos GK. Liver Regeneration. J Cell Physiol. 2007;213: 286–300. doi: 10.1002/jcp.21172 17559071 10.1002/jcp.21172 PMC2701258

[11] Vierbuchen T. Direct conversion of fibroblasts to functional neurons by defined factors. Nature. 2010;463: 1035–1041. doi: 10.1038/nature08797 20107439 10.1038/nature08797 PMC2829121

[12] Yoo AS. MicroRNA-mediated conversion of human fibroblasts to neurons. Nature. 2011;476: 228–231. doi: 10.1038/nature10323 21753754 10.1038/nature10323 PMC3348862

[13] Caiazzo M. Direct generation of functional dopaminergic neurons from mouse and human fibroblasts. Nature. 2011;476: 224–227. doi: 10.1038/nature10284 21725324 10.1038/nature10284

[14] Guo Z. In vivo direct reprogramming of reactive glial cells into functional neurons after brain injury and in an Alzheimer’s disease model. Cell Stem Cell. 2014;14: 188–202. doi: 10.1016/j.stem.2013.12.001 24360883 10.1016/j.stem.2013.12.001 PMC3967760

[15] Su Z, Niu W, Liu ML, Zou Y, Zhang CL. In vivo conversion of astrocytes to neurons in the injured adult spinal cord. Nat Commun. 2014;5: 3338 doi: 10.1038/ncomms4338 24569435 10.1038/ncomms4338 PMC3966078

[16] Lanza R, Atala A. Essentials of Stem Cell Biology. Academic Press; 2013.

[17] Heinrich C, Spagnoli FM, Berninger B. In vivo reprogramming for tissue repair. Nat Cell Biol. 2015;17: 204–211. doi: 10.1038/ncb3108 25720960 10.1038/ncb3108

[18] Abe T, Yoshida M, Yoshioka Y, Wakusawa R, Tokita-Ishikawa Y, Seto H, et al Iris pigment epithelial cell transplantation for degenerative retinal diseases. Prog Retin Eye Res. 2007;26: 302–321. doi: 10.1016/j.preteyeres.2007.01.003 17324604 10.1016/j.preteyeres.2007.01.003

[19] Thumann G, Stöcker M, Maltusch C, Salz AK, Barth S, Walter P, et al High efficiency non-viral transfection of retinal and iris pigment epithelial cells with pigment epithelium-derived factor. Gene Ther. 2010;17: 181–189. doi: 10.1038/gt.2009.124 19741732 10.1038/gt.2009.124

[20] Jasty S, Srinivasan P, Pasricha G, Chatterjee N, Subramanian K. Gene expression profiles and retinal potential of stem/progenitor cells derived from human iris and ciliary pigment epithelium. Stem Cell Rev. 2012;8: 1163–1177. doi: 10.1007/s12015-012-9394-3 22744312 10.1007/s12015-012-9394-3

[21] Rezai KA, Lappas A, Kohen L, Wiedemann P, Heimann K. Comparison of tight junction permeability for albumin in iris pigment epithelium and retinal pigment epithelium in vitro. Graefes Arch Clin Exp Ophthalmol Albrecht Von Graefes Arch Für Klin Exp Ophthalmol. 1997;235: 48–55. 10.1007/BF01007837 9034842

[22] Thumann G. Development and Cellular Functions of the Iris Pigment Epithelium. Surv Ophthalmol. 2001;45: 345–354. doi: 10.1016/S0039-6257(00)00195-8 11166346 10.1016/s0039-6257(00)00195-8

[23] Booij JC, van Soest S, Swagemakers SMA, Essing AHW, Verkerk AJMH, van der Spek PJ, et al Functional annotation of the human retinal pigment epithelium transcriptome. BMC Genomics. 2009;10: 164 doi: 10.1186/1471-2164-10-164 19379482 10.1186/1471-2164-10-164 PMC2679759

[24] Janssen SF, Gorgels TGMF, Bossers K, Ten Brink JB, Essing AHW, Nagtegaal M, et al Gene expression and functional annotation of the human ciliary body epithelia. PloS One. 2012;7: e44973 doi: 10.1371/journal.pone.0044973 23028713 10.1371/journal.pone.0044973 PMC3445623

[25] Janssen SF, van der Spek SJF, Ten Brink JB, Essing AHW, Gorgels TGMF, van der Spek PJ, et al Gene expression and functional annotation of the human and mouse choroid plexus epithelium. PloS One. 2013;8: e83345 doi: 10.1371/journal.pone.0083345 24391755 10.1371/journal.pone.0083345 PMC3877019

[26] Bennis A, Gorgels TGMF, Brink JB ten, van der Spek PJ, Bossers K, Heine VM, et al Comparison of Mouse and Human Retinal Pigment Epithelium Gene Expression Profiles: Potential Implications for Age-Related Macular Degeneration. PLOS ONE. 2015;10: e0141597 doi: 10.1371/journal.pone.0141597 26517551 10.1371/journal.pone.0141597 PMC4627757

[27] Strauss O. The retinal pigment epithelium in visual function. Physiol Rev. 2005;85: 845–881. doi: 10.1152/physrev.00021.2004 15987797 10.1152/physrev.00021.2004

[28] Finnemann SC, Bonilha VL, Marmorstein AD, Rodriguez-Boulan E. Phagocytosis of rod outer segments by retinal pigment epithelial cells requires αvβ5 integrin for binding but not for internalization. Proc Natl Acad Sci U S A. 1997;94: 12932–12937. 9371778 10.1073/pnas.94.24.12932 PMC24241

[29] Finnemann SC, Nandrot EF. MERTK ACTIVATION DURING RPE PHAGOCYTOSIS IN VIVO REQUIRES αVβ5 INTEGRIN. Adv Exp Med Biol. 2006;572: 499–503. doi: 10.1007/0-387-32442-9_69 17249615 10.1007/0-387-32442-9_69 PMC3577060

[30] Law A-L, Ling Q, Hajjar KA, Futter CE, Greenwood J, Adamson P, et al Annexin A2 regulates phagocytosis of photoreceptor outer segments in the mouse retina. Mol Biol Cell. 2009;20: 3896–3904. doi: 10.1091/mbc.E08-12-1204 19587120 10.1091/mbc.E08-12-1204 PMC2735488

[31] Thompson DA, Gal A. Vitamin A metabolism in the retinal pigment epithelium: genes, mutations, and diseases. Prog Retin Eye Res. 2003;22: 683–703. doi: 10.1016/S1350-9462(03)00051-X 12892646 10.1016/s1350-9462(03)00051-x

[32] Bazan NG. Survival signaling in retinal pigment epithelial cells in response to oxidative stress: significance in retinal degenerations. Adv Exp Med Biol. 2006;572: 531–540. doi: 10.1007/0-387-32442-9_74 17249620 10.1007/0-387-32442-9_74

[33] Plafker SM, O’Mealey GB, Szweda LI. MECHANISMS FOR COUNTERING OXIDATIVE STRESS AND DAMAGE IN RETINAL PIGMENT EPITHELIUM. Int Rev Cell Mol Biol. 2012;298: 135–177. doi: 10.1016/B978-0-12-394309-5.00004-3 22878106 10.1016/B978-0-12-394309-5.00004-3 PMC3564215

[34] Shadrach KG, Rayborn ME, Hollyfield JG, Bonilha VL. DJ-1-Dependent Regulation of Oxidative Stress in the Retinal Pigment Epithelium (RPE). PLoS ONE. 2013;8: e67983 doi: 10.1371/journal.pone.0067983 23844142 10.1371/journal.pone.0067983 PMC3699467

[35] Boulton ME. Studying melanin and lipofuscin in RPE cell culture models. Exp Eye Res. 2014;126: 61–67. doi: 10.1016/j.exer.2014.01.016 25152361 10.1016/j.exer.2014.01.016 PMC4143628

[36] Ruiz A, Bhat SP, Bok D. Characterization and quantification of full-length and truncated Na,K-ATPase alpha 1 and beta 1 RNA transcripts expressed in human retinal pigment epithelium. Gene. 1995;155: 179–184. 7536695 10.1016/0378-1119(94)00812-7

[37] Philp NJ, Yoon H, Lombardi L. Mouse MCT3 gene is expressed preferentially in retinal pigment and choroid plexus epithelia. Am J Physiol Cell Physiol. 2001;280: C1319–1326. 11287345 10.1152/ajpcell.2001.280.5.C1319

[38] Stamer WD, Bok D, Hu J, Jaffe GJ, McKay BS. Aquaporin-1 channels in human retinal pigment epithelium: role in transepithelial water movement. Invest Ophthalmol Vis Sci. 2003;44: 2803–2808. 12766090 10.1167/iovs.03-0001

[39] Simó R, Villarroel M, Corraliza L, Hernández C, Garcia-Ramírez M, Simó R, et al The Retinal Pigment Epithelium: Something More than a Constituent of the Blood-Retinal Barrier—Implications for the Pathogenesis of Diabetic Retinopathy, The Retinal Pigment Epithelium: Something More than a Constituent of the Blood-Retinal Barrier—Implications for the Pathogenesis of Diabetic Retinopathy. BioMed Res Int BioMed Res Int. 2010;2010, 2010: e190724 doi: 10.1155/2010/190724 20182540 10.1155/2010/190724 PMC2825554

[40] Song MJ, Bharti K. Looking into the future: Using induced pluripotent stem cells to build two and three dimensional ocular tissue for cell therapy and disease modeling. Brain Res. 2015; doi: 10.1016/j.brainres.2015.12.011 26706569 10.1016/j.brainres.2015.12.011 PMC4837038

[41] Thumann G, Kirchhof B. Transplantation of iris pigment epithelium. Ophthalmol Z Dtsch Ophthalmol Ges. 2004;101: 882–885. doi: 10.1007/s00347-004-1084-3 15300389 10.1007/s00347-004-1084-3

[42] Kühl SJ, Kühl M. On the role of Wnt/β-catenin signaling in stem cells. Biochim Biophys Acta BBA—Gen Subj. 2013;1830: 2297–2306. doi: 10.1016/j.bbagen.2012.08.010 22986148 10.1016/j.bbagen.2012.08.010

[43] Sato N, Meijer L, Skaltsounis L, Greengard P, Brivanlou AH. Maintenance of pluripotency in human and mouse embryonic stem cells through activation of Wnt signaling by a pharmacological GSK-3-specific inhibitor. Nat Med. 2004;10: 55–63. doi: 10.1038/nm979 14702635 10.1038/nm979

[44] Miki T, Yasuda S, Kahn M. Wnt/β-catenin signaling in embryonic stem cell self-renewal and somatic cell reprogramming. Stem Cell Rev. 2011;7: 836–846. doi: 10.1007/s12015-011-9275-1 21603945 10.1007/s12015-011-9275-1

[45] Marson A, Foreman R, Chevalier B, Bilodeau S, Kahn M, Young RA, et al Wnt signaling promotes reprogramming of somatic cells to pluripotency. Cell Stem Cell. 2008;3: 132–135. doi: 10.1016/j.stem.2008.06.019 18682236 10.1016/j.stem.2008.06.019 PMC3235673

[46] Ross J, Busch J, Mintz E, Ng D, Stanley A, Brafman D, et al A rare human syndrome provides genetic evidence that WNT signaling is required for reprogramming of fibroblasts to induced pluripotent stem cells. Cell Rep. 2014;9: 1770–1780. doi: 10.1016/j.celrep.2014.10.049 25464842 10.1016/j.celrep.2014.10.049 PMC4335800

[47] Sanges D, Romo N, Simonte G, Di Vicino U, Tahoces AD, Fernández E, et al Wnt/β-Catenin Signaling Triggers Neuron Reprogramming and Regeneration in the Mouse Retina. Cell Rep. 2013;4: 271–286. doi: 10.1016/j.celrep.2013.06.015 23850287 10.1016/j.celrep.2013.06.015

[48] Zhang P, Chang W-H, Fong B, Gao F, Liu C, Al Alam D, et al Regulation of induced pluripotent stem (iPS) cell induction by Wnt/β-catenin signaling. J Biol Chem. 2014;289: 9221–9232. doi: 10.1074/jbc.M113.542845 24482235 10.1074/jbc.M113.542845 PMC3979361

[49] Hägglund A-C, Berghard A, Carlsson L. Canonical Wnt/β-catenin signalling is essential for optic cup formation. PloS One. 2013;8: e81158 doi: 10.1371/journal.pone.0081158 24324671 10.1371/journal.pone.0081158 PMC3852023

[50] Leach LL, Buchholz DE, Nadar VP, Lowenstein SE, Clegg DO. Canonical/β-catenin Wnt pathway activation improves retinal pigmented epithelium derivation from human embryonic stem cells. Invest Ophthalmol Vis Sci. 2015;56: 1002–1013. doi: 10.1167/iovs.14-15835 25604686 10.1167/iovs.14-15835

[51] Westenskow P, Piccolo S, Fuhrmann S. Beta-catenin controls differentiation of the retinal pigment epithelium in the mouse optic cup by regulating Mitf and Otx2 expression. Dev Camb Engl. 2009;136: 2505–2510. doi: 10.1242/dev.032136 19553286 10.1242/dev.032136 PMC2709060

[52] Arnhold S, Semkova I, Andressen C, Lenartz D, Meissner G, Sturm V, et al Iris pigment epithelial cells: a possible cell source for the future treatment of neurodegenerative diseases. Exp Neurol. 2004;187: 410–417. doi: 10.1016/j.expneurol.2004.02.015 15144867 10.1016/j.expneurol.2004.02.015

[53] Asami M, Sun G, Yamaguchi M, Kosaka M. Multipotent cells from mammalian iris pigment epithelium. Dev Biol. 2007;304: 433–446. doi: 10.1016/j.ydbio.2006.12.047 17239846 10.1016/j.ydbio.2006.12.047

[54] Sun G, Asami M, Ohta H, Kosaka J, Kosaka M. Retinal stem/progenitor properties of iris pigment epithelial cells. Dev Biol. 2006;289: 243–252. doi: 10.1016/j.ydbio.2005.10.035 16310762 10.1016/j.ydbio.2005.10.035

[55] Seko Y, Azuma N, Kaneda M, Nakatani K, Miyagawa Y, Noshiro Y, et al Derivation of human differential photoreceptor-like cells from the iris by defined combinations of CRX, RX and NEUROD. PloS One. 2012;7: e35611 doi: 10.1371/journal.pone.0035611 22558175 10.1371/journal.pone.0035611 PMC3338414

[56] Dwyer MA, Kazmin D, Hu P, McDonnell DP, Malek G. Research Resource: Nuclear Receptor Atlas of Human Retinal Pigment Epithelial Cells: Potential Relevance to Age-Related Macular Degeneration. Mol Endocrinol. 2011;25: 360–372. doi: 10.1210/me.2010-0392 21239617 10.1210/me.2010-0392 PMC3386542

[57] Hu P, Herrmann R, Bednar A, Saloupis P, Dwyer MA, Yang P, et al Aryl hydrocarbon receptor deficiency causes dysregulated cellular matrix metabolism and age-related macular degeneration-like pathology. Proc Natl Acad Sci U S A. 2013;110: E4069–4078. doi: 10.1073/pnas.1307574110 24106308 10.1073/pnas.1307574110 PMC3808645

[58] Esfandiary H, Chakravarthy U, Patterson C, Young I, Hughes AE. Association study of detoxification genes in age related macular degeneration. Br J Ophthalmol. 2005;89: 470–474. doi: 10.1136/bjo.2004.047340 15774926 10.1136/bjo.2004.047340 PMC1772608

[59] Kim S-Y, Yang H-J, Chang Y-S, Kim J-W, Brooks M, Chew EY, et al Deletion of Aryl Hydrocarbon Receptor AHR in Mice Leads to Subretinal Accumulation of Microglia and RPE Atrophy. Invest Ophthalmol Vis Sci. 2014;55: 6031–6040. doi: 10.1167/iovs.14-15091 25159211 10.1167/iovs.14-15091 PMC4176417

[60] Choudhary M, Kazmin D, Hu P, Thomas RS, McDonnell DP, Malek G. Aryl hydrocarbon receptor knock-out exacerbates choroidal neovascularization via multiple pathogenic pathways. J Pathol. 2015;235: 101–112. doi: 10.1002/path.4433 25186463 10.1002/path.4433 PMC4277859

[61] Hu DN, Ritch R, McCormick SA, Pelton-Henrion K. Isolation and cultivation of human iris pigment epithelium. Invest Ophthalmol Vis Sci. 1992;33: 2443–2453. 1634342

[62] Abe T, Tomita H, Ohashi T, Yamada K, Takeda Y, Akaishi K, et al Characterization of iris pigment epithelial cell for auto cell transplantation. Cell Transplant. 1999;8: 501–510. 10580344 10.1177/096368979900800505

[63] Mao Y, Finnemann SC. Analysis of photoreceptor outer segment phagocytosis by RPE cells in culture. Methods Mol Biol Clifton NJ. 2013;935: 285–295. doi: 10.1007/978-1-62703-080-9_20 23150376 10.1007/978-1-62703-080-9_20 PMC3590840

[64] Thumann G, Bartz-Schmidt KU, Heimann K, Schraermeyer U. Phagocytosis of rod outer segments by human iris pigment epithelial cells in vitro. Graefes Arch Clin Exp Ophthalmol Albrecht Von Graefes Arch Für Klin Exp Ophthalmol. 1998;236: 753–757. 10.1007/s004170050154 9801890

[65] Abe T, Tomita H, Kano T, Yoshida M, Ohashi T, Nakamura Y, et al Autologous iris pigment epithelial cell transplantation in monkey subretinal region. Curr Eye Res. 2000;20: 268–275. 10806440

[66] Cai H, Shin MC, Tezel TH, Kaplan HJ, Del Priore LV. Use of iris pigment epithelium to replace retinal pigment epithelium in age-related macular degeneration: a gene expression analysis. Arch Ophthalmol Chic Ill 1960. 2006;124: 1276–1285. doi: 10.1001/archopht.124.9.1276 16966623 10.1001/archopht.124.9.1276

[67] Van Soest SS, de Wit GMJ, Essing AHW, ten Brink JB, Kamphuis W, de Jong PTVM, et al Comparison of human retinal pigment epithelium gene expression in macula and periphery highlights potential topographic differences in Bruch’s membrane. Mol Vis. 2007;13: 1608–1617. 17893662

[68] Booij JC, ten Brink JB, Swagemakers SMA, Verkerk AJMH, Essing AHW, van der Spek PJ, et al A new strategy to identify and annotate human RPE-specific gene expression. PloS One. 2010;5: e9341 doi: 10.1371/journal.pone.0009341 20479888 10.1371/journal.pone.0009341 PMC2866542

[69] Smyth GK. Linear models and empirical bayes methods for assessing differential expression in microarray experiments. Stat Appl Genet Mol Biol. 2004;3: Article3. doi: 10.2202/1544-6115.1027 16646809 10.2202/1544-6115.1027

[70] GENE-E. [Internet]. Available: (http://www.broadinstitute.org/cancer/software/GENE-E/.

