# Supplementary figures and images for: Comparative gene expression study and pathway analysis of the human iris- and the retinal pigment epithelium
Source: PLoS One. 2017 Aug 21;12(8):e0182983. doi: 10.1371/journal.pone.0182983 (PMC5565104; doi:10.1371/journal.pone.0182983)

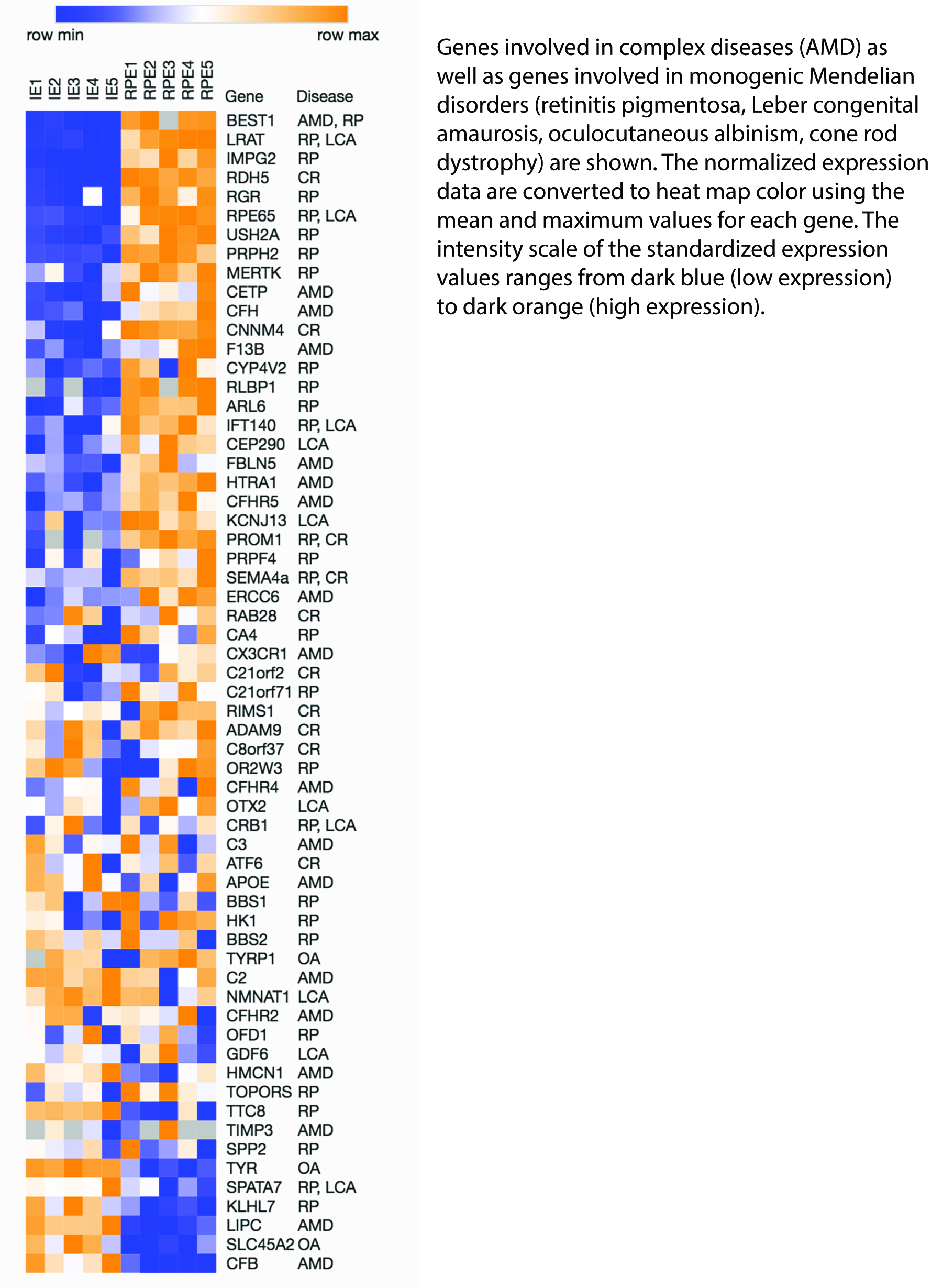

Supplement: S1 Fig — (TIF) [file pone.0182983.s001.tif]

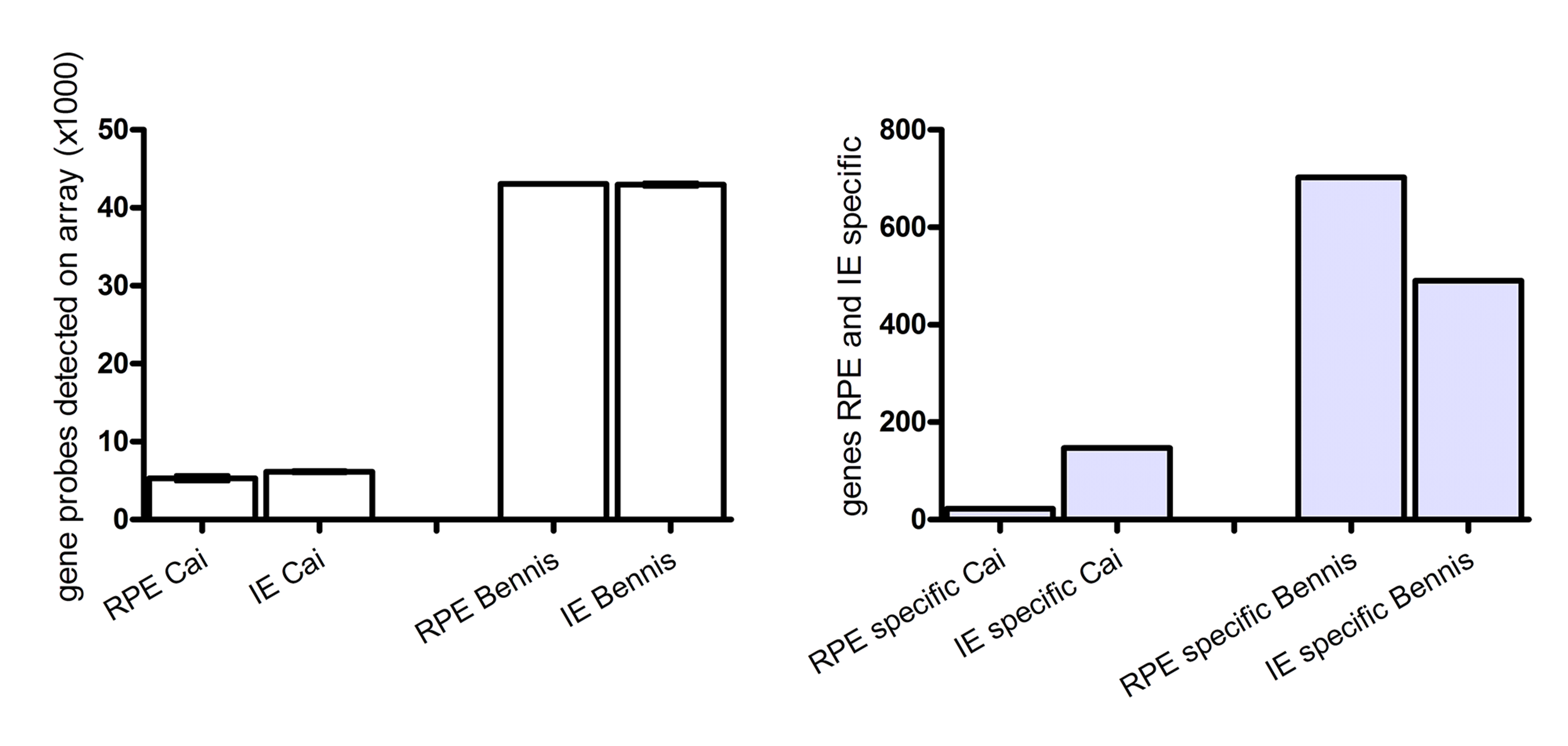

Supplement: S2 Fig — (TIF) [file pone.0182983.s002.tif]

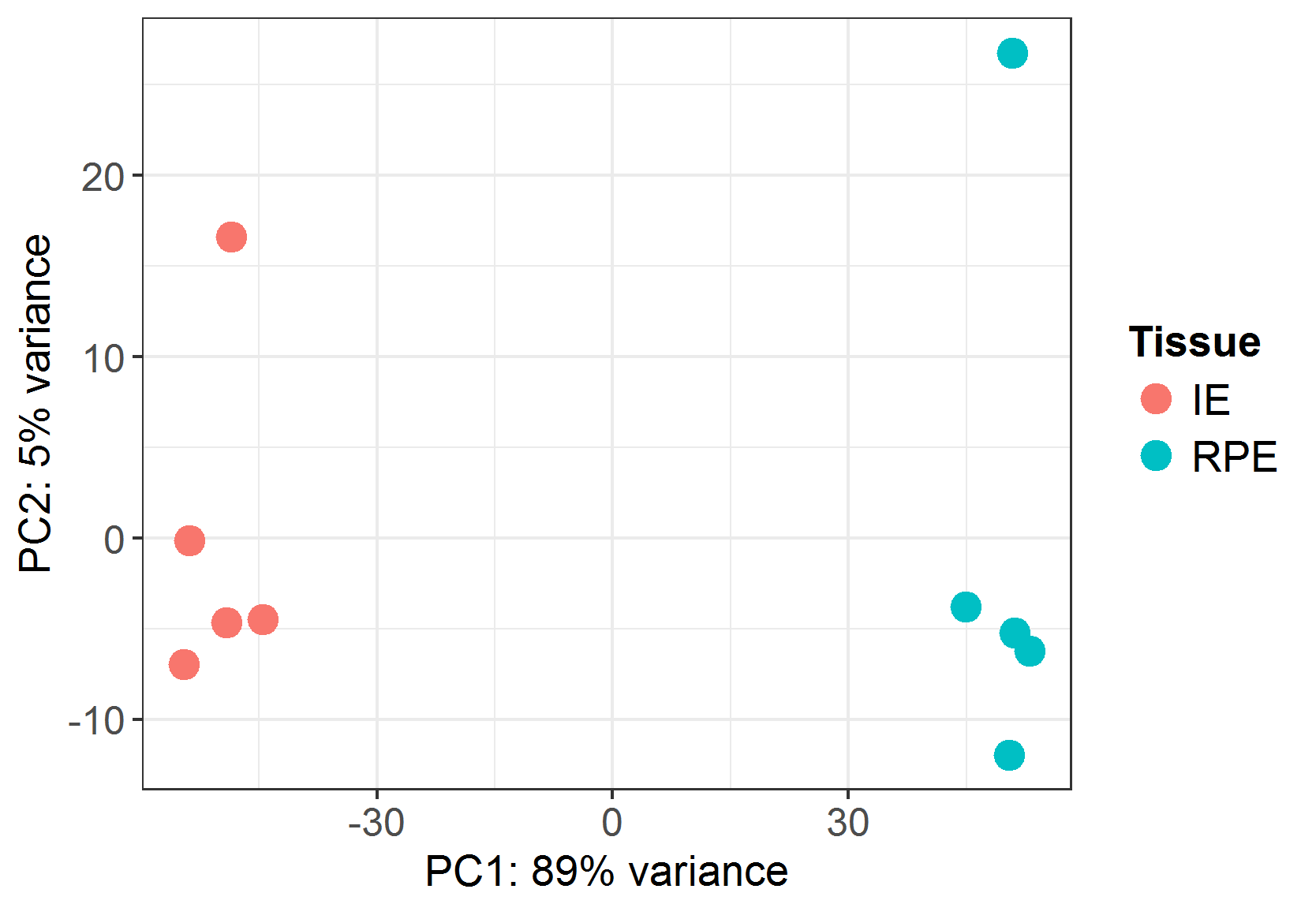

Supplement: S3 Fig — (TIFF) [file pone.0182983.s003.tiff]

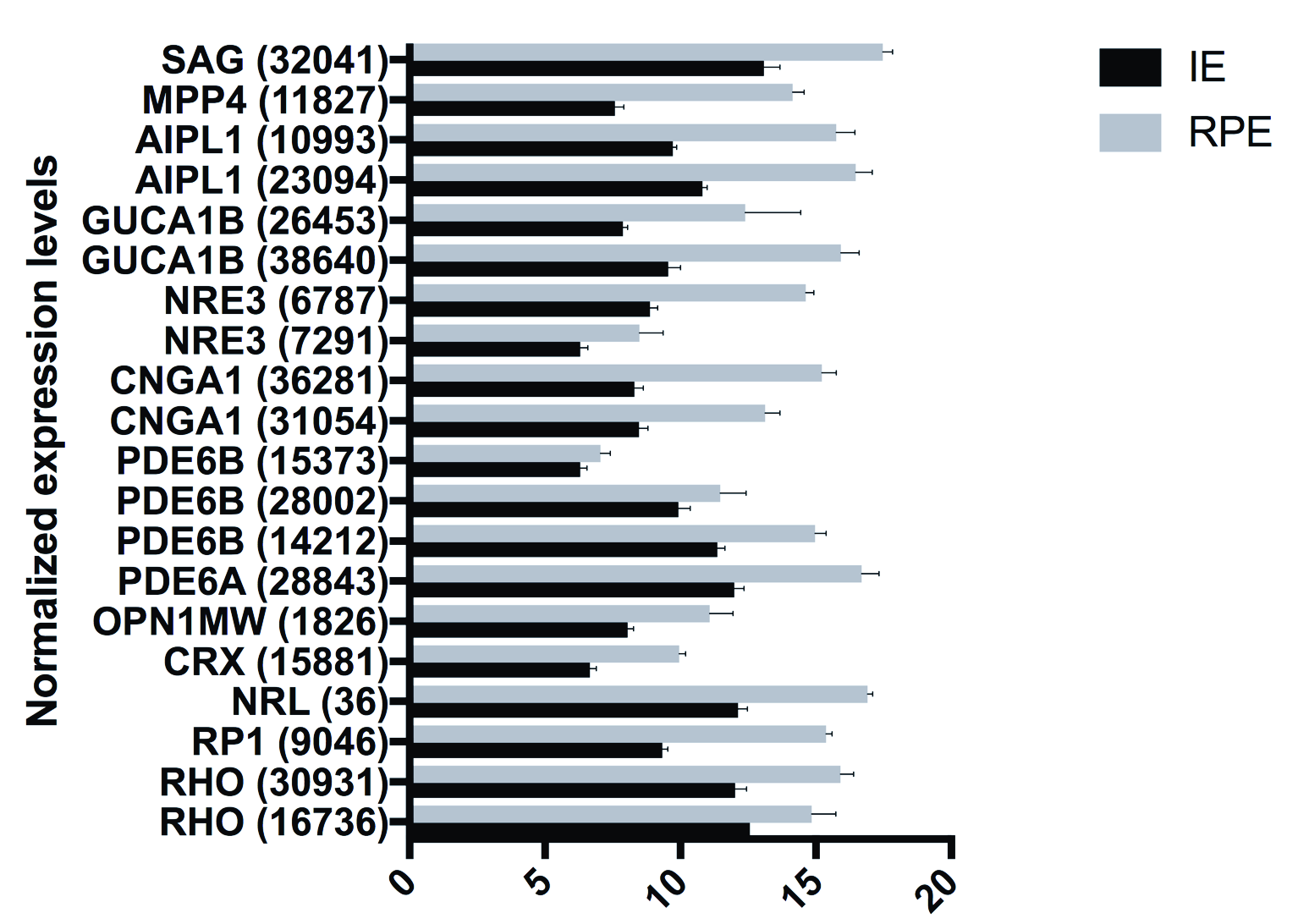

Supplement: S4 Fig — (TIFF) [file pone.0182983.s004.tiff]

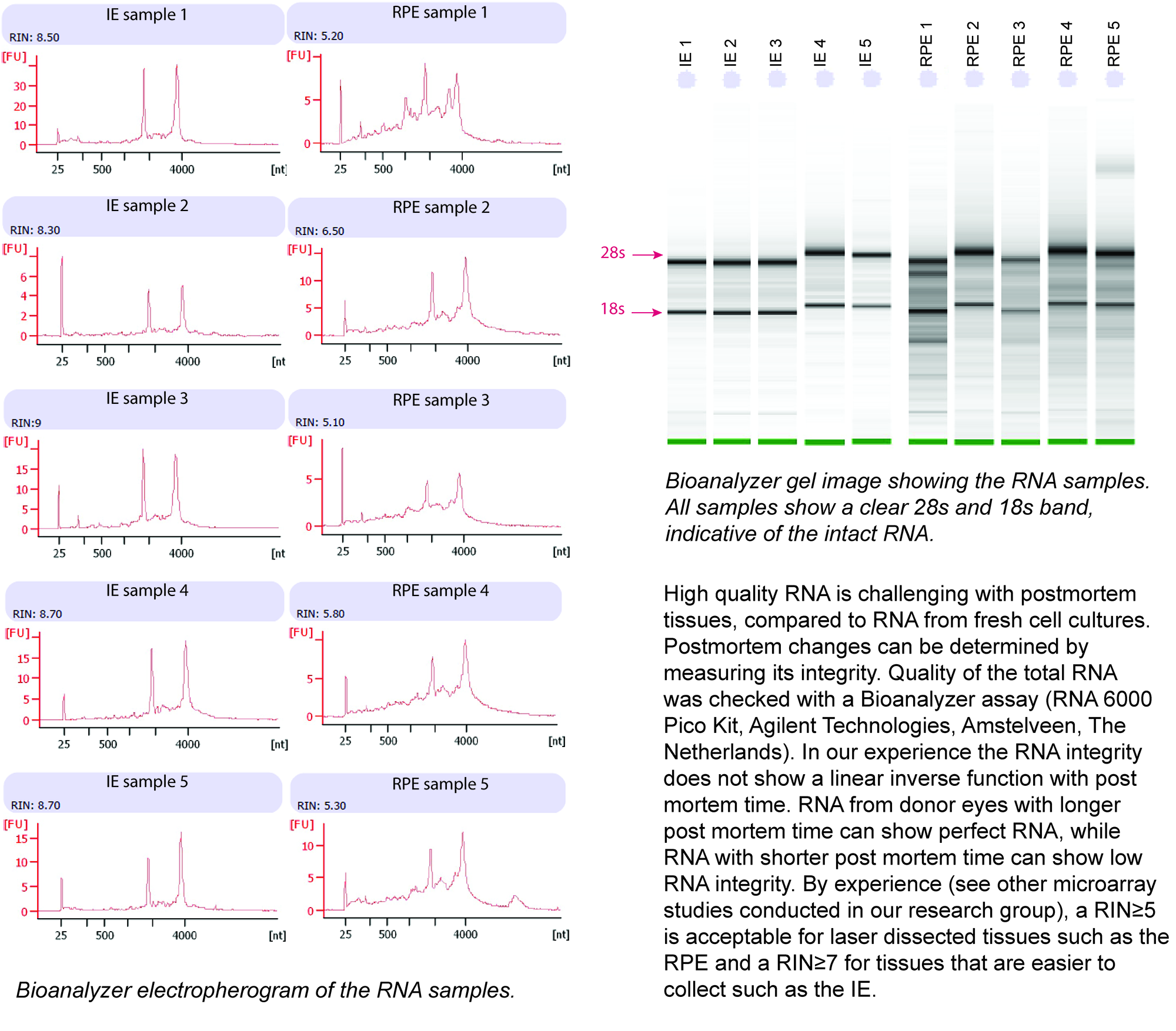

Supplement: S5 Fig — (TIF) [file pone.0182983.s005.tif]

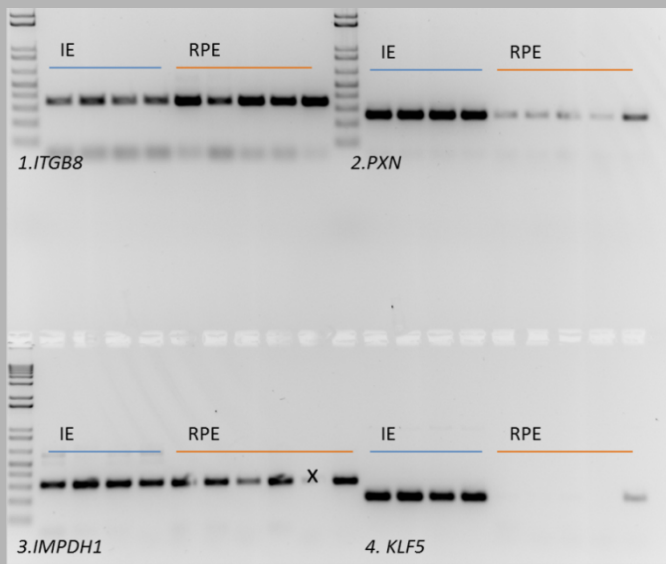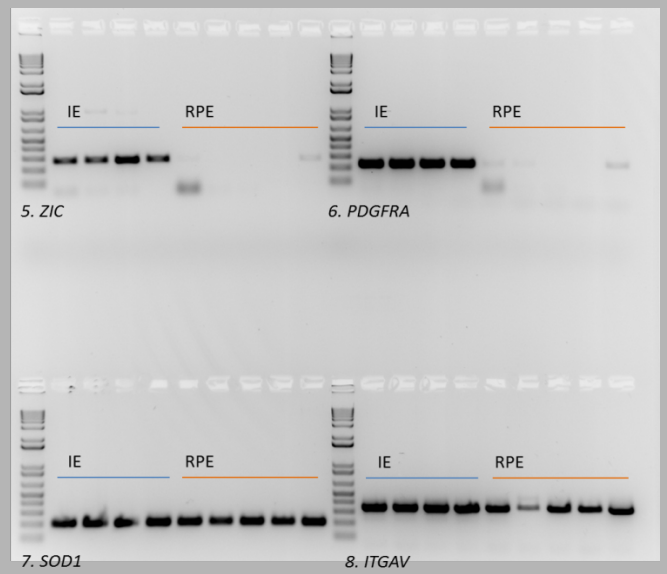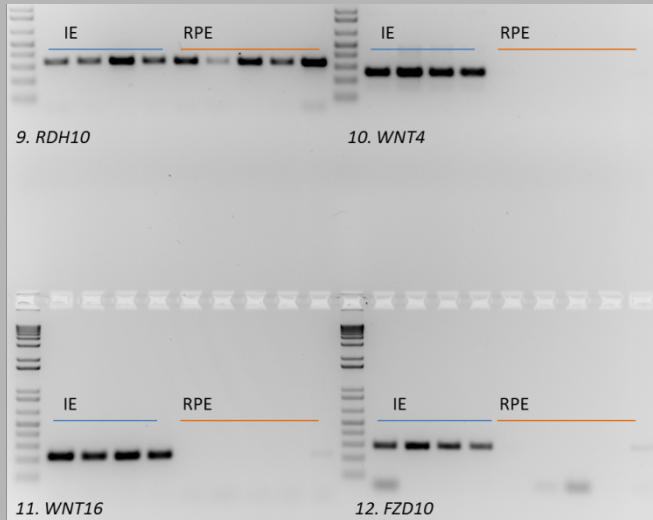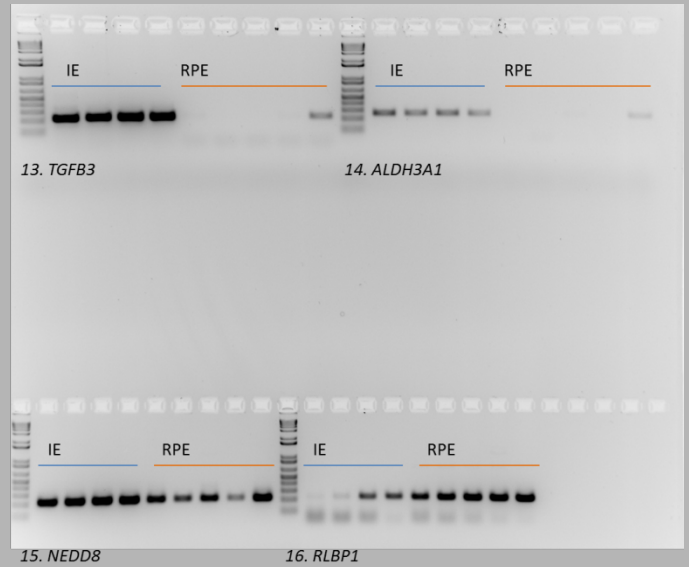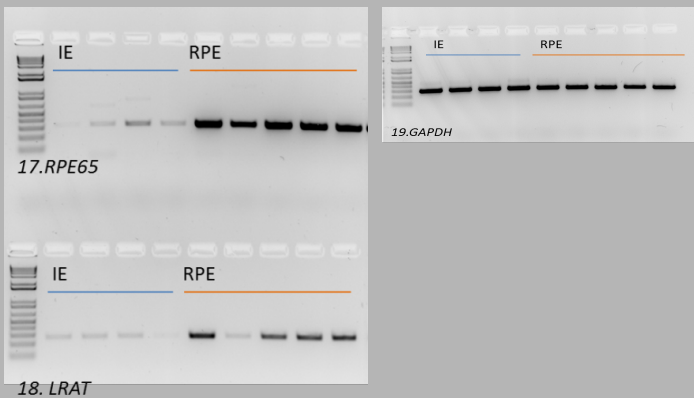

Supplement: S7 Fig — (PDF) [file pone.0182983.s007.pdf]
