# Supplementary material for: Comparative gene expression study and pathway analysis of the human iris- and the retinal pigment epithelium
Source: PLoS One. 2017 Aug 21;12(8):e0182983. doi: 10.1371/journal.pone.0182983 (PMC5565104; doi:10.1371/journal.pone.0182983)
Supplement: S6 Fig — (PDF) [file pone.0182983.s006.pdf]

Human Iris Epithelium Sample 1

QC Report - Agilent Technologies : 2 Color Gene Expression

Date	Thursday, April 16, 2015 - 09:31	BG Method	No Background
Image	US12302316_251485079961_S01_H [1_1]	Background Detrend	On(FeatNCRange, LoPass)
Protocol	GE2-v5_95_Feb07 (Read Only)	Multiplicative Detrend	True
User Name	bennis	Dye Norm	Linear Lowess
Grid	014850_D_20070207	Linear DyeNorm Factor	1.06(Red) 1.01(Green)
FE Version	9.5.3.1	Additive Error	4(Red)3(Green)
		Saturation Value	606400 (r), 586516 (g)

Spot Finding of the Four Corners of the Array

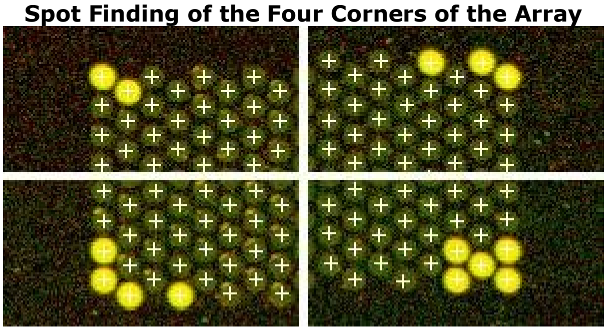

Grid Normal

	Feature Red	Feature Green	Local Background Red	Local Background Green
Non Uniform	12	12	664	87
Population	150	162	2198	1796

Spatial Distribution of All Outliers on the Array

532 rows x 85 columns

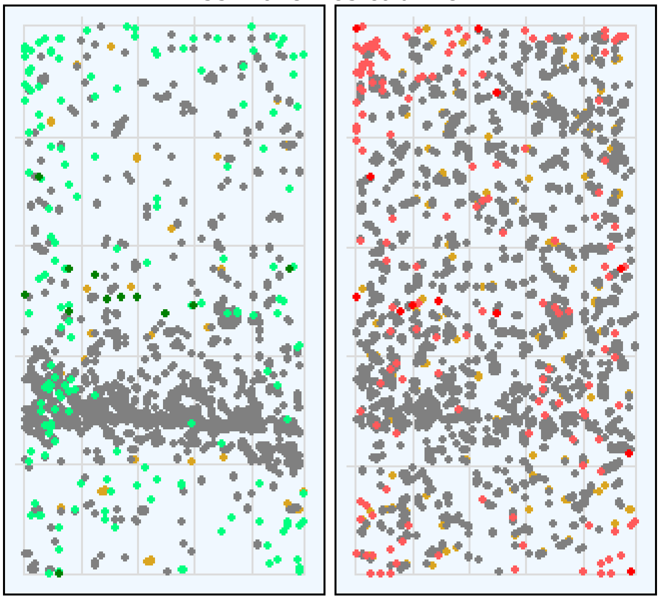

FeatureNonUnif (Red or Green) = 18(0.04%)

GeneNonUnif (Red or Green) = 15 (0.037 %)

BG NonUniform
BG Population
Red FeaturePopulation
Red Feature NonUniform
Green FeaturePopulation
Green Feature NonUniform

Negative Control Stats

	Red	Green
Average Net Signals	47.47	44.84
StdDev Net Signals	2.82	2.15

Net Signal Statistics

Agilent SpikeIns:

	Red	Green
# Saturated Features	0	0
99% of Sig. Distrib.	117	98
50% of Sig. Distrib.	68	63
1% of Sig. Distrib.	50	49

Non-Control probes:

	Red	Green
# Saturated Features	4	0
99% of Sig. Distrib.	217421	59317
50% of Sig. Distrib.	577	805
1% of Sig. Distrib.	49	51

Red and Green Background Corrected Signals (Non-Control Inliers)

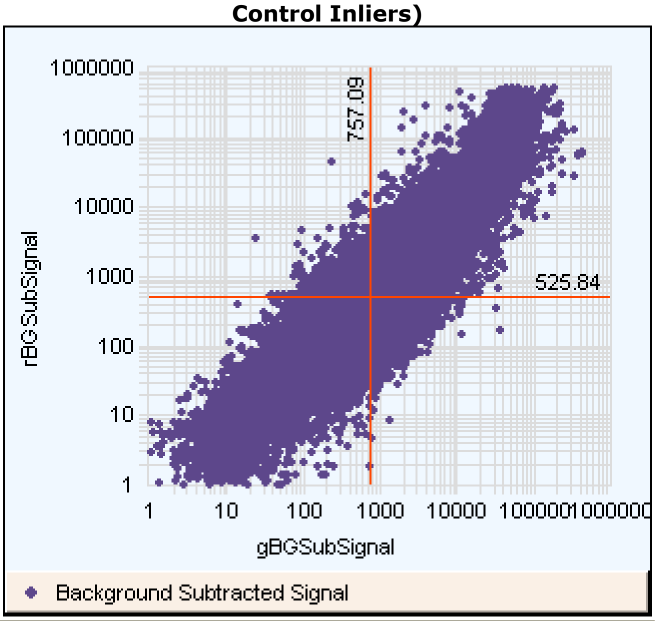

Features (NonCtrl) with BGSubSignals < 0: 771 (Red); 128 (Green)

Average BG Sub Signal	-3.86	-2.83
StdDev BG Sub Signal	2.73	1.99

Local Bkg (inliers)

	Red	Green
Number	42653	43168
Avg	47.18	33.44
SD	3.63	2.45

Foreground Surface Fit

	Red	Green
RMS_Fit	1.15	0.88
RMS_Resid	3.91	2.87
Avg_Fit	68.51	64.34

Reproducibility: %CV for Replicated Probes

	Median %CV Signal (inliers)			
	Non-Control probes		Agilent SpikeIns	
	Red	Green	Red	Green
BGSubSignal	6.39	6.26	-1.00	15.37
ProcessedSignal	3.19	3.82	-1.00	14.68

Array Uniformity: LogRatios

	Non-Control	Agilent SpikeIns
AbsAvgLogRatio	0.27	0.19
AverageS/N	21.88	1.96

Sensitivity:Agilent SpikeIns - Ratio of Signal to Background for 2 dimmest probes

(+)E1A_r60_n11		(+)E1A_r60_a97	
**(g)**	**(r)**	**(g)**	**(r)**
2.1	1.7	1.1	1.2

Spatial Distribution of Significantly Up-Regulated and Down-Regulated Features

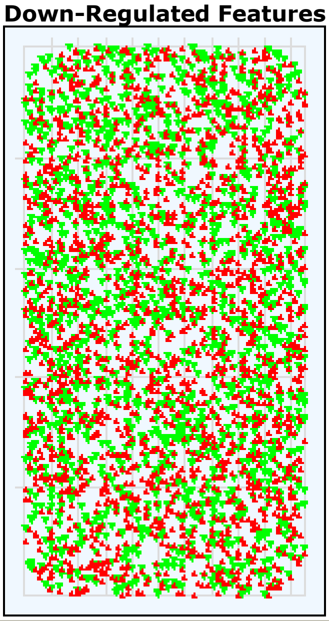

#Up-Regulated:11708 (Red) ; #Down-Regulated:10738 (Green)

Up-Regulated Down-Regulated

LogRatio Versus Log Processed Signal

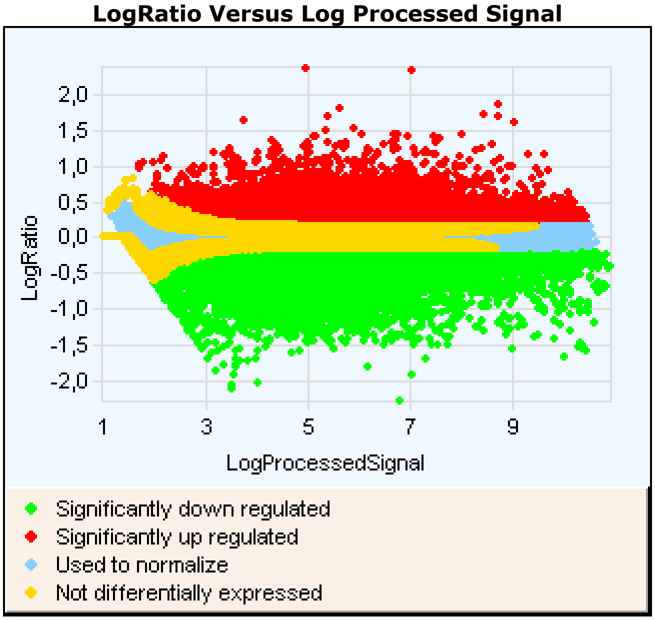

Agilent SpikeIns Signal Statistics

Probe Name	Exp	Obs	SD	S/N
(+)E1A_r60_n9	-1.00	0.08	0.19	0.43
(+)E1A_r60_a107	-0.48	0.14	0.14	1.04
(+)E1A_r60_a135	-0.48	0.15	0.22	0.67
(+)E1A_r60_n11	-0.48	0.40	0.07	5.76
(+)E1A_r60_1	0.00	0.21	0.21	0.97
(+)E1A_r60_a20	0.00	0.25	0.06	4.44
(+)E1A_r60_3	0.48	0.14	0.07	2.09
(+)E1A_r60_a104	0.48	0.34	0.20	1.71
(+)E1A_r60_a97	0.48	0.08	0.19	0.40
(+)E1A_r60_a22	1.00	0.12	0.06	2.06

Agilent SpikeIns: % CV of Average BG Sub Signal

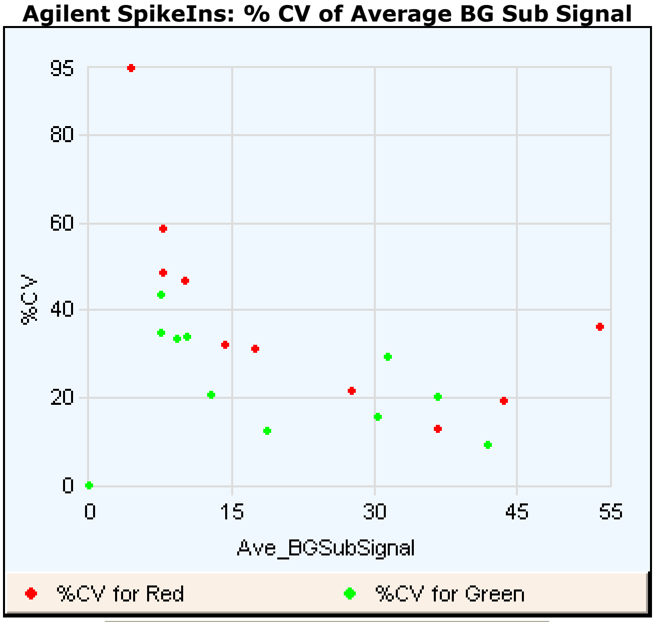

Median %CV:-1.00%(Red);15.37%(Green)

Agilent SpikeIns: Expected LogRatio Vs Observed LogRatio

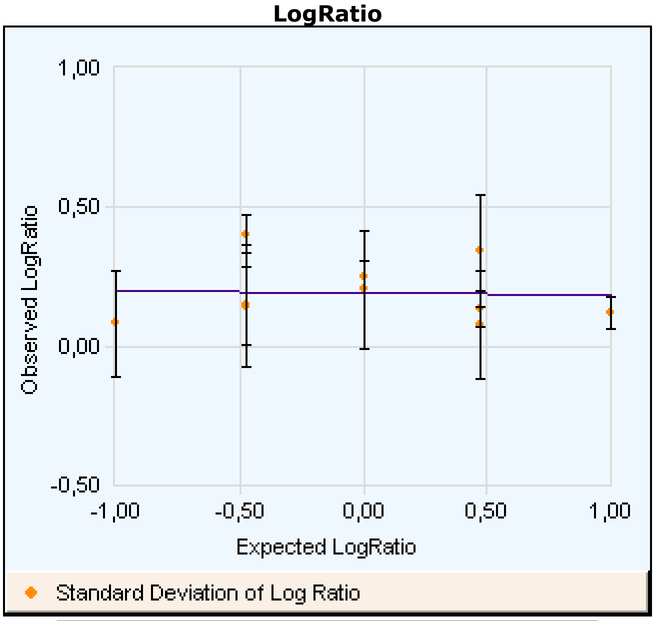

Y-Intercept = 0.191 ; Slope = -0.009 ; R^2 = 0.002

Human Iris Epithelium Sample 2

QC Report - Agilent Technologies : 2 Color Gene Expression

Date	Thursday, April 16, 2015 - 09:36	BG Method	No Background
Image	US12302316_251485079961_S01_H [1_2]	Background Detrend	On(FeatNCRange, LoPass)
Protocol	GE2-v5_95_Feb07 (Read Only)	Multiplicative Detrend	True
User Name	bennis	Dye Norm	Linear Lowess
Grid	014850_D_20070207	Linear DyeNorm Factor	0.832(Red) 1.45(Green)
FE Version	9.5.3.1	Additive Error	4(Red)5(Green)
		Saturation Value	594427 (r), 585587 (g)

Spot Finding of the Four Corners of the Array

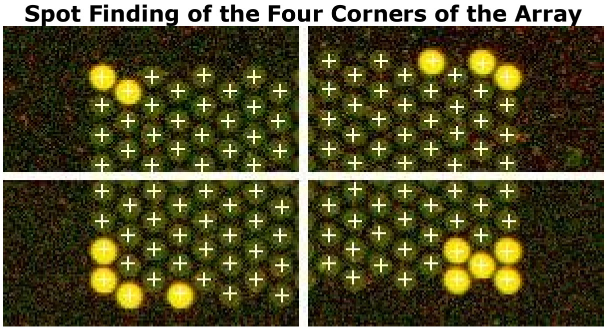

Grid Normal

	Feature Red	Feature Green	Local Background Red	Local Background Green
Non Uniform	12	7	805	16
Population	165	164	2044	938

Spatial Distribution of All Outliers on the Array

532 rows x 85 columns

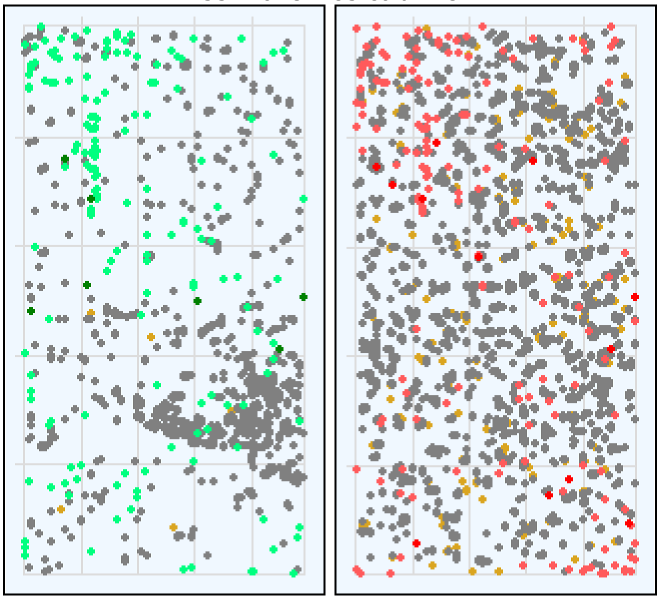

FeatureNonUnif (Red or Green) = 16(0.04%)

GeneNonUnif (Red or Green) = 14 (0.034 %)

- BG NonUniform
- BG Population
- Red FeaturePopulation
- Red Feature NonUniform
- Green FeaturePopulation
- Green Feature NonUniform

Negative Control Stats

	Red	Green
Average Net Signals	50.20	45.62
StdDev Net Signals	3.52	2.28

Net Signal Statistics

Agilent SpikeIns:

	Red	Green
# Saturated Features	0	0
99% of Sig. Distrib.	140	84
50% of Sig. Distrib.	69	58
1% of Sig. Distrib.	51	48

Non-Control probes:

	Red	Green
# Saturated Features	9	0
99% of Sig. Distrib.	270204	39473
50% of Sig. Distrib.	791	636
1% of Sig. Distrib.	52	50

Red and Green Background Corrected Signals (Non-Control Inliers)

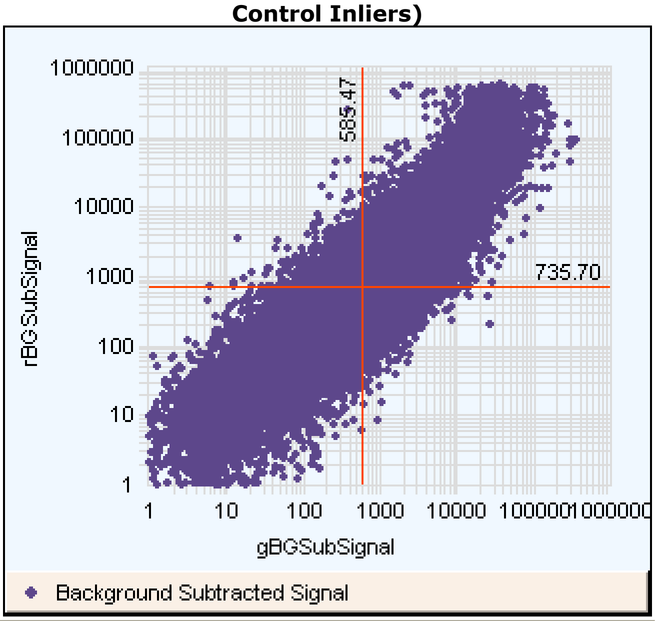

Features (NonCtrl) with BGSubSignals < 0: 870 (Red); 475 (Green)

	Red	Green
Average BG Sub Signal	-5.14	-4.78
StdDev BG Sub Signal	3.42	2.28

Local Bkg (inliers)

	Red	Green
Number	42766	44070
Avg	48.81	35.02
SD	3.91	2.09

Foreground Surface Fit

	Red	Green
RMS_Fit	1.18	0.67
RMS_Resid	4.51	3.14
Avg_Fit	72.53	67.04

Reproducibility: %CV for Replicated Probes

Median %CV Signal (inliers)

	Non-Control probes		Agilent SpikeIns	
	Red	Green	Red	Green
BGSubSignal	8.32	7.89	-1.00	-1.00
ProcessedSignal	3.34	3.89	-1.00	-1.00

Array Uniformity: LogRatios

	Non-Control	Agilent SpikeIns
AbsAvgLogRatio	0.30	0.17
AverageS/N	25.67	1.76

Sensitivity:Agilent SpikeIns - Ratio of Signal to Background for 2 dimmest probes

(+)E1A_r60_n11		(+)E1A_r60_a97	
(g)	(r)	(g)	(r)
2.2	1.4	1.0	1.0

Spatial Distribution of Significantly Up-Regulated and Down-Regulated Features

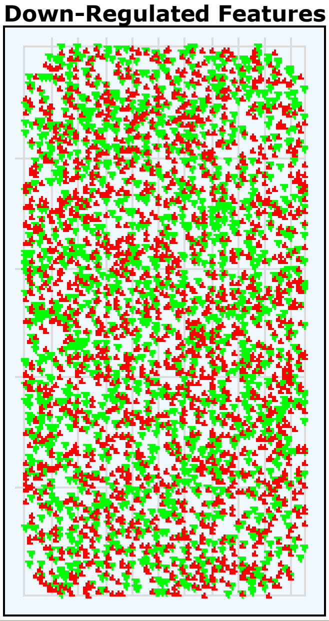

#Up-Regulated:12215 (Red) ; #Down-Regulated:11011 (Green)

Up-Regulated Down-Regulated

LogRatio Versus Log Processed Signal

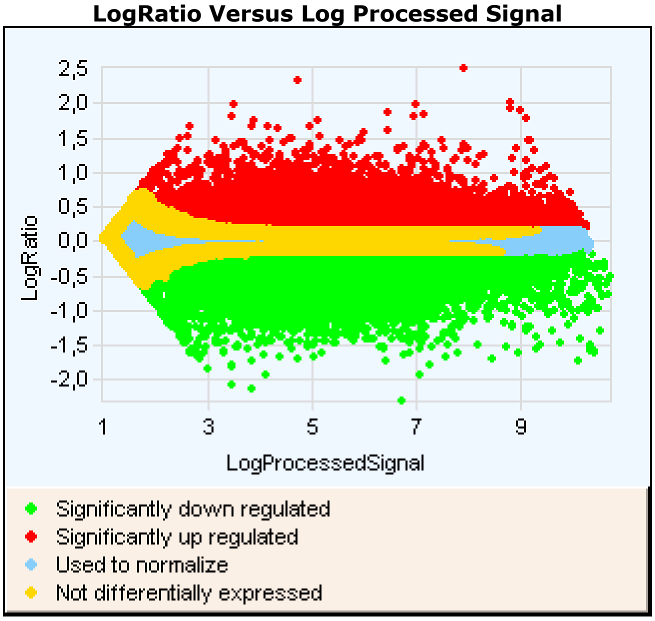

Agilent SpikeIns Signal Statistics

Probe Name	Exp	Obs	SD	S/N
(+)E1A_r60_n9	-1.00	0.02	0.21	0.11
(+)E1A_r60_a107	-0.48	0.07	0.20	0.34
(+)E1A_r60_a135	-0.48	0.06	0.24	0.26
(+)E1A_r60_n11	-0.48	0.46	0.06	7.64
(+)E1A_r60_1	0.00	0.31	0.17	1.76
(+)E1A_r60_a20	0.00	0.19	0.08	2.39
(+)E1A_r60_3	0.48	0.17	0.10	1.80
(+)E1A_r60_a104	0.48	0.33	0.19	1.77
(+)E1A_r60_a97	0.48	-0.02	0.07	0.32
(+)E1A_r60_a22	1.00	0.10	0.08	1.24

Agilent SpikeIns: % CV of Average BG Sub Signal

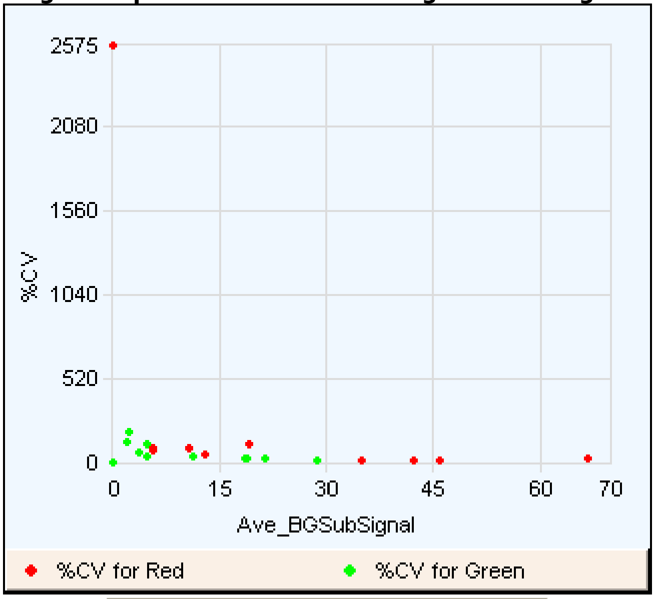

Median %CV:-1.00%(Red);-1.00%(Green)

Agilent SpikeIns: Expected LogRatio Vs Observed LogRatio

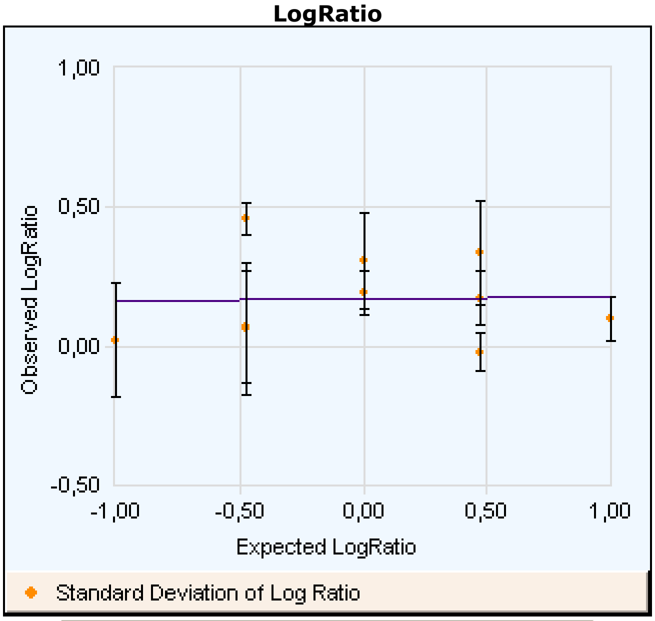

Y-Intercept = 0.170 ; Slope = 0.009 ; R^2 = 0.001

Human Iris Epithelium Sample 3

QC Report - Agilent Technologies : 2 Color Gene Expression

Date	Thursday, April 16, 2015 - 09:40	BG Method	No Background
Image	US12302316_251485079961_S01_H [1_3]	Background Detrend	On(FeatNCRange, LoPass)
Protocol	GE2-v5_95_Feb07 (Read Only)	Multiplicative Detrend	True
User Name	bennis	Dye Norm	Linear Lowess
Grid	014850_D_20070207	Linear DyeNorm Factor	1.05(Red) 1.42(Green)
FE Version	9.5.3.1	Additive Error	4(Red)4(Green)
		Saturation Value	588507 (r), 585910 (g)

Spot Finding of the Four Corners of the Array

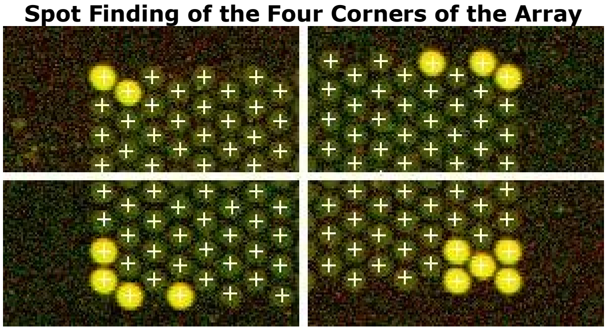

Grid Normal

	Feature Red	Feature Green	Local Background Red	Local Background Green
Non Uniform	9	8	316	20
Population	172	161	1661	768

Spatial Distribution of All Outliers on the Array

532 rows x 85 columns

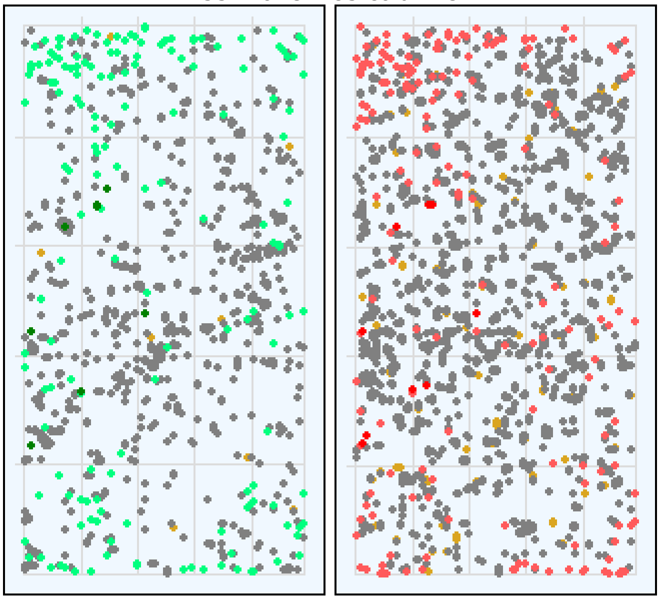

FeatureNonUnif (Red or Green) = 11(0.02%)

GeneNonUnif (Red or Green) = 11 (0.027 %)

- BG NonUniform
- BG Population
- Red FeaturePopulation
- Red Feature NonUniform
- Green FeaturePopulation
- Green Feature NonUniform

Negative Control Stats

	Red	Green
Average Net Signals	46.93	47.55
StdDev Net Signals	2.85	1.98

Net Signal Statistics

Agilent SpikeIns:

	Red	Green
# Saturated Features	0	0
99% of Sig. Distrib.	112	87
50% of Sig. Distrib.	64	60
1% of Sig. Distrib.	46	49

Non-Control probes:

	Red	Green
# Saturated Features	4	0
99% of Sig. Distrib.	213834	48595
50% of Sig. Distrib.	678	649
1% of Sig. Distrib.	48	52

Red and Green Background Corrected Signals (Non-Control Inliers)

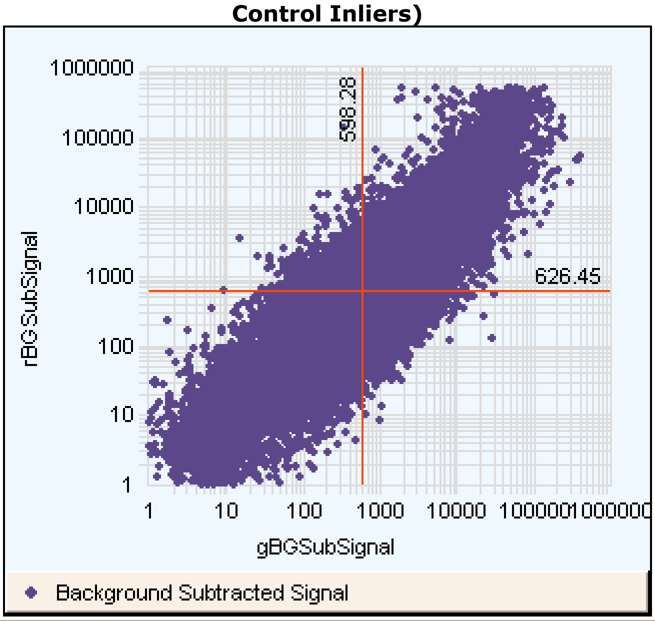

Features (NonCtrl) with BGSubSignals < 0: 952 (Red); 312 (Green)

Average BG Sub Signal	-4.32	-3.76
StdDev BG Sub Signal	2.77	2.05

Local Bkg (inliers)

	Red	Green
Number	43248	44235
Avg	46.55	37.11
SD	3.25	2.08

Foreground Surface Fit

	Red	Green
RMS_Fit	1.12	0.52
RMS_Resid	3.76	2.78
Avg_Fit	68.46	67.94

Reproducibility: %CV for Replicated Probes

	Median %CV Signal (inliers)			
	Non-Control probes		Agilent SpikeIns	
	Red	Green	Red	Green
BGSubSignal	7.38	6.81	-1.00	-1.00
ProcessedSignal	3.21	3.59	-1.00	-1.00

Array Uniformity: LogRatios

	Non-Control	Agilent SpikeIns
AbsAvgLogRatio	0.32	0.16
AverageS/N	30.42	1.29

Sensitivity:Agilent SpikeIns - Ratio of Signal to Background for 2 dimmest probes

(+)E1A_r60_n11		(+)E1A_r60_a97	
**(g)**	**(r)**	**(g)**	**(r)**
1.9	1.4	1.0	1.1

Spatial Distribution of Significantly Up-Regulated and Down-Regulated Features

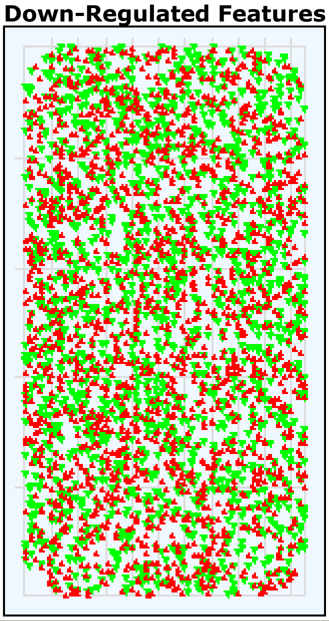

#Up-Regulated:12549 (Red) ; #Down-Regulated:11429 (Green)

Up-Regulated Down-Regulated

LogRatio Versus Log Processed Signal

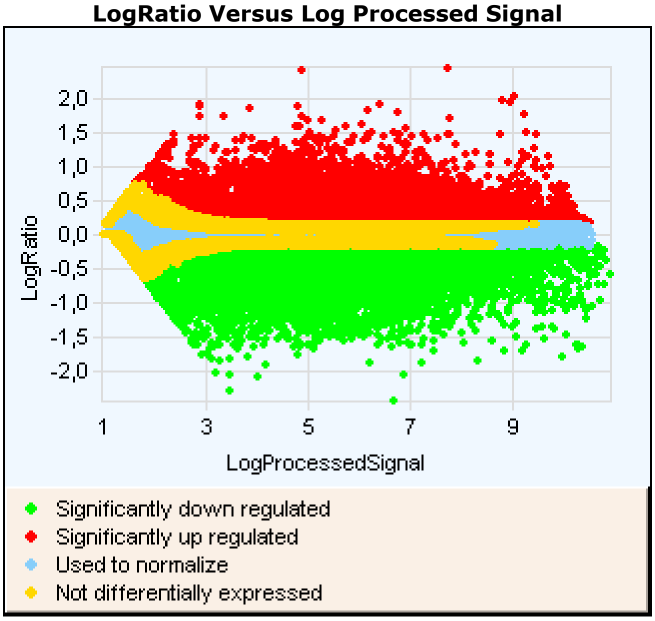

Agilent SpikeIns Signal Statistics

Probe Name	Exp	Obs	SD	S/N
(+)E1A_r60_n9	-1.00	-0.01	0.24	0.06
(+)E1A_r60_a107	-0.48	0.06	0.19	0.30
(+)E1A_r60_a135	-0.48	0.09	0.23	0.41
(+)E1A_r60_n11	-0.48	0.35	0.12	2.80
(+)E1A_r60_1	0.00	0.20	0.20	1.01
(+)E1A_r60_a20	0.00	0.24	0.08	3.08
(+)E1A_r60_3	0.48	0.18	0.08	2.34
(+)E1A_r60_a104	0.48	0.35	0.22	1.56
(+)E1A_r60_a97	0.48	-0.04	0.10	0.36
(+)E1A_r60_a22	1.00	0.06	0.06	0.97

Agilent SpikeIns: % CV of Average BG Sub Signal

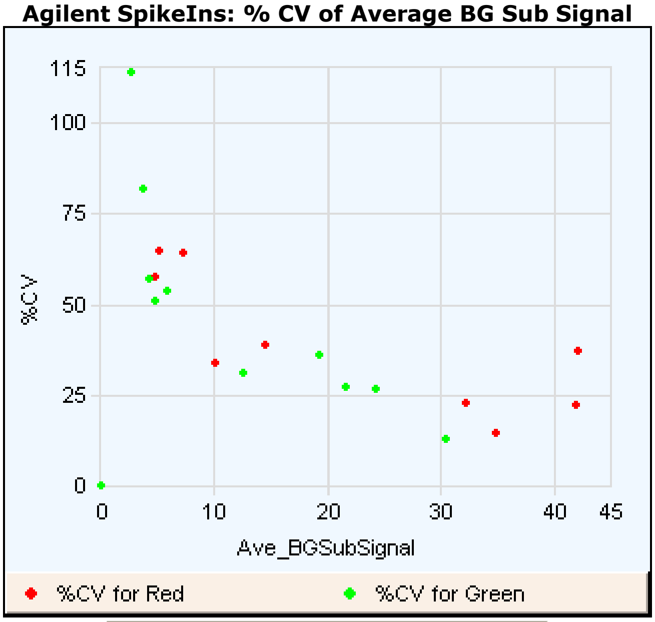

Median %CV:-1.00%(Red);-1.00%(Green)

Agilent SpikeIns: Expected LogRatio Vs Observed LogRatio

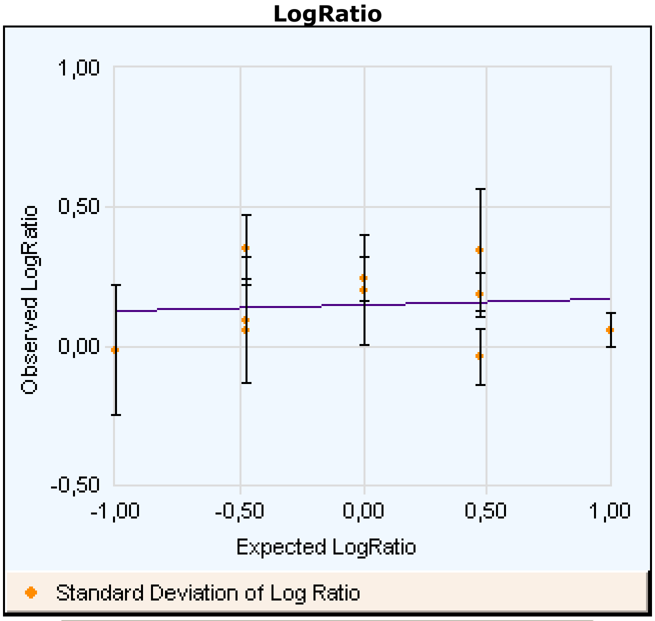

Y-Intercept = 0.148 ; Slope = 0.021 ; R^2 = 0.008

Human Iris Epithelium Sample 4

QC Report - Agilent Technologies : 2 Color Gene Expression

Date	Thursday, April 16, 2015 - 09:44	BG Method	No Background
Image	US12302316_251485079961_S01_H [1_4]	Background Detrend	On(FeatNCRange, LoPass)
Protocol	GE2-v5_95_Feb07 (Read Only)	Multiplicative Detrend	True
User Name	bennis	Dye Norm	Linear Lowess
Grid	014850_D_20070207	Linear DyeNorm Factor	0.967(Red) 1.42(Green)
FE Version	9.5.3.1	Additive Error	4(Red)5(Green)
		Saturation Value	588298 (r), 585172 (g)

Spot Finding of the Four Corners of the Array

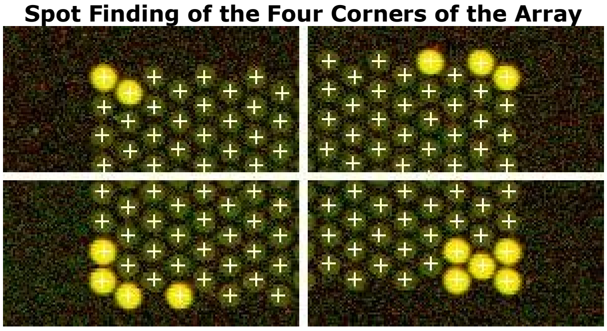

Grid Normal

	Feature Red	Feature Green	Local Background Red	Local Background Green
Non Uniform	11	11	402	21
Population	206	252	1780	773

Spatial Distribution of All Outliers on the Array

532 rows x 85 columns

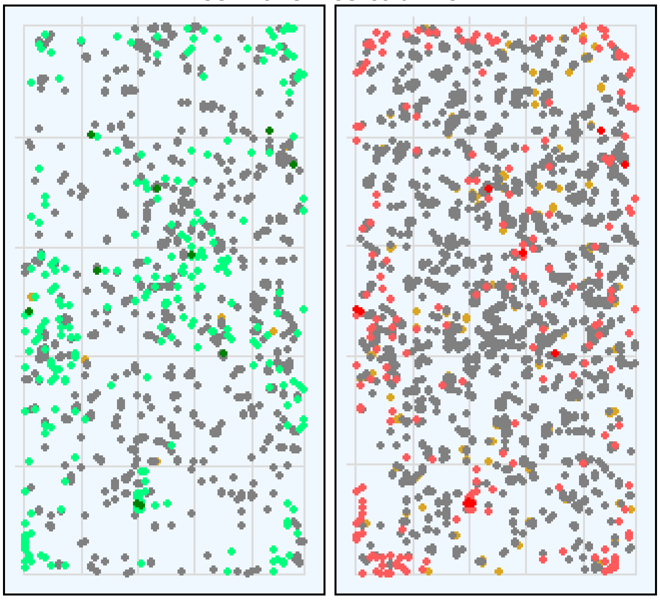

FeatureNonUnif (Red or Green) = 13(0.03%)

GeneNonUnif (Red or Green) = 13 (0.032 %)

- BG NonUniform
- BG Population
- Red FeaturePopulation
- Red Feature NonUniform
- Green FeaturePopulation
- Green Feature NonUniform

Negative Control Stats

	Red	Green
Average Net Signals	48.64	48.38
StdDev Net Signals	3.92	2.56

Net Signal Statistics

Agilent SpikeIns:

	Red	Green
# Saturated Features	0	0
99% of Sig. Distrib.	130	106
50% of Sig. Distrib.	69	63
1% of Sig. Distrib.	47	51

Non-Control probes:

	Red	Green
# Saturated Features	8	0
99% of Sig. Distrib.	216726	44017
50% of Sig. Distrib.	710	627
1% of Sig. Distrib.	50	53

Red and Green Background Corrected Signals (Non-Control Inliers)

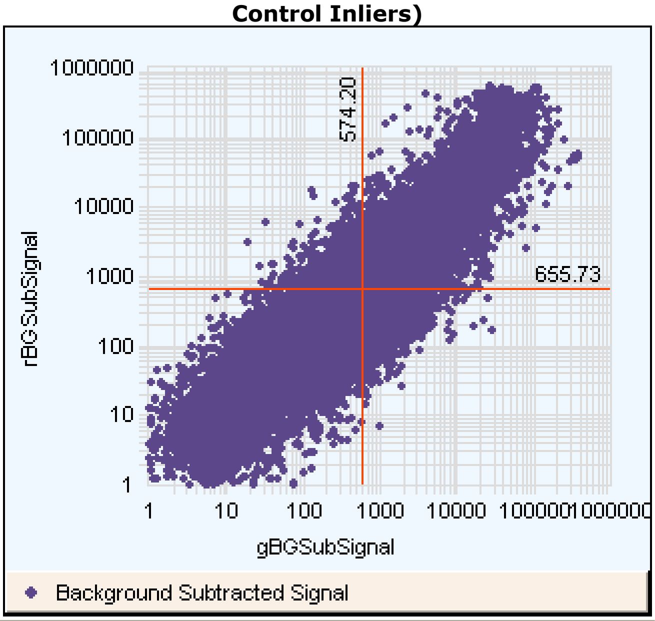

Features (NonCtrl) with BGSubSignals < 0: 865 (Red); 416 (Green)

Average BG Sub Signal	-4.95	-5.04
StdDev BG Sub Signal	3.63	2.50

Local Bkg (inliers)

	Red	Green
Number	43098	44230
Avg	46.49	37.46
SD	3.30	2.00

Foreground Surface Fit

	Red	Green
RMS_Fit	1.56	1.16
RMS_Resid	4.64	3.28
Avg_Fit	70.83	70.06

Reproducibility: %CV for Replicated Probes

	Median %CV Signal (inliers)			
	Non-Control probes		Agilent SpikeIns	
	Red	Green	Red	Green
BGSubSignal	6.51	5.67	-1.00	-1.00
ProcessedSignal	4.04	4.48	-1.00	-1.00

Array Uniformity: LogRatios

	Non-Control	Agilent SpikeIns
AbsAvgLogRatio	0.29	0.18
AverageS/N	21.10	1.47

Sensitivity:Agilent SpikeIns - Ratio of Signal to Background for 2 dimmest probes

(+)E1A_r60_n11		(+)E1A_r60_a97	
**(g)**	**(r)**	**(g)**	**(r)**
2.1	1.3	1.0	1.0

Spatial Distribution of Significantly Up-Regulated and Down-Regulated Features

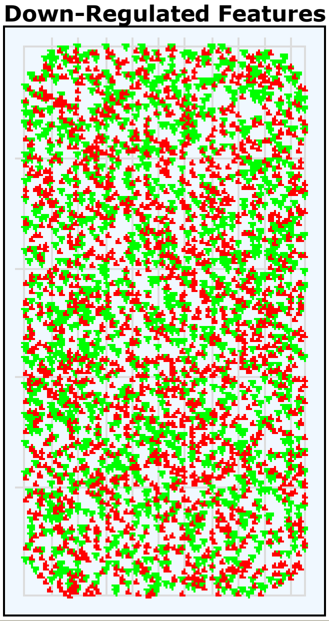

#Up-Regulated:11738 (Red) ; #Down-Regulated:10656 (Green)

Up-Regulated Down-Regulated

LogRatio Versus Log Processed Signal

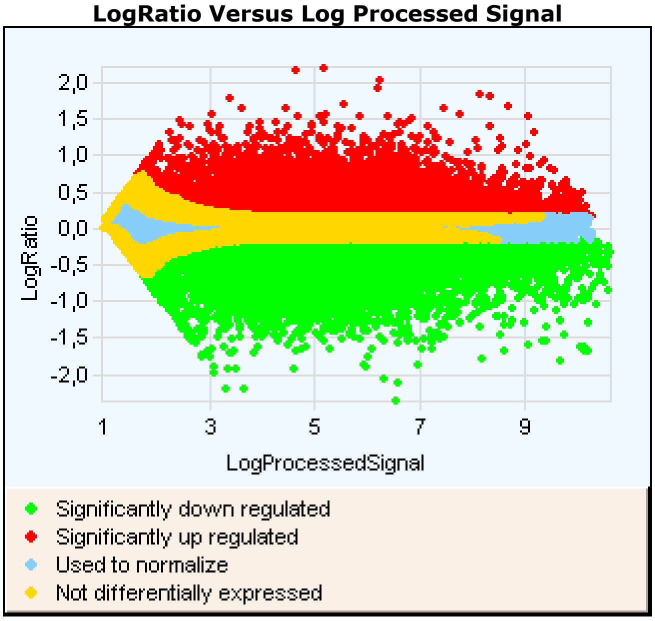

Agilent SpikeIns Signal Statistics

Probe Name	Exp	Obs	SD	S/N
(+)E1A_r60_n9	-1.00	0.03	0.22	0.13
(+)E1A_r60_a107	-0.48	0.11	0.14	0.74
(+)E1A_r60_a135	-0.48	0.11	0.20	0.55
(+)E1A_r60_n11	-0.48	0.40	0.09	4.34
(+)E1A_r60_1	0.00	0.25	0.21	1.18
(+)E1A_r60_a20	0.00	0.20	0.06	3.41
(+)E1A_r60_3	0.48	0.15	0.10	1.48
(+)E1A_r60_a104	0.48	0.40	0.22	1.84
(+)E1A_r60_a97	0.48	-0.05	0.21	0.22
(+)E1A_r60_a22	1.00	0.08	0.10	0.83

Agilent SpikeIns: % CV of Average BG Sub Signal

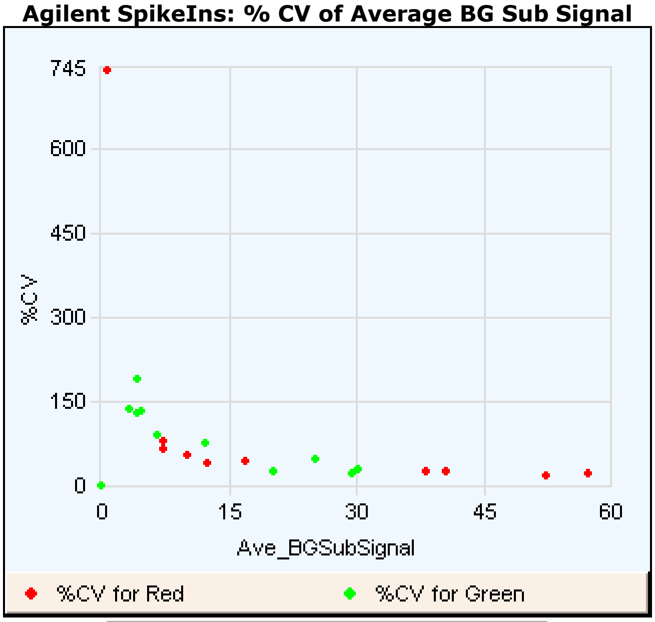

Median %CV:-1.00%(Red);-1.00%(Green)

Agilent SpikeIns: Expected LogRatio Vs Observed LogRatio

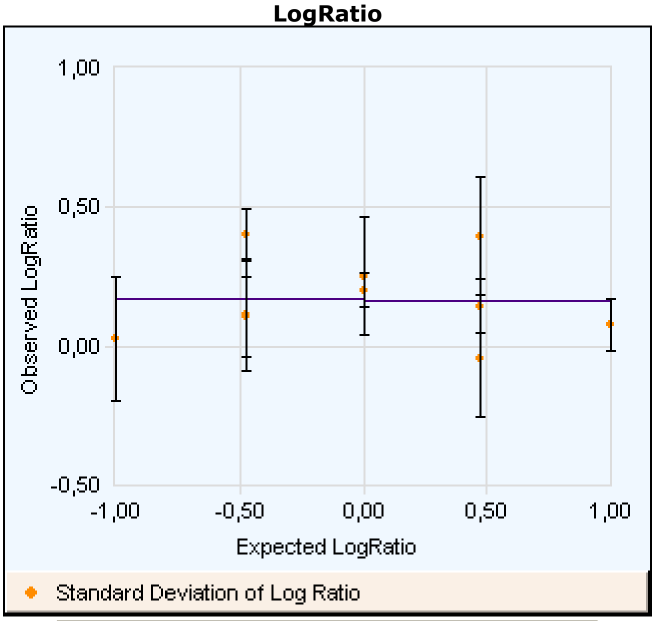

Y-Intercept = 0.168 ; Slope = -0.003 ; R^2 = 0.000

Human Iris Epithelium Sample 5

QC Report - Agilent Technologies : 2 Color Gene Expression

Date	Thursday, April 16, 2015 - 11:23	BG Method	No Background
Image	US12302316_251485079963_S01_H [1_2]	Background Detrend	On(FeatNCRange, LoPass)
Protocol	GE2-v5_95_Feb07 (Read Only)	Multiplicative Detrend	True
User Name	bennis	Dye Norm	Linear Lowess
Grid	014850_D_20070207	Linear DyeNorm Factor	2.4(Red) 1.1(Green)
FE Version	9.5.3.1	Additive Error	5(Red)2(Green)
		Saturation Value	569872 (r), 587750 (g)

Spot Finding of the Four Corners of the Array

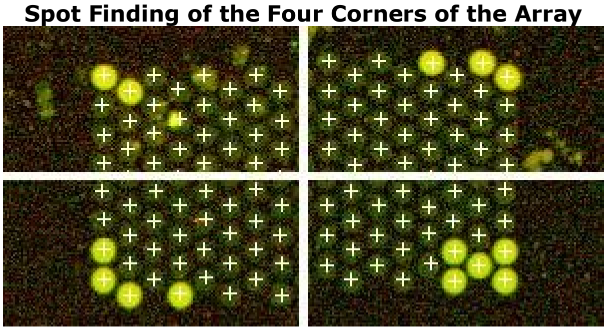

Grid Normal

	Feature Red	Feature Green	Local Background Red	Local Background Green
Non Uniform	19	18	28	85
Population	119	173	1046	757

Spatial Distribution of All Outliers on the Array

532 rows x 85 columns

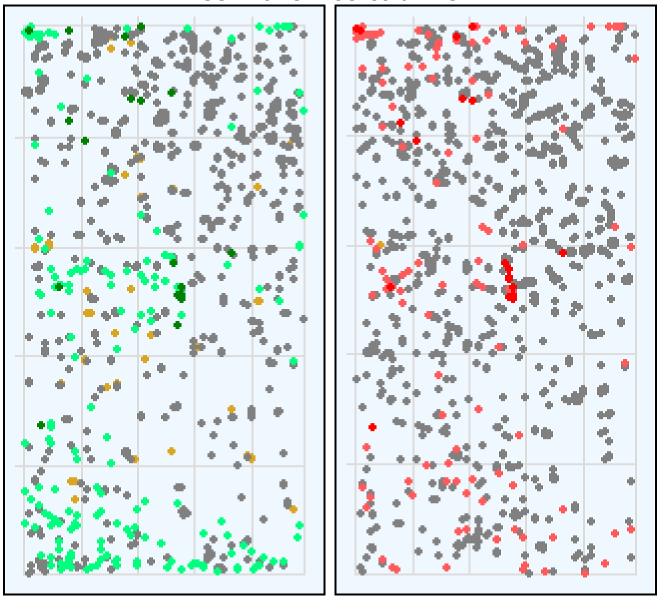

FeatureNonUnif (Red or Green) = 22(0.05%)

GeneNonUnif (Red or Green) = 20 (0.049 %)

BG NonUniform, BG Population, Red FeaturePopulation, Red Feature NonUniform, Green FeaturePopulation, Green Feature NonUniform

Negative Control Stats

	Red	Green
Average Net Signals	34.32	33.56
StdDev Net Signals	2.19	3.06

Net Signal Statistics

Agilent SpikeIns:

	Red	Green
# Saturated Features	0	0
99% of Sig. Distrib.	65	74
50% of Sig. Distrib.	39	44
1% of Sig. Distrib.	31	32

Non-Control probes:

	Red	Green
# Saturated Features	0	0
99% of Sig. Distrib.	51943	53513
50% of Sig. Distrib.	165	443
1% of Sig. Distrib.	33	36

Red and Green Background Corrected Signals (Non-Control Inliers)

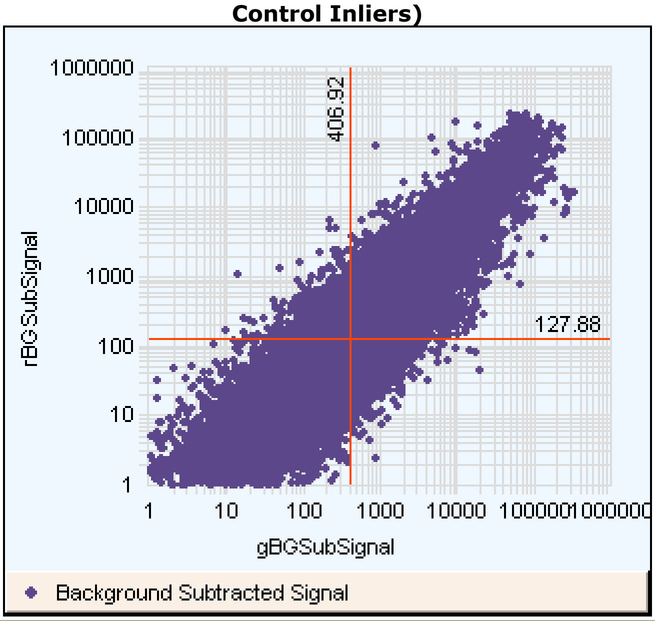

Features (NonCtrl) with BGSubSignals < 0: 1563 (Red); 342 (Green)

Average BG Sub Signal	-2.11	-2.67
StdDev BG Sub Signal	1.65	1.90

Local Bkg (inliers)

	Red	Green
Number	43968	44211
Avg	42.72	31.47
SD	2.37	1.98

Foreground Surface Fit

	Red	Green
RMS_Fit	1.48	2.45
RMS_Resid	1.99	2.13
Avg_Fit	53.48	53.32

Reproducibility: %CV for Replicated Probes

	Median %CV Signal (inliers)			
	Non-Control probes		Agilent SpikeIns	
	Red	Green	Red	Green
BGSubSignal	26.88	8.50	-1.00	-1.00
ProcessedSignal	6.31	3.95	-1.00	-1.00

Array Uniformity: LogRatios

	Non-Control	Agilent SpikeIns
AbsAvgLogRatio	0.26	0.10
AverageS/N	10.84	0.55

Sensitivity:Agilent SpikeIns - Ratio of Signal to Background for 2 dimmest probes

(+)E1A_r60_n11		(+)E1A_r60_a97	
**(g)**	**(r)**	**(g)**	**(r)**
1.3	1.4	1.0	1.0

Spatial Distribution of Significantly Up-Regulated and Down-Regulated Features

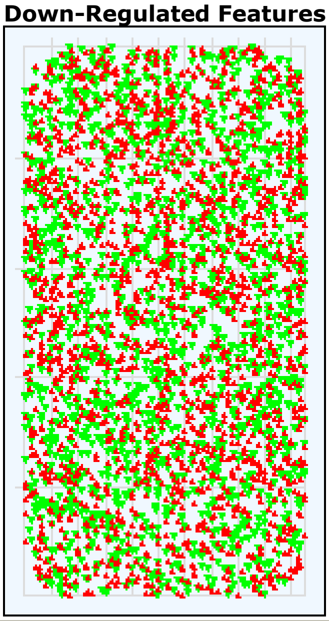

#Up-Regulated:10518 (Red) ; #Down-Regulated:9873 (Green)

Up-Regulated Down-Regulated

LogRatio Versus Log Processed Signal

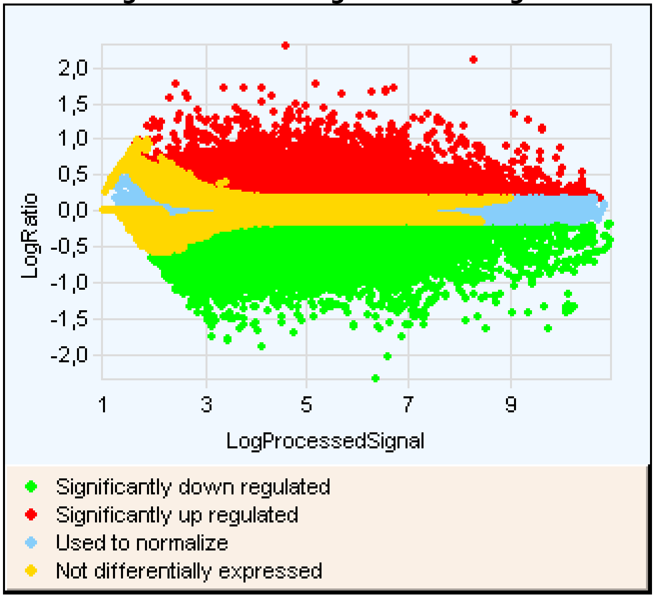

Agilent SpikeIns Signal Statistics

Probe Name	Exp	Obs	SD	S/N
(+)E1A_r60_n9	-1.00	0.07	0.18	0.40
(+)E1A_r60_a107	-0.48	0.03	0.21	0.12
(+)E1A_r60_a135	-0.48	0.09	0.20	0.45
(+)E1A_r60_n11	-0.48	0.38	0.16	2.41
(+)E1A_r60_1	0.00	0.07	0.20	0.37
(+)E1A_r60_a20	0.00	-0.01	0.15	0.09
(+)E1A_r60_3	0.48	0.09	0.20	0.46
(+)E1A_r60_a104	0.48	0.16	0.26	0.62
(+)E1A_r60_a97	0.48	0.04	0.12	0.29
(+)E1A_r60_a22	1.00	0.05	0.20	0.26

Agilent SpikeIns: % CV of Average BG Sub Signal

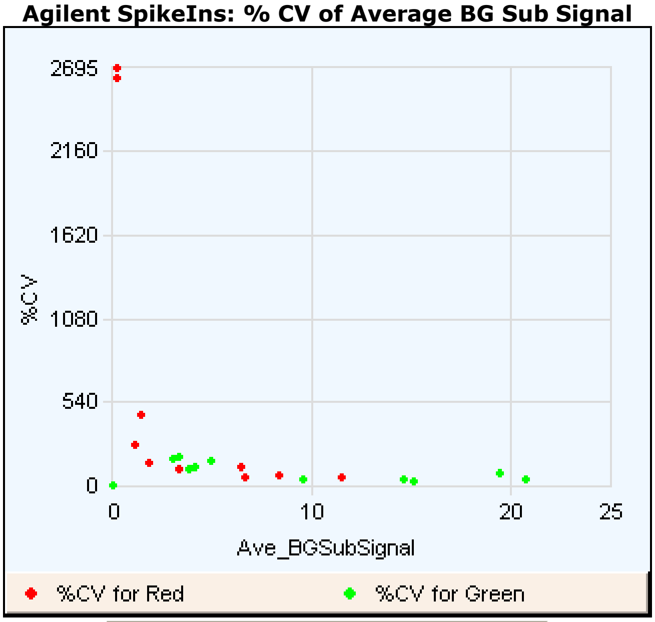

Median %CV:-1.00%(Red);-1.00%(Green)

Agilent SpikeIns: Expected LogRatio Vs Observed LogRatio

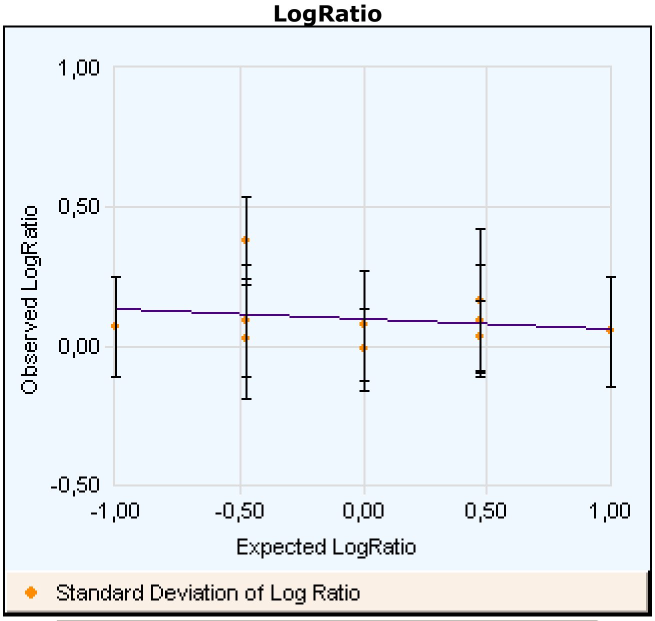

Y-Intercept = 0.097 ; Slope = -0.035 ; R^2 = 0.037

Human Retinal Pigment Epithelium Sample 1

QC Report - Agilent Technologies : 2 Color Gene Expression

Date	Thursday, April 16, 2015 - 09:35	BG Method	No Background
Image	US12302316_251485079962_S01_H [1_2]	Background Detrend	On(FeatNCRange, LoPass)
Protocol	GE2-v5_95_Feb07 (Read Only)	Multiplicative Detrend	True
User Name	bennis	Dye Norm	Linear Lowess
Grid	014850_D_20070207	Linear DyeNorm Factor	1.82(Red) 1.17(Green)
FE Version	9.5.3.1	Additive Error	7(Red)4(Green)
		Saturation Value	593997 (r), 584507 (g)

Spot Finding of the Four Corners of the Array

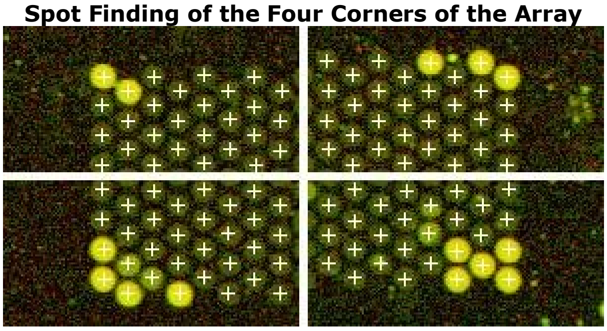

Grid Normal

	Feature Red	Feature Green	Local Background Red	Local Background Green
Non Uniform	144	226	88	92
Population	208	184	1476	1327

Spatial Distribution of All Outliers on the Array

532 rows x 85 columns

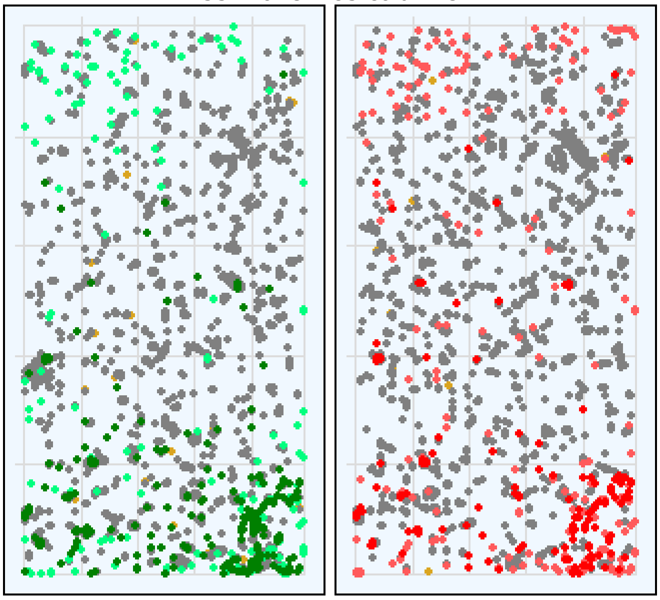

FeatureNonUnif (Red or Green) = 251(0.56%)

GeneNonUnif (Red or Green) = 217 (0.529 %)

- BG NonUniform
- BG Population
- Red FeaturePopulation
- Red Feature NonUniform
- Green FeaturePopulation
- Green Feature NonUniform

Negative Control Stats

	Red	Green
Average Net Signals	43.03	48.27
StdDev Net Signals	3.00	2.74

Net Signal Statistics

Agilent SpikeIns:

	Red	Green
# Saturated Features	0	0
99% of Sig. Distrib.	186	295
50% of Sig. Distrib.	75	71
1% of Sig. Distrib.	46	52

Non-Control probes:

	Red	Green
# Saturated Features	0	0
99% of Sig. Distrib.	48738	65226
50% of Sig. Distrib.	285	509
1% of Sig. Distrib.	48	54

Red and Green Background Corrected Signals (Non-Control Inliers)

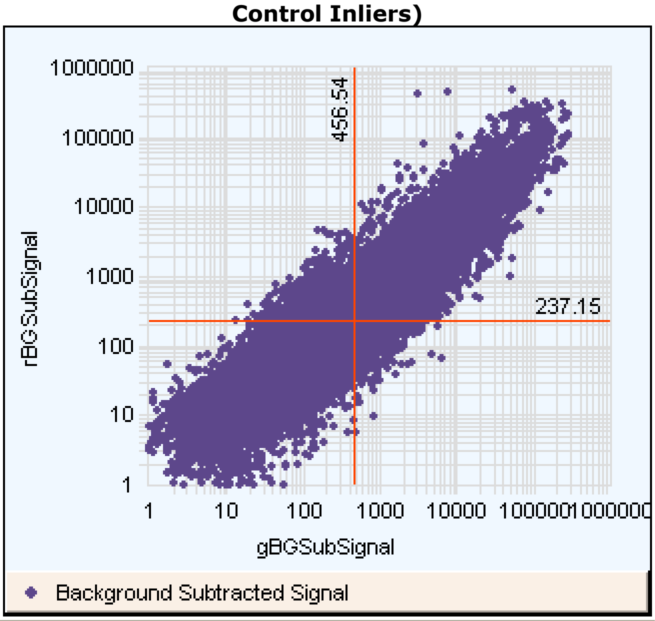

Features (NonCtrl) with BGSubSignals < 0: 437 (Red); 276 (Green)

	Red	Green
Average BG Sub Signal	-5.08	-4.03
StdDev BG Sub Signal	2.67	2.50

Local Bkg (inliers)

	Red	Green
Number	43527	43668
Avg	41.03	35.32
SD	2.19	1.94

Foreground Surface Fit

	Red	Green
RMS_Fit	1.36	1.25
RMS_Resid	4.09	3.16
Avg_Fit	64.47	69.02

Reproducibility: %CV for Replicated Probes

	Median %CV Signal (inliers)			
	Non-Control probes		Agilent SpikeIns	
	Red	Green	Red	Green
BGSubSignal	13.82	12.96	-1.00	-1.00
ProcessedSignal	5.31	4.97	-1.00	-1.00

Array Uniformity: LogRatios

	Non-Control	Agilent SpikeIns
AbsAvgLogRatio	0.27	0.18
AverageS/N	16.09	1.92

Sensitivity:Agilent SpikeIns - Ratio of Signal to Background for 2 dimmest probes

(+)E1A_r60_n11		(+)E1A_r60_a97	
**(g)**	**(r)**	**(g)**	**(r)**
1.7	1.4	1.5	1.3

Spatial Distribution of Significantly Up-Regulated and Down-Regulated Features

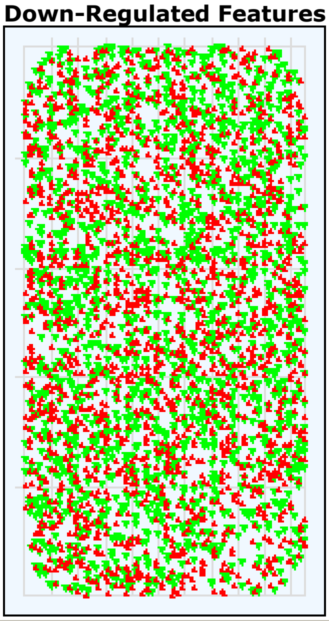

#Up-Regulated:10317 (Red) ; #Down-Regulated:10857 (Green)

Up-Regulated Down-Regulated

LogRatio Versus Log Processed Signal

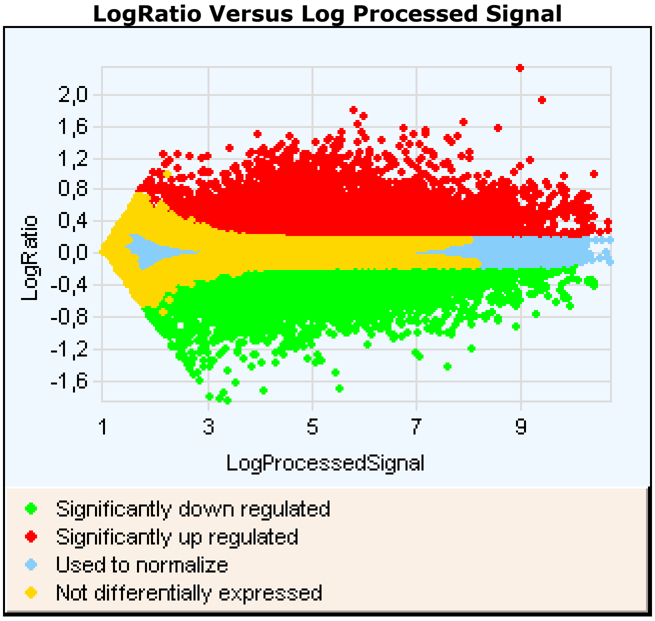

Agilent SpikeIns Signal Statistics

Probe Name	Exp	Obs	SD	S/N
(+)E1A_r60_n9	-1.00	0.14	0.10	1.38
(+)E1A_r60_a107	-0.48	0.26	0.09	3.01
(+)E1A_r60_a135	-0.48	0.14	0.13	1.08
(+)E1A_r60_n11	-0.48	0.26	0.09	2.87
(+)E1A_r60_1	0.00	-0.07	0.10	0.68
(+)E1A_r60_a20	0.00	0.17	0.08	2.16
(+)E1A_r60_3	0.48	0.09	0.08	1.15
(+)E1A_r60_a104	0.48	0.21	0.12	1.72
(+)E1A_r60_a97	0.48	0.24	0.12	1.95
(+)E1A_r60_a22	1.00	0.19	0.06	3.16

Agilent SpikeIns: % CV of Average BG Sub Signal

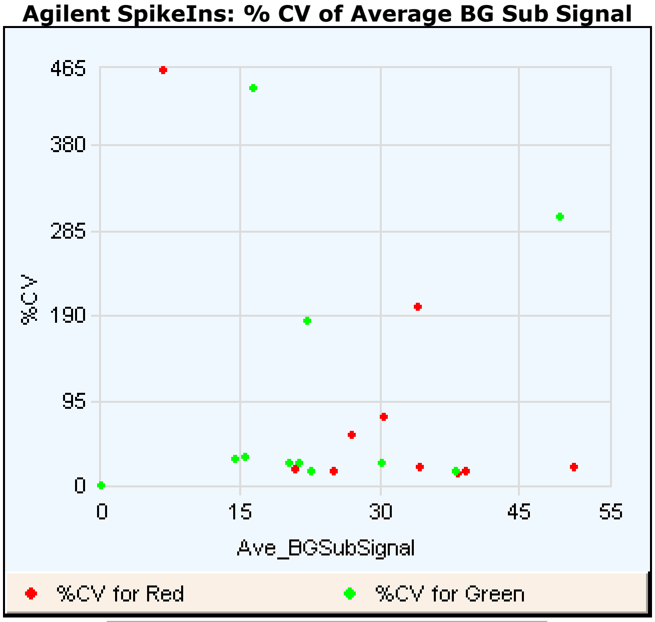

Median %CV:-1.00%(Red);-1.00%(Green)

Agilent SpikeIns: Expected LogRatio Vs Observed LogRatio

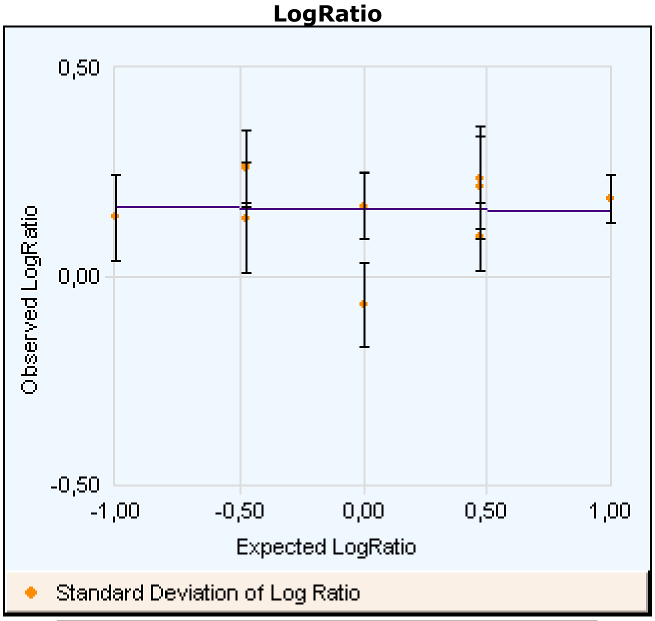

Y-Intercept = 0.165 ; Slope = -0.004 ; R^2 = 0.000

Human Retinal Pigment Epithelium Sample 2

QC Report - Agilent Technologies : 2 Color Gene Expression

Date	Thursday, April 16, 2015 - 09:42	BG Method	No Background
Image	US12302316_251485079962_S01_H [1_4]	Background Detrend	On(FeatNCRange, LoPass)
Protocol	GE2-v5_95_Feb07 (Read Only)	Multiplicative Detrend	True
User Name	bennis	Dye Norm	Linear Lowess
Grid	014850_D_20070207	Linear DyeNorm Factor	1.84(Red) 1.41(Green)
FE Version	9.5.3.1	Additive Error	4(Red)4(Green)
		Saturation Value	591262 (r), 583338 (g)

Spot Finding of the Four Corners of the Array

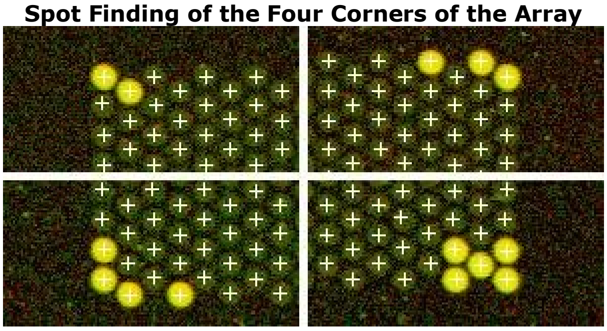

Grid Normal

	Feature		Local Background	
	Red	Green	Red	Green
Non Uniform	7	13	120	13
Population	92	78	1504	852

Spatial Distribution of All Outliers on the Array

532 rows x 85 columns

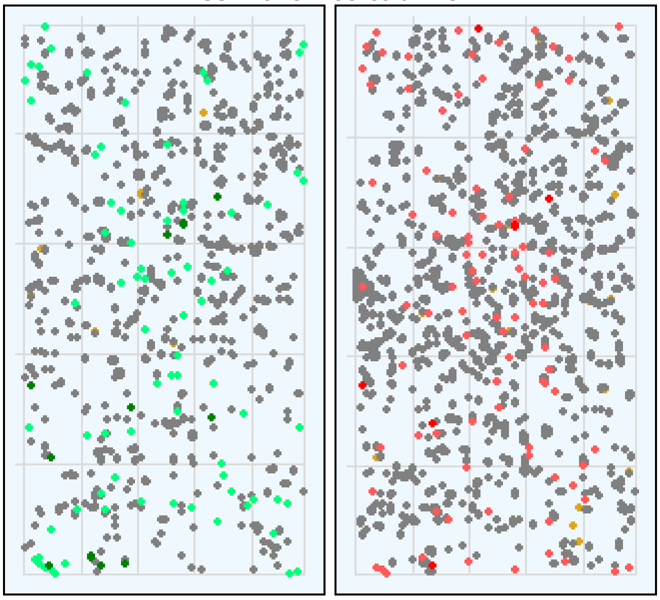

FeatureNonUnif (Red or Green) = 16(0.04%)

GeneNonUnif (Red or Green) = 15 (0.037 %)

BG NonUniform
BG Population
Red FeaturePopulation
Red Feature NonUniform
Green FeaturePopulation
Green Feature NonUniform

Negative Control Stats

	Red	Green
Average Net Signals	35.62	44.19
StdDev Net Signals	2.21	2.23

Net Signal Statistics

Agilent SpikeIns:

	Red	Green
# Saturated Features	0	0
99% of Sig. Distrib.	73	77
50% of Sig. Distrib.	46	56
1% of Sig. Distrib.	36	45

Non-Control probes:

	Red	Green
# Saturated Features	0	0
99% of Sig. Distrib.	78521	57539
50% of Sig. Distrib.	303	524
1% of Sig. Distrib.	38	50

Red and Green Background Corrected Signals (Non-Control Inliers)

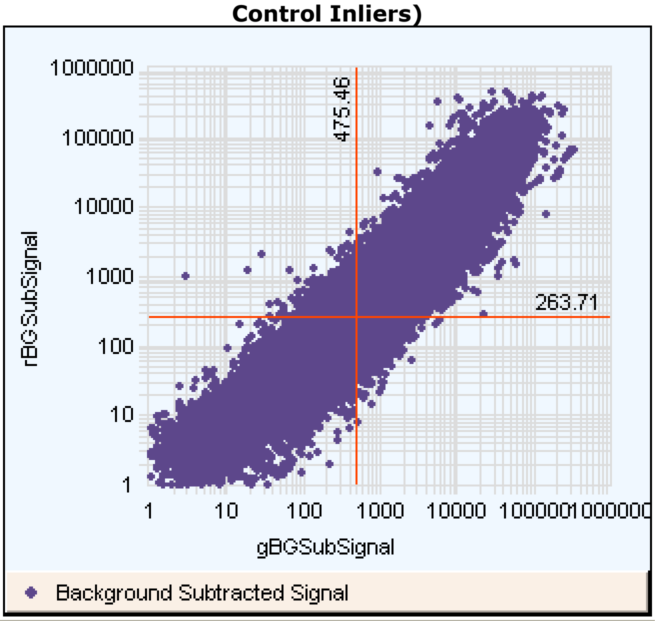

Features (NonCtrl) with BGSubSignals < 0: 644 (Red); 244 (Green)

Average BG Sub Signal	-3.56	-3.97
StdDev BG Sub Signal	2.14	2.25

Local Bkg (inliers)

	Red	Green
Number	43486	44156
Avg	40.73	37.21
SD	2.31	1.88

Foreground Surface Fit

	Red	Green
RMS_Fit	0.68	0.67
RMS_Resid	2.32	2.62
Avg_Fit	55.49	64.87

Reproducibility: %CV for Replicated Probes

Median %CV Signal (inliers)

	Non-Control probes		Agilent SpikeIns	
	Red	Green	Red	Green
BGSubSignal	13.43	12.93	-1.00	-1.00
ProcessedSignal	4.82	5.43	-1.00	-1.00

Array Uniformity: LogRatios

	Non-Control	Agilent SpikeIns
AbsAvgLogRatio	0.31	0.12
AverageS/N	21.31	0.81

Sensitivity:Agilent SpikeIns - Ratio of Signal to Background for 2 dimmest probes

(+)E1A_r60_n11		(+)E1A_r60_a97	
**(g)**	**(r)**	**(g)**	**(r)**
1.4	1.3	1.0	1.1

Spatial Distribution of Significantly Up-Regulated and Down-Regulated Features

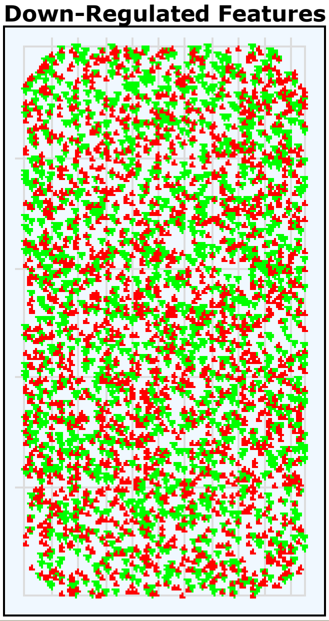

#Up-Regulated:12514 (Red) ; #Down-Regulated:11268 (Green)

Up-Regulated Down-Regulated

LogRatio Versus Log Processed Signal

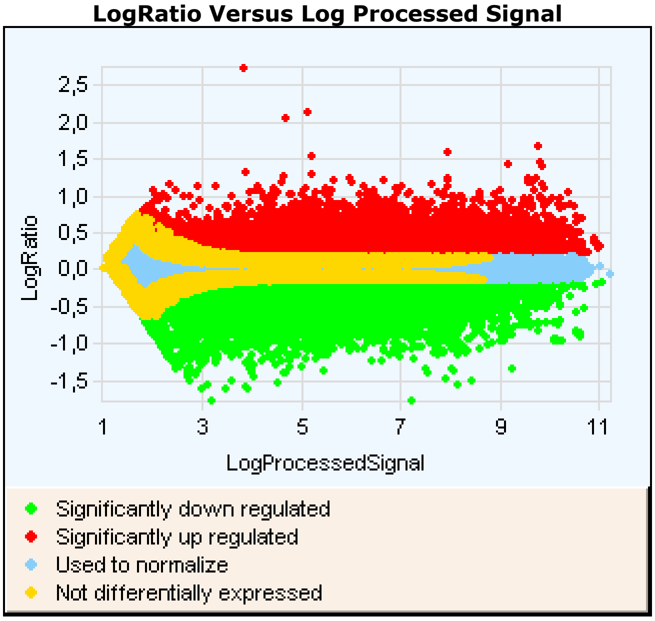

Agilent SpikeIns Signal Statistics

Probe Name	Exp	Obs	SD	S/N
(+)E1A_r60_n9	-1.00	0.01	0.23	0.06
(+)E1A_r60_a107	-0.48	0.12	0.18	0.65
(+)E1A_r60_a135	-0.48	0.12	0.23	0.52
(+)E1A_r60_n11	-0.48	0.22	0.12	1.79
(+)E1A_r60_1	0.00	0.12	0.24	0.49
(+)E1A_r60_a20	0.00	0.25	0.40	0.62
(+)E1A_r60_3	0.48	0.09	0.14	0.66
(+)E1A_r60_a104	0.48	0.09	0.24	0.40
(+)E1A_r60_a97	0.48	-0.01	0.17	0.07
(+)E1A_r60_a22	1.00	0.21	0.07	2.80

Agilent SpikeIns: % CV of Average BG Sub Signal

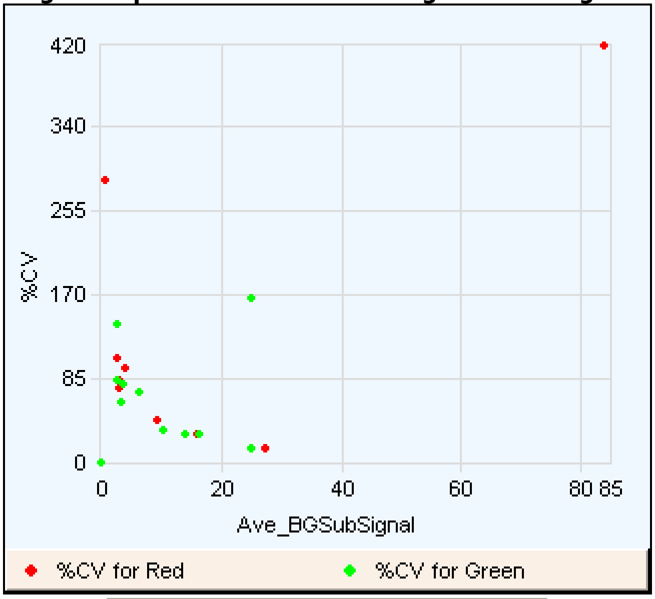

Median %CV:-1.00%(Red);-1.00%(Green)

Agilent SpikeIns: Expected LogRatio Vs Observed LogRatio

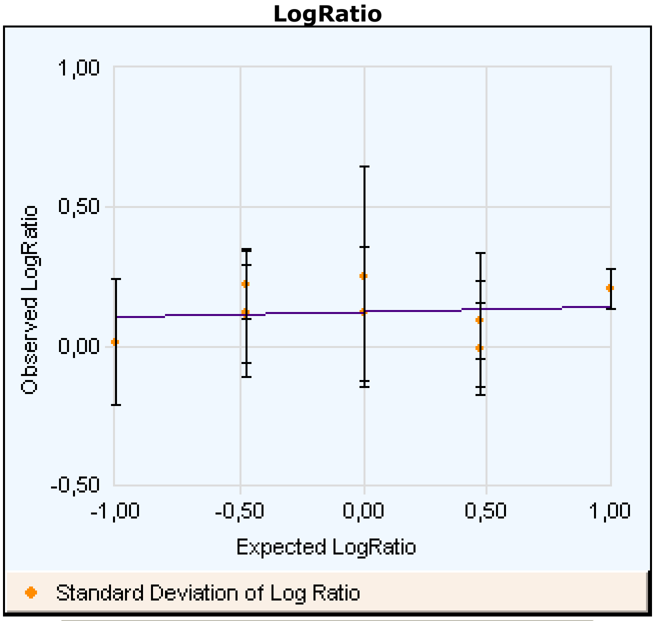

Y-Intercept = 0.122 ; Slope = 0.018 ; R^2 = 0.017

Human Retinal Pigment Epithelium Sample 3

QC Report - Agilent Technologies : 2 Color Gene Expression

Date	Thursday, April 16, 2015 - 09:31	BG Method	No Background
Image	US12302316_251485079962_S01_H [1_1]	Background Detrend	On(FeatNCRange, LoPass)
Protocol	GE2-v5_95_Feb07 (Read Only)	Multiplicative Detrend	True
User Name	bennis	Dye Norm	Linear Lowess
Grid	014850_D_20070207	Linear DyeNorm Factor	1.6(Red) 1.84(Green)
FE Version	9.5.3.1	Additive Error	4(Red)4(Green)
		Saturation Value	602397 (r), 583471 (g)

Spot Finding of the Four Corners of the Array

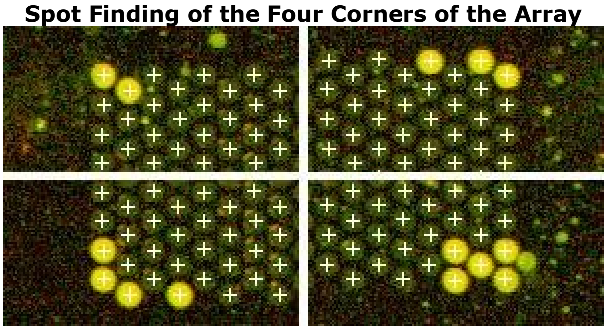

Grid Normal

	Feature Red	Feature Green	Local Background Red	Local Background Green
Non Uniform	13	14	278	52
Population	114	87	1919	1090

Spatial Distribution of All Outliers on the Array

532 rows x 85 columns

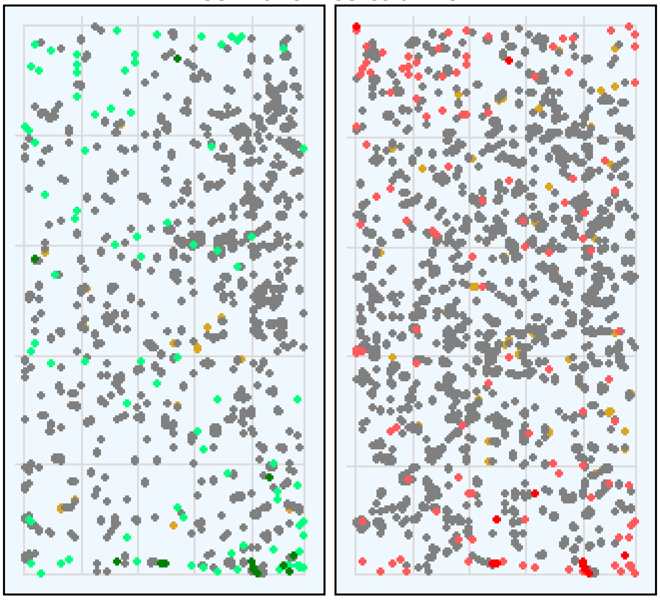

FeatureNonUnif (Red or Green) = 18(0.04%)

GeneNonUnif (Red or Green) = 15 (0.037 %)

- BG NonUniform
- BG Population
- Red FeaturePopulation
- Red Feature NonUniform
- Green FeaturePopulation
- Green Feature NonUniform

Negative Control Stats

	Red	Green
Average Net Signals	36.40	39.09
StdDev Net Signals	2.20	2.33

Net Signal Statistics

Agilent SpikeIns:

	Red	Green
# Saturated Features	0	0
99% of Sig. Distrib.	91	77
50% of Sig. Distrib.	50	49
1% of Sig. Distrib.	37	39

Non-Control probes:

	Red	Green
# Saturated Features	2	0
99% of Sig. Distrib.	109525	50344
50% of Sig. Distrib.	428	477
1% of Sig. Distrib.	40	43

Red and Green Background Corrected Signals (Non-Control Inliers)

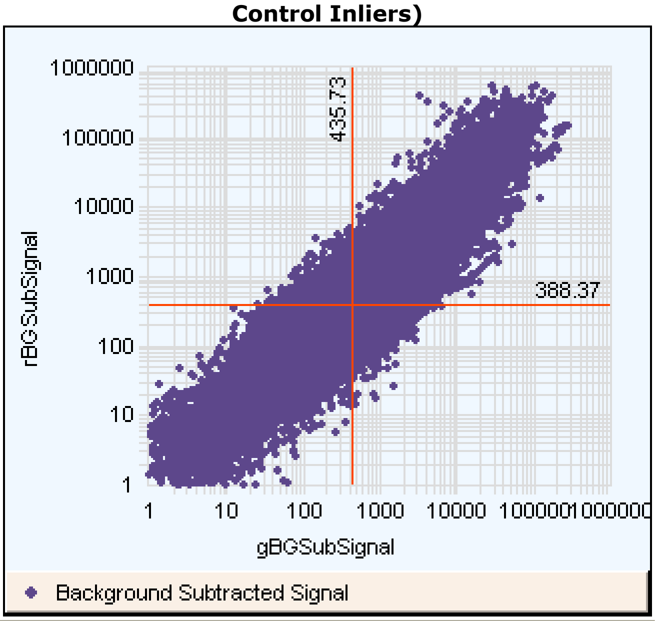

Features (NonCtrl) with BGSubSignals < 0: 414 (Red); 228 (Green)

Average BG Sub Signal	-3.49	-2.69
StdDev BG Sub Signal	2.17	2.06

Local Bkg (inliers)

	Red	Green
Number	43034	43900
Avg	41.53	31.75
SD	2.66	1.86

Foreground Surface Fit

	Red	Green
RMS_Fit	0.49	1.54
RMS_Resid	2.57	2.40
Avg_Fit	56.20	58.47

Reproducibility: %CV for Replicated Probes

Median %CV Signal (inliers)

	Non-Control probes		Agilent SpikeIns	
	Red	Green	Red	Green
BGSubSignal	13.10	13.12	-1.00	-1.00
ProcessedSignal	4.59	4.70	-1.00	-1.00

Array Uniformity: LogRatios

	Non-Control	Agilent SpikeIns
AbsAvgLogRatio	0.33	0.10
AverageS/N	23.96	0.93

Sensitivity:Agilent SpikeIns - Ratio of Signal to Background for 2 dimmest probes

(+)E1A_r60_n11		(+)E1A_r60_a97	
**(g)**	**(r)**	**(g)**	**(r)**
1.6	1.3	1.0	1.1

Spatial Distribution of Significantly Up-Regulated and Down-Regulated Features

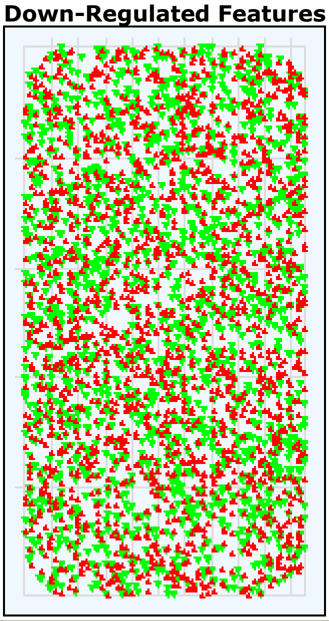

#Up-Regulated:11889 (Red) ; #Down-Regulated:11011 (Green)

Up-Regulated Down-Regulated

LogRatio Versus Log Processed Signal

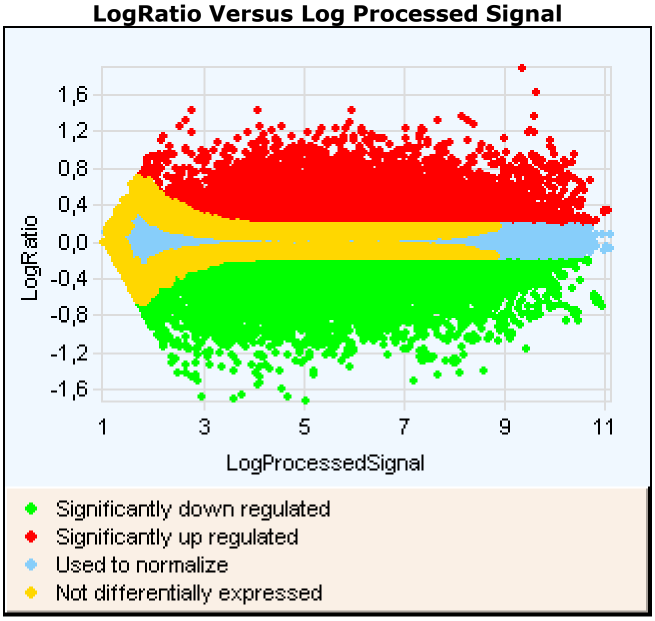

Agilent SpikeIns Signal Statistics

Probe Name	Exp	Obs	SD	S/N
(+)E1A_r60_n9	-1.00	0.02	0.18	0.13
(+)E1A_r60_a107	-0.48	0.06	0.11	0.54
(+)E1A_r60_a135	-0.48	0.05	0.19	0.28
(+)E1A_r60_n11	-0.48	0.25	0.14	1.81
(+)E1A_r60_1	0.00	0.07	0.18	0.41
(+)E1A_r60_a20	0.00	0.16	0.10	1.66
(+)E1A_r60_3	0.48	0.08	0.10	0.82
(+)E1A_r60_a104	0.48	0.03	0.23	0.12
(+)E1A_r60_a97	0.48	-0.08	0.20	0.39
(+)E1A_r60_a22	1.00	0.14	0.05	3.09

Agilent SpikeIns: % CV of Average BG Sub Signal

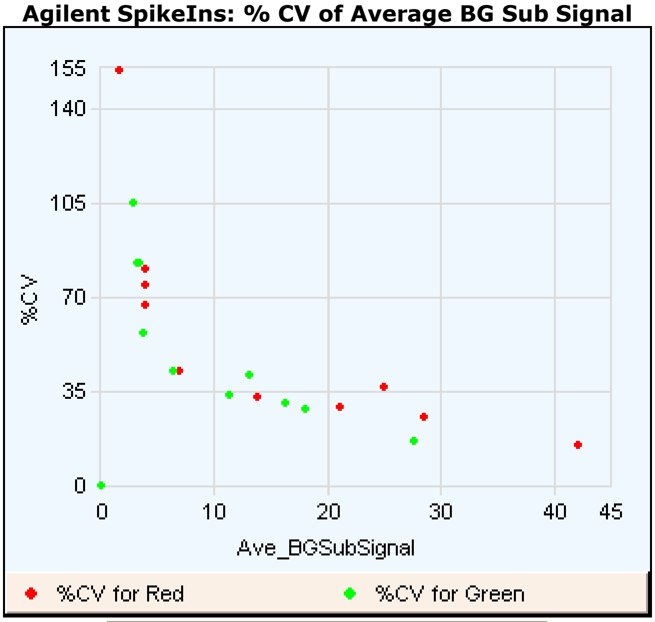

Median %CV:-1.00%(Red);-1.00%(Green)

Agilent SpikeIns: Expected LogRatio Vs Observed LogRatio

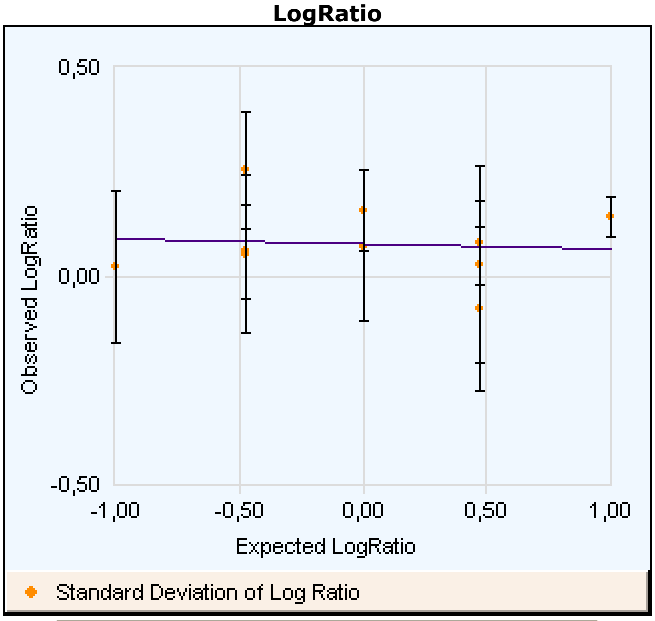

Y-Intercept = 0.080 ; Slope = -0.012 ; R^2 = 0.007

Human Retinal Pigment Epithelium Sample 4

QC Report - Agilent Technologies : 2 Color Gene Expression

Date	Thursday, April 16, 2015 - 09:38	BG Method	No Background
Image	US12302316_251485079962_S01_H [1_3]	Background Detrend	On(FeatNCRange, LoPass)
Protocol	GE2-v5_95_Feb07 (Read Only)	Multiplicative Detrend	True
User Name	bennis	Dye Norm	Linear Lowess
Grid	014850_D_20070207	Linear DyeNorm Factor	0.894(Red) 1.37(Green)
FE Version	9.5.3.1	Additive Error	10(Red)7(Green)
		Saturation Value	593071 (r), 583874 (g)

Spot Finding of the Four Corners of the Array

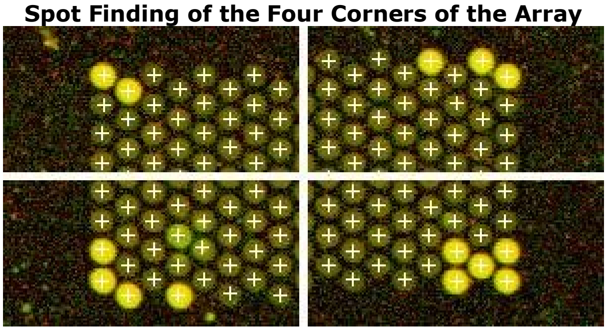

Grid Normal

	Feature Red	Feature Green	Local Background Red	Local Background Green
Non Uniform	53	58	288	59
Population	184	163	1742	919

Spatial Distribution of All Outliers on the Array

532 rows x 85 columns

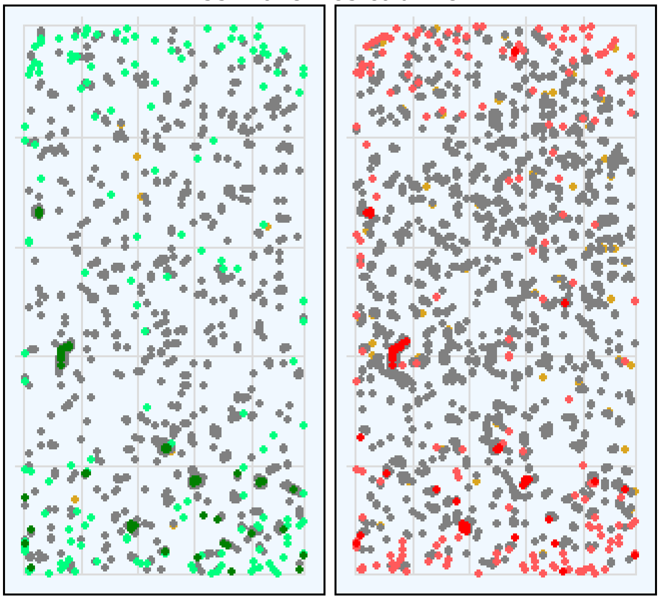

FeatureNonUnif (Red or Green) = 77(0.17%)

GeneNonUnif (Red or Green) = 66 (0.161 %)

BG NonUniform   BG Population

Red FeaturePopulation   Red Feature NonUniform

Green FeaturePopulation   Green Feature NonUniform

Negative Control Stats

	Red	Green
Average Net Signals	86.59	66.30
StdDev Net Signals	5.33	3.29

Net Signal Statistics

Agilent SpikeIns:

	Red	Green
# Saturated Features	0	0
99% of Sig. Distrib.	492	244
50% of Sig. Distrib.	325	174
1% of Sig. Distrib.	108	78

Non-Control probes:

	Red	Green
# Saturated Features	6	0
99% of Sig. Distrib.	143310	43519
50% of Sig. Distrib.	540	556
1% of Sig. Distrib.	100	78

Red and Green Background Corrected Signals (Non-Control Inliers)

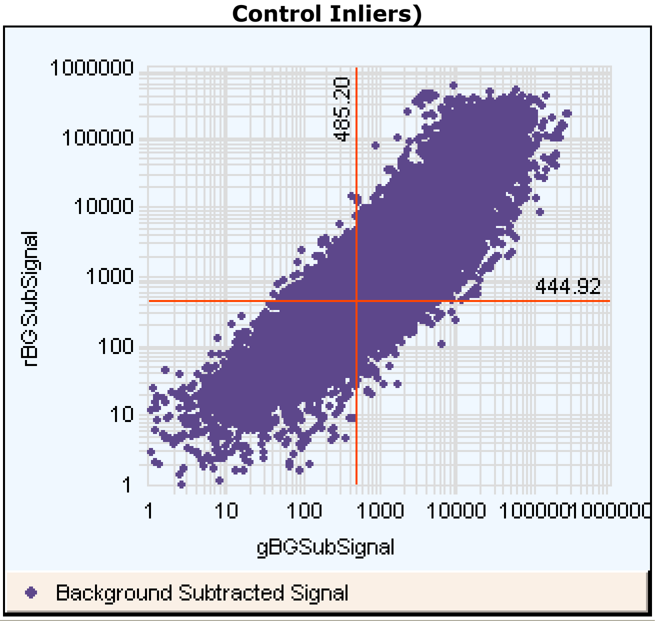

Features (NonCtrl) with BGSubSignals < 0: 298 (Red); 193 (Green)

Average BG Sub Signal	-7.19	-4.36
StdDev BG Sub Signal	4.93	3.48

Local Bkg (inliers)

	Red	Green
Number	43202	44087
Avg	42.88	36.48
SD	2.83	1.83

Foreground Surface Fit

	Red	Green
RMS_Fit	3.95	3.40
RMS_Resid	10.97	5.37
Avg_Fit	110.69	87.54

Reproducibility: %CV for Replicated Probes

	Median %CV Signal (inliers)			
	Non-Control probes		Agilent SpikeIns	
	Red	Green	Red	Green
BGSubSignal	11.30	11.04	9.27	8.67
ProcessedSignal	5.64	5.37	15.85	12.36

Array Uniformity: LogRatios

	Non-Control	Agilent SpikeIns
AbsAvgLogRatio	0.36	0.22
AverageS/N	21.49	6.29

Sensitivity:Agilent SpikeIns - Ratio of Signal to Background for 2 dimmest probes

(+)E1A_r60_n11		(+)E1A_r60_a97	
**(g)**	**(r)**	**(g)**	**(r)**
3.7	2.6	3.8	2.6

Spatial Distribution of Significantly Up-Regulated and Down-Regulated Features

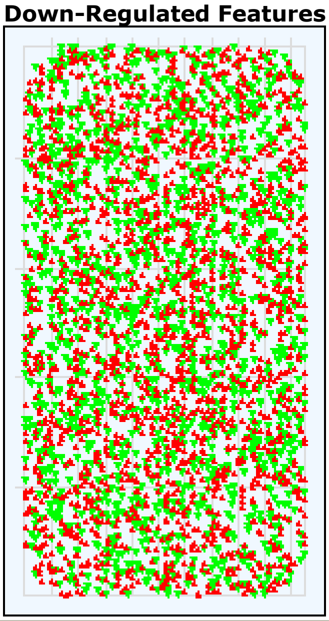

#Up-Regulated:12872 (Red) ; #Down-Regulated:11382 (Green)

Up-Regulated Down-Regulated

LogRatio Versus Log Processed Signal

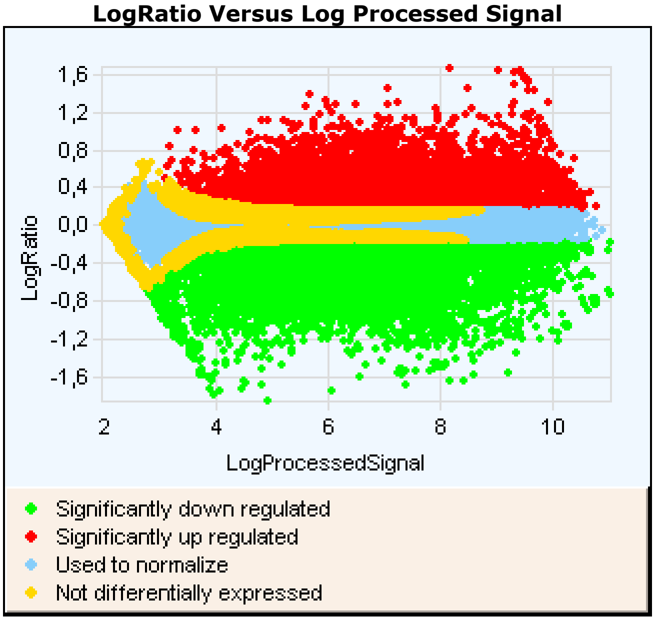

Agilent SpikeIns Signal Statistics

Probe Name	Exp	Obs	SD	S/N
(+)E1A_r60_n9	-1.00	0.21	0.09	2.31
(+)E1A_r60_a107	-0.48	0.26	0.03	9.80
(+)E1A_r60_a135	-0.48	0.21	0.03	6.33
(+)E1A_r60_n11	-0.48	0.23	0.11	2.03
(+)E1A_r60_1	0.00	0.06	0.17	0.35
(+)E1A_r60_a20	0.00	0.30	0.03	9.79
(+)E1A_r60_3	0.48	0.15	0.09	1.58
(+)E1A_r60_a104	0.48	0.26	0.02	12.26
(+)E1A_r60_a97	0.48	0.27	0.03	10.53
(+)E1A_r60_a22	1.00	0.23	0.03	7.87

Agilent SpikeIns: % CV of Average BG Sub Signal

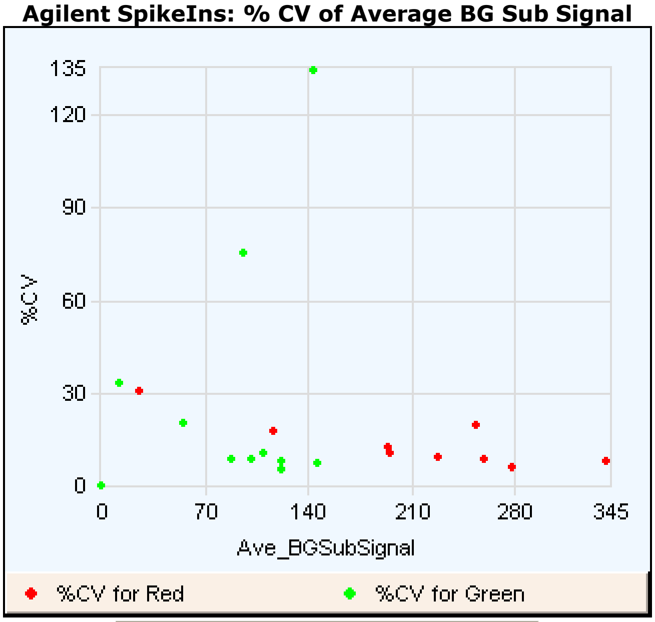

Median %CV:9.27%(Red);8.67%(Green)

Agilent SpikeIns: Expected LogRatio Vs Observed LogRatio

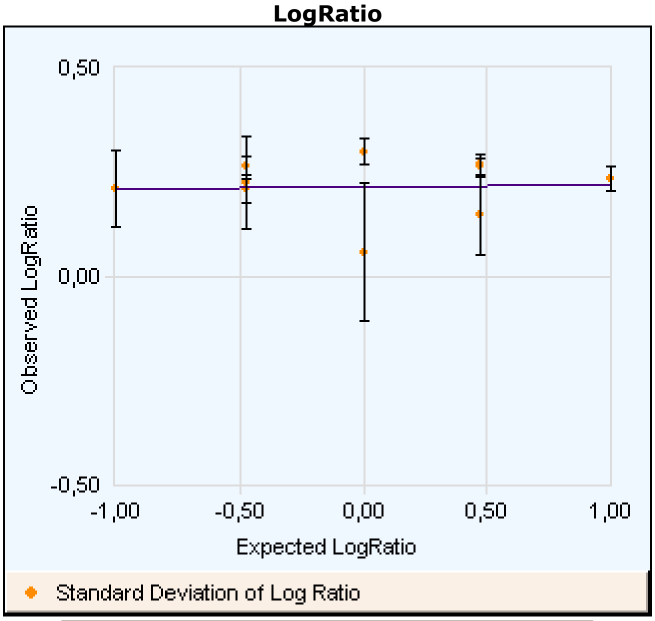

Y-Intercept = 0.218 ; Slope = 0.004 ; R^2 = 0.001

Human Retinal Pigment Epithelium Sample 5

QC Report - Agilent Technologies : 2 Color Gene Expression

Date	Thursday, April 16, 2015 - 11:22	BG Method	No Background
Image	US12302316_251485079963_S01_H [1_1]	Background Detrend	On(FeatNCRange, LoPass)
Protocol	GE2-v5_95_Feb07 (Read Only)	Multiplicative Detrend	True
User Name	bennis	Dye Norm	Linear Lowess
Grid	014850_D_20070207	Linear DyeNorm Factor	5.11(Red)0.894(Green)
FE Version	9.5.3.1	Additive Error	12(Red)2(Green)
		Saturation Value	578643 (r), 588759 (g)

Spot Finding of the Four Corners of the Array

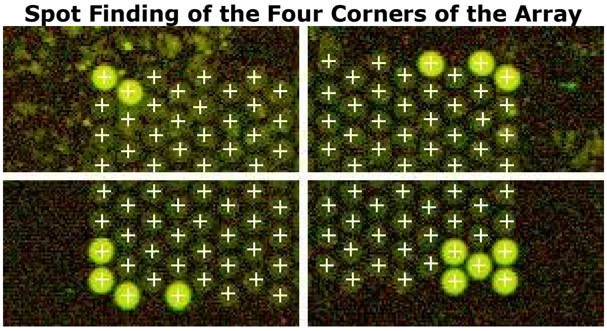

Grid Normal

	Feature		Local Background	
	Red	Green	Red	Green
Non Uniform	50	62	40	275
Population	163	183	839	1169

Spatial Distribution of All Outliers on the Array

532 rows x 85 columns

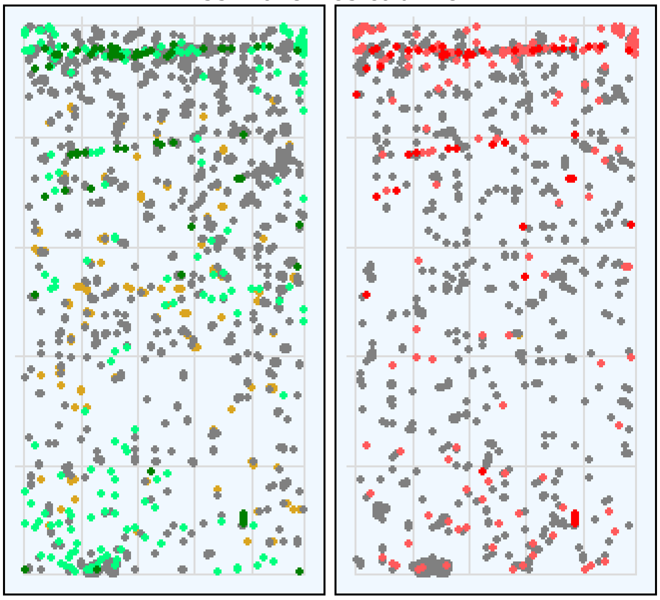

FeatureNonUnif (Red or Green) = 72(0.16%)

GeneNonUnif (Red or Green) = 57 (0.139 %)

● BG NonUniform ● BG Population

● Red FeaturePopulation ● Red Feature NonUniform

● Green FeaturePopulation ● Green Feature NonUniform

Negative Control Stats

	Red	Green
Average Net Signals	34.51	31.87
StdDev Net Signals	2.63	3.13

Net Signal Statistics

Agilent SpikeIns:

	Red	Green
# Saturated Features	0	0
99% of Sig. Distrib.	53	80
50% of Sig. Distrib.	38	43
1% of Sig. Distrib.	30	32

Non-Control probes:

	Red	Green
# Saturated Features	0	0
99% of Sig. Distrib.	14688	65019
50% of Sig. Distrib.	91	432
1% of Sig. Distrib.	33	35

Red and Green Background Corrected Signals (Non-Control Inliers)

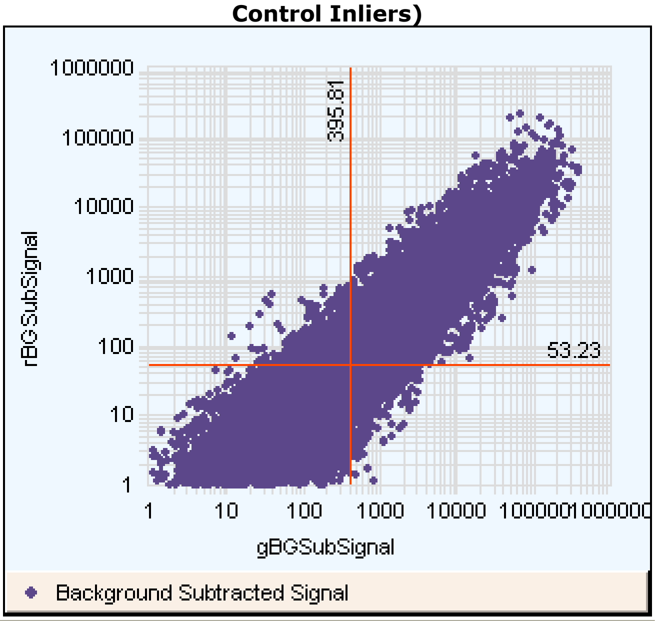

Features (NonCtrl) with BGSubSignals < 0: 3318 (Red); 529 (Green)

Average BG Sub Signal	-2.95	-3.99
StdDev BG Sub Signal	2.12	2.09

Local Bkg (inliers)

	Red	Green
Number	44175	43701
Avg	42.42	29.83
SD	2.25	1.99

Foreground Surface Fit

	Red	Green
RMS_Fit	1.79	2.59
RMS_Resid	2.36	2.63
Avg_Fit	54.58	53.09

Reproducibility: %CV for Replicated Probes

	Median %CV Signal (inliers)			
	Non-Control probes		Agilent SpikeIns	
	Red	Green	Red	Green
BGSubSignal	21.39	7.28	-1.00	-1.00
ProcessedSignal	7.04	3.78	-1.00	-1.00

Array Uniformity: LogRatios

	Non-Control	Agilent SpikeIns
AbsAvgLogRatio	0.32	0.03
AverageS/N	10.04	0.23

Sensitivity:Agilent SpikeIns - Ratio of Signal to Background for 2 dimmest probes

(+)E1A_r60_n11		(+)E1A_r60_a97	
**(g)**	**(r)**	**(g)**	**(r)**
1.0	1.4	0.9	1.0

Spatial Distribution of Significantly Up-Regulated and Down-Regulated Features

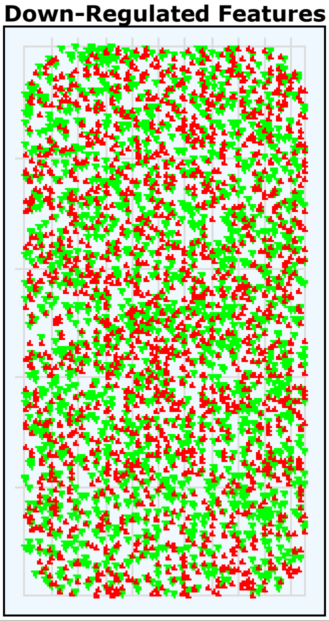

#Up-Regulated:10268 (Red) ; #Down-Regulated:10846 (Green)

Up-Regulated Down-Regulated

LogRatio Versus Log Processed Signal

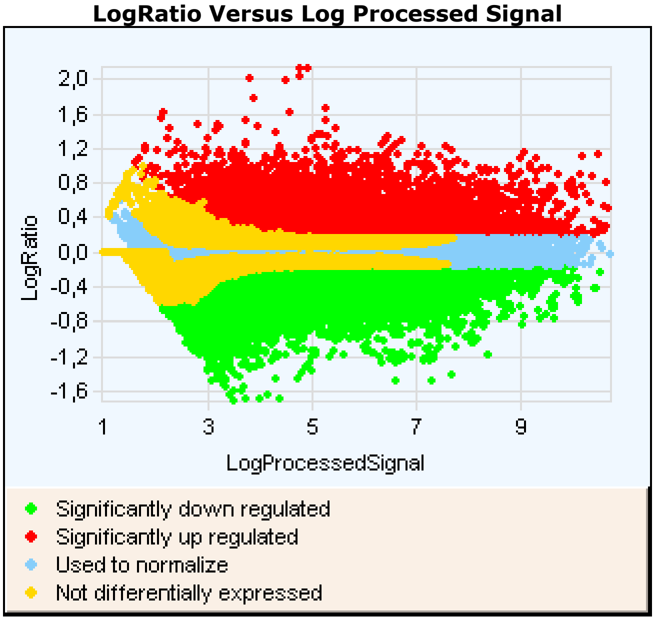

Agilent SpikeIns Signal Statistics

Probe Name	Exp	Obs	SD	S/N
(+)E1A_r60_n9	-1.00	0.03	0.10	0.25
(+)E1A_r60_a107	-0.48	-0.01	0.12	0.07
(+)E1A_r60_a135	-0.48	0.02	0.12	0.18
(+)E1A_r60_n11	-0.48	-0.03	0.17	0.16
(+)E1A_r60_1	0.00	0.03	0.13	0.25
(+)E1A_r60_a20	0.00	0.04	0.23	0.17
(+)E1A_r60_3	0.48	-0.05	0.16	0.30
(+)E1A_r60_a104	0.48	0.01	0.05	0.18
(+)E1A_r60_a97	0.48	0.01	0.07	0.18
(+)E1A_r60_a22	1.00	0.10	0.19	0.51

Agilent SpikeIns: % CV of Average BG Sub Signal

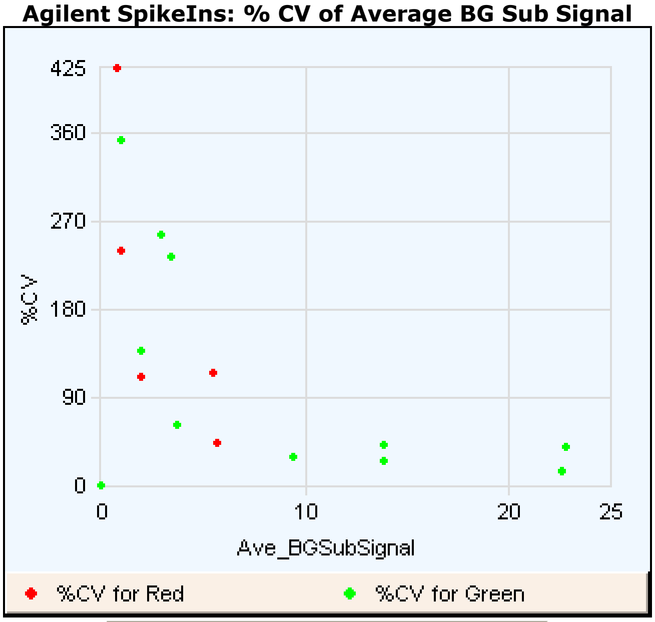

Median %CV:-1.00%(Red);-1.00%(Green)

Agilent SpikeIns: Expected LogRatio Vs Observed LogRatio

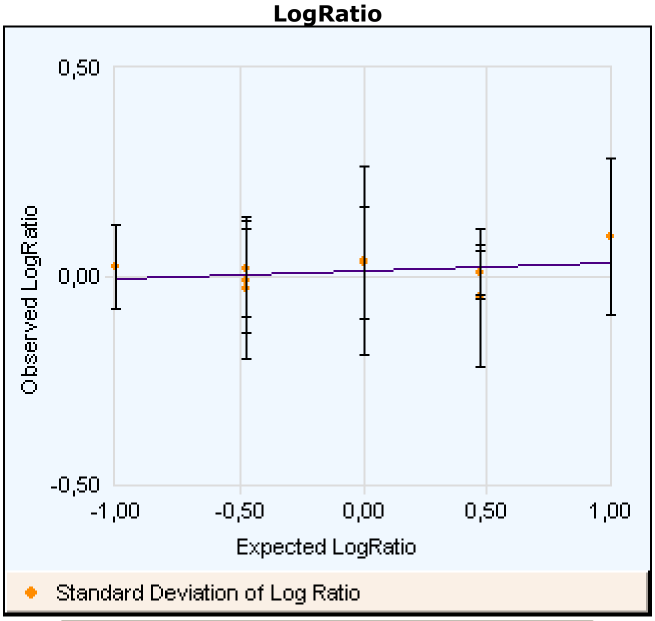

Y-Intercept = 0.015 ; Slope = 0.019 ; R^2 = 0.086
